# The synergism of cytosolic acidosis and reduced NAD^+^/NADH ratio is responsible for lactic acidosis-induced vascular smooth muscle cell impairment in sepsis

**DOI:** 10.1186/s12929-023-00992-6

**Published:** 2024-01-09

**Authors:** Philipp Terpe, Stefanie Ruhs, Virginie Dubourg, Michael Bucher, Michael Gekle

**Affiliations:** 1 Julius-Bernstein-Institute of Physiology, Martin Luther University Halle-Wittenberg, 06112 Halle (Saale), Germany; 2 Department of Anesthesiology and Surgical Intensive Care, University Hospital Halle (Saale), 06120 Halle (Saale), Germany

**Keywords:** Sepsis, Metabolism, Lactic acidosis, Acidosis, Vascular smooth muscle cells, NAD^+^/NADH ratio, Cellular dedifferentiation, Vascular calcification

## Abstract

**Background:**

During sepsis, serve vascular dysfunctions lead to life-threatening multiple organ failure, due to vascular smooth muscle cells (VSMC) impairments, resulting in vasoplegia, hypotension and hypoperfusion. In addition, septic patients have an altered cell metabolism that leads to lactic acidosis. Septic patients suffering from lactic acidosis have a high risk of mortality. In addition, septic survivors are at risk of secondary vascular disease. The underlying mechanisms of whether and how lactic acidosis leads to the changes in VSMCs is not well understood. The aim of this study was to comprehensively investigate the effect of lactic acidosis on VSMCs and additionally compare the effects with those induced by pure acidosis and sodium lactate.

**Methods:**

Primary human aortic smooth muscle cells (HAoSMCs) were treated for 48 h with lactic acidosis (LA_pH 6.8), hydrochloric acid (HCl_pH 6.8), sodium lactate (Na^+^-lactate_pH 7.4) and the respective controls (ctrl._pH 7.4; hyperosmolarity control: mannitol_pH 7.4) and comparatively analyzed for changes in (i) transcriptome, (ii) energy metabolism, and (iii) phenotype.

**Results:**

Both types of acidosis led to comparable and sustained intracellular acidification without affecting cell viability. RNA sequencing and detailed transcriptome analysis revealed more significant changes for lactic acidosis than for hydrochloric acidosis, with lactate being almost ineffective, suggesting qualitative and quantitative synergism of acidosis and lactate. Bioinformatic predictions in energy metabolism and phenotype were confirmed experimentally. Lactic acidosis resulted in strong inhibition of glycolysis, glutaminolysis, and altered mitochondrial respiration which reduced cellular ATP content, likely due to increased TXNIP expression and altered NAD^+^/NADH ratio. Hydrochloric acidosis induced significantly smaller effects without changing the NAD^+^/NADH ratio, with the ATP content remaining constant. These metabolic changes led to osteo-/chondrogenic/senescent transdifferentiation of VSMCs, with the effect being more pronounced in lactic acidosis than in pure acidosis.

**Conclusions:**

Overall, lactic acidosis exerted a much stronger effect on energy metabolism than pure acidosis, whereas lactate had almost no effect, reflecting the qualitative and quantitative synergism of acidosis and lactate. As a consequence, lactic acidosis may lead to acute functional impairments of VSMC, sustained perturbations of the transcriptome and cellular dedifferentiation. Moreover, these effects may contribute to the acute and prolonged vascular pathomechanisms in septic patients.

**Supplementary Information:**

The online version contains supplementary material available at 10.1186/s12929-023-00992-6.

## Background

Sepsis is a life-threatening organ dysfunction caused by a dysregulated inflammatory host response to an infection with bacteria or fungi or to their toxins. It is characterized by hemodynamic dysfunction of one or more organs [1] and a generalized vasodilation and a decreased systemic vascular resistance [2]. Thus, sepsis is a complex clinical and biochemical condition. It is one of the most common causes of death in intensive care medicine with a mortality rate of 30–50% [3] and its worldwide incidence still increases [3]. Although a number of effective treatment methods are now available in the context of focal rehabilitation and intensive care medicine, they remain predominantly non-specific and supportive [4]. This is due to the still incomplete knowledge of the causative cellular mechanisms leading to vascular malfunctions, which is one of the initial causes of sepsis-related systemic disease and multi-organ failure.

**Fig. 1:**
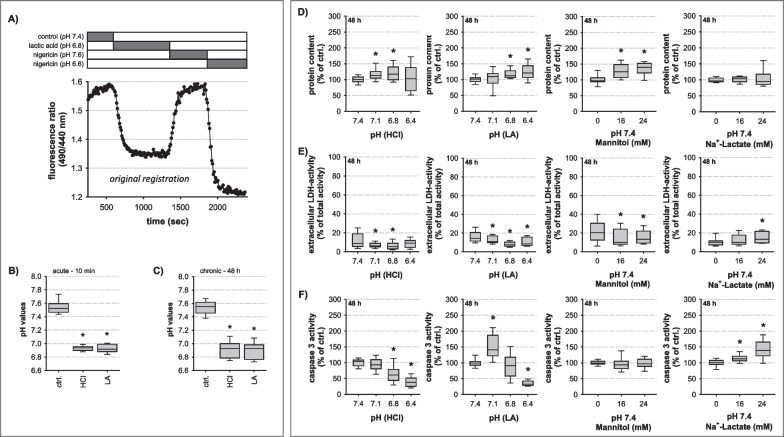
Influence of hydrochloric- and lactic acidosis on pH_i_ and cellular viability. **A** pH_i_ was determined by BCECF measurements. The BCECF fluorescence ratio was calibrated by using two nigericin solutions of known H^+^ concentration. Short-term (10 min) (**B**; N = 4; n = 42–93; *p ≤ 0.0001 vs. ctrl.) and chronic extracellular acidification (48 h) (**C**; N = 3; n = 35–42; *p ≤ 0.0001 vs. ctrl.) led to rapid and sustained intracellular acidification to a similar extent. Cellular viability was determined by protein content (**D**), LDH release (**E**), and caspase activity (**F**) for 48 h. Both types of acidosis did not decrease cellular protein content (N = 4–7, n = 15–21), nor induce necrotic (N = 4–8, n = 15–24) or apoptotic cell death (N = 3–7, n = 12–21); *p ≤ 0.01 vs. ctrl

**Fig. 2:**
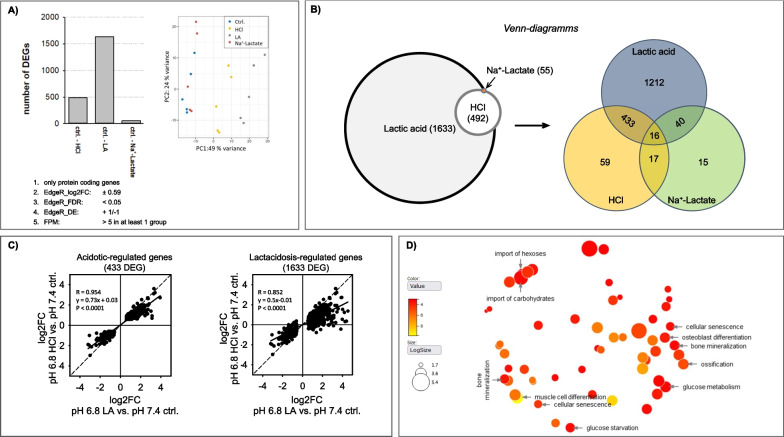
Acidosis impact on the transcriptome. **A** Differential expression analysis results for HAoSMCs treated with hydrochloric-/, lactic acidosis and Na^+^-Lactate for 48 h. Genes were considered as differentially expressed if |log2FC|≥ 0.59 and FDR ≤ 0.05 in the output of EdgeR. The average number of FPM had to be > 5 in at least one group. PCA were performed for all samples. The variation between sample groups was mostly related to the different pH treatment conditions (pH 7.4 vs. pH 6.8) whereby the type of acidosis was also crucial (PC1; N = 5). Lactic acidosis led to a more—1633 DEGs than hydrochloric acidosis—492 DEG. **B** The Venn diagram shows the number of genes regulated specifically and overlapping by hydrochloric acidosis (specific genes: 59), lactic acidosis (specific genes: 1212 genes), and Na^+^-Lactate (specific genes: 15). 433 genes are regulated by both hydrochloric- and lactic acidosis and represent the acidosis-sensitive genes. **C** Scatter plots showing the log2FC of the genes regulated by lactic acidosis and hydrochloric acidosis. Regardless of whether only LA-induced genes (1633 DEGs) or acidosis-regulated genes (433 DEGs) were used approximately 95% of DEGs were regulated in the same sense. However, the effect of lactic acidosis was more pronounced, as indicated by the deviation of the slope of the regression line compared with the bisector. **D** Illustration of the predicted LA-regulated biological processes obtained by GO term enrichment analysis with gprofiler using Revigo. Lactic acidosis resulted in altered carbohydrate metabolism and dedifferentiation of HAoSMCs (senescent, osteo-/chondrogenic-like phenotype)

**Fig. 3:**
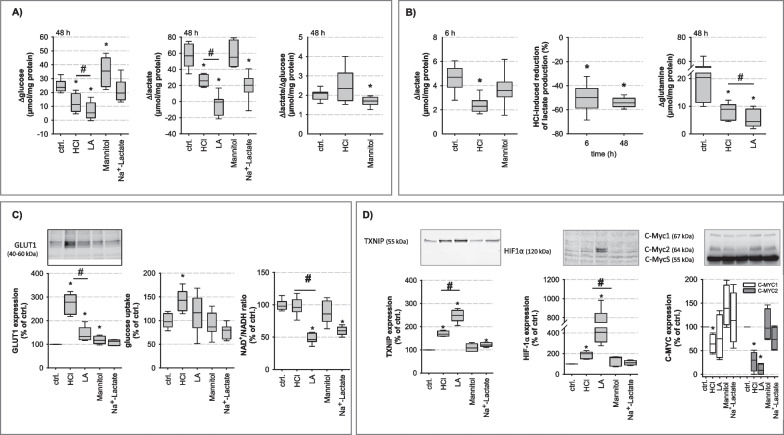
Impact of acidosis on glucose metabolism. **A** Glucose consumption and lactate production were determined after 48 h treatment (N = 4, n = 12–24; *p ≤ 3 × 10^–6^ vs. ctrl.; #p ≤ 0.04 HCl vs. LA). The respective ratio ∆lactate/∆glucose of 2 indicates a pure glycolytic metabolism. Lactic acidosis resulted in almost complete inhibition of glycolysis. **B** Lactate production was reduced by HCl treatment after 6 h and reached comparable values as after 48 h (N = 6; n = 14–18; *p ≤ 4 × 10^–8^ vs. ctrl.; #p ≤ 0.001 HCl vs. LA). Glutamine consumption was reduced after 48 h acidotic-treatment whereby lactic acidosis had the strongest effect (N = 6; n = 17–18; *p ≤ 0.0002 vs. ctrl; # p ≤ 0.002 HCl vs. LA). **C** GLUT1 protein expression was upregulated by both types of acidotic treatment (N = 5; n = 5; *p ≤ 5 × 10^–5^ vs. ctrl; # p ≤ 0.0007 HCl vs. LA) whereas the initial Glucose uptake was only increased by HCl treatment (N = 5; n = 5; *p ≤ 0.001 vs. ctrl). Furthermore, LA as well as lactate treatment led to a reduced cytosolic NAD^+^/NADH ratio in contrast to HCl treatment (N = 5; n = 15; *p ≤ 0.0001 vs. ctrl; # p ≤ 0.0001 HCl vs. LA). The loading control for each Western Blot can be found in Additional file 1: part_original Western Bot Images. **D** Protein expression of TXNIP and HIF1α showing a strong upregulation of both proteins during acidotic treatment whereby lactic acidosis has the strongest effect (N = 5; n = 4–5; TXNIP:*p ≤ 6 × 10^–5^ vs. ctrl; # p ≤ 0.001 HCl vs. LA; HIF-1α:*p ≤ 0.03 vs. ctrl; # p ≤ 0.025 HCl vs. LA). In contrast, c-MYC2 protein expression is downregulated by acidic treatment (N = 4; n = 4; *p ≤ 0.0005 vs. ctrl.). The loading controls for each Western Blot can be found in Additional file 1: part_original Western Bot Images

**Fig. 4:**
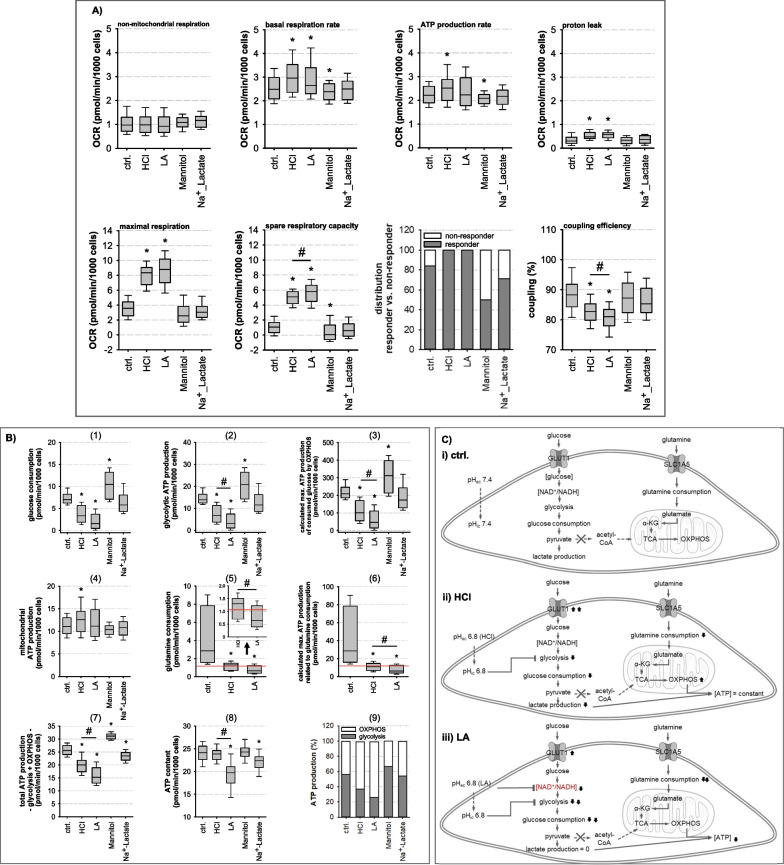
Impact of acidosis on mitochondrial function. **A** Mitochondrial functions of HAoSMCs were determined by Seahorse® technology. Basal respiration rate, proton leak, spare respiratory capacity and coupling efficiency were altered by both types of acidosis (N = 8–12; n = 44–102; *p ≤ 0.001 vs. ctrl.; # p ≤ 0.01 HCl vs.LA). **B** Comparison of glycolytic and oxidative energy metabolism. Panels 1–3 show the possible glycolytic and maximum possible mitochondrial ATP production from glucose consumption. Panel 4 presents the mitochondrial ATP production where glutamine consumption represents a possible source of energy for this purpose (panel 5–6). Panel 7 shows the calculated ATP production from glycolysis and OXPHOS whereas the cellular ATP content is depicted in panel 8. Under lactic acidosis, the cellular ATP content can no longer be maintained in contrast to HCl (panel 8). Panel 9 reveals that acidotic treated cells obtain their ATP mainly by OXPHOS and less by glycolysis. **C** Graphical summary of cellular metabolism under (i) control, (ii) pure acidosis (HCl) and (iii) lactic acidosis (LA). (I) During control conditions (pH_i_ 7.4), the ingested glucose is completely converted to lactate whereby ATP is produced. Other substrates e.g. glutamine can generate ATP in the mitochondrion via TCA and OXPHOS, allowing cells to maintain stable ATP levels. (II) During HCl, pH_i_ decreases to 6.8, resulting in lower glucose consumption, lactate production, although the NAD ^+^ /NADH ratio is not altered, and glycolytic ATP amount although more glucose is taken up. Glutamine consumption and the resulting amount of ATP decrease, but cells are still able to keep their cellular ATP content stable. (III) LA also lowers pH_i_ to 6.8, but glucose is no longer utilized and no additional lactate is produced. In addition to the inhibition of relevant glycolytic enzymes by the acidic pH, the cellular NAD^+^/NAHD ratio also decreases, which completely inhibits glycolysis and glucose is no longer consumed. In addition, glutamine utilization also decreases more than under HCl-induced acidosis. Finally, the cellular ATP decreases

**Fig. 5:**
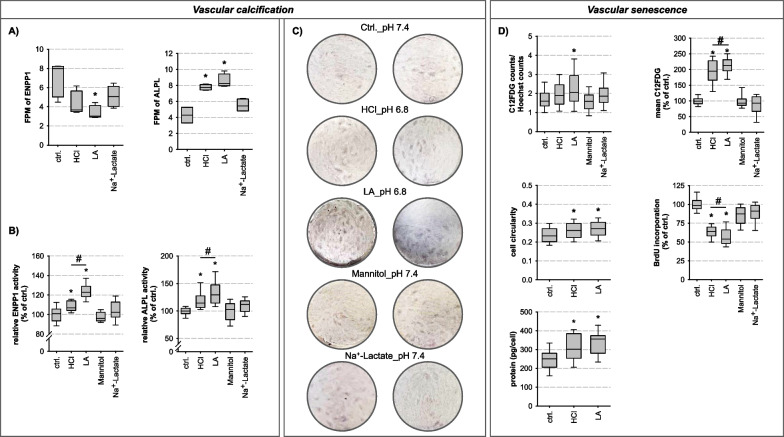
Phenotypical alterations. **A** FPMs of ENPP1 and ALPL (RNA-sequencing output). Acidosis led to downregulation of ENPP1 mRNA, whereas ALPL mRNA was increased (N = 5; n = 5). **B** ENPP1 and ALPL activity were increased during acidotic treatment with the effect being most pronounced during lactic acidosis (ENPP1: N = 3, n = 10–23; ALPL: N = 6, n = 22–24; *p ≤ 0.006 vs. ctrl.; # p ≤ 0.02 HCl vs. LA). **C** Calcium depositions stained by Alizarin Red in HAoSMCs prior exposure to β-glycerolphosphate for 14 d were shown. In particular, lactic acidosis led to an increase in calcium deposition (N = 5; n = 6). **D** Basal SA-β-gal activity was present in all cells independent of treatment conditions (C_12_FDG counts/Hoechst counts). Acidotic treatment, especially lactic acidosis, increased SA-β-gal activity (N = 6; n = 27–29; *p ≤ 4 × 10^–15^ vs. ctrl.; # p ≤ 0.03 HCl vs.LA). Cell circularity, analysed by calcein-staining, became rounder due to the acidotic treatment (0 = spindle-shaped, 1 = round). A reduced DNA synthesis rate by acidotic treatment was measured with BrdU incorporation whereby the effect was most pronounced during lactic acidosis (N = 5, n = 25; *p ≤ 2 × 10^–18^ vs. ctrl.; # p ≤ 0.03 HCl vs.LA). The protein content/cell showed a hypertrophic cell response due to acidotic treatment (N = 3–4; n = 9–21; *p ≤ 0.01 vs. ctrl.)

In the past, research on sepsis-related vascular function has mainly focused on membrane receptors, transport systems and signaling pathways in endothelial and vascular smooth muscle cells (VSMCs). Despite the strong clinical evidence for disturbed metabolic processes during sepsis, particularly the common metabolic acidosis [5], research focusing on cellular metabolism remained rare. More specifically, sepsis-associated metabolic acidosis is characterized by increasing levels of serum lactate (≥ 2 mmol/l) and is therefore a matter of lactic acidosis. Indeed, two-third of patients with sepsis or septic shock have elevated serum lactate concentrations [6]. Increase in serum lactate during sepsis is a strong indicator for an unfavorable outcome [7], indicating a possible important pathomechanistic role of lactic acidosis. Extracellular acidosis can be sensed at the cell membrane but almost certainly lead to a decrease on intracellular pH (pH_i_), thereby acting on a number of cellular processes, including signaling pathways, transcription regulation and metabolic processes [8]. Recently, we have shown that an acidic micro-environment triggers a differential transcriptional program in fibroblasts [9] and epithelial cells [10]. This induces pathologically relevant genes, e.g. inflammation-related genes, which generate a parainflammatory situation leading to loss of cell function. Systemic consequences of metabolic acidosis include vascular dysfunction, increased muscle protein catabolism, decreased albumin synthesis, renal function decline and insulin resistance [8, 11]—all consequences that also occur in septic patients.

In the present study, we used primary human aortic smooth muscle cells (HAoSMCs) to perform a comprehensive investigation of the impact of metabolic acidosis on viability, energy metabolism, mitochondrial function, gene expression and cell dedifferentiation. We focused on the differential impact of lactic acidosis- and hydrochloric-induced metabolic acidosis to define the specific role of lactic acid. Our results demonstrate a profound effect of metabolic acidosis on energy metabolism and phenotype of HAoSMCs. They also show that the impact of lactic acidosis is more pronounced compared to hydrochloric acidosis, providing a mechanistic explanation for the critical vascular dysfunction during sepsis when serum lactate concentration rise.

## Material and methods

The buffer compositions, chemical and antibodies references are provided in Additional file 1: Methods.

### Cell culture and experimental setup

Primary Human Aortic Smooth Muscle Cells (HAoSMCs; male, Caucasian; PromoCell, Heidelberg, Germany) were cultivated in Smooth Muscle Cell Growth Medium 2 (PromoCell, Heidelberg, Germany) containing 5% FCS, Epidermal Growth Factor (0.5 ng/ml), Basic Fibroblast Growth Factor (2 ng/ml) and Insulin (5 µg/ml) at 37 °C in a humidified atmosphere with 5% CO_2_. HAoSMCs at passages 2–11 were used in experiments after reaching 70–80% confluence. Cell number was determined with a Casy® cell sorter and analyzer (Innovatis, Reutlingen, Germany). 50.000 cells/cm^2^ were seeded for experiments and ~ 20.000 cells/cm^2^ for cultivating growth on 10 cm petri dishes.

Prior to the experiments, HAoSMCs were synchronized by incubation in supplement- and serum free Dulbecco's modified eagle's medium (DMEM) medium (5.5 mM glucose, 24 mM NaHCO_3_, 25 mM HEPES) for 24 h. Subsequently, cells were treated in DMEM medium for 48 h under the following conditions representing an acidotic priming: (i) control (pH 7.4); (ii) hydrochloric acid (HCl, pH 6.8); (iii) lactic acid (LA, pH 6.8, 24 mmol/l) and the respective controls (iv) mannitol (pH 7.4, 24 mmol/l) (hyperosmolar control) and (v) Na^+^-Lactate (pH 7.4, anion of lactic acid, 24 mmol/l).

### RNA sample preparation

Total RNA was isolated from HAoSMCs using BlueZol Reagent as descripted in the user manual. The RNA concentration was determined using a NanoVue™ Plus Spectrophotometer (GE Healthcare, Chicago, USA). For RNA-sequencing, eventual genomic DNA contamination was removed from the RNA samples using the TURBO DNA-free™ Kit. RNA sample were cleaned by ethanol precipitation with sodium acetate (3 M), glycogen (20 mg/ml) and 100% ethanol. To check the quality of the purified RNA samples, a 2100 Bioanalyzer System (Agilent Technologies, Karlsruhe, Germany) was used. All samples had a RNA Integrity Number (RIN) above 9.8 (with 10 as the maximal possible value).

### RNA sequencing

Novogene Co., Ltd (Cambridge, United-Kingdom) carried out the sequencing libraries preparation (poly(A) enrichment) and the paired-end sequencing (2 × 150 bp) runs on a NovaSeq6000 Illumina system (*N* = 5). Adaptor clipping and data quality control was provided by the service company as well. Read mapping to the human genome *hg38* was done with HISAT2 (v. 2.1.0) [12] and feature Counts (2.0.0, –M –t exon) [13] was used to count the mapped reads. Gene annotation was done using BiomaRt (v.2.44.4) [14] to access Ensembl archive v101. Raw RNA sequencing data and annotated counts are publicly available on Gene Expression Omnibus (GEO) database (https://www.ncbi.nlm.nih.gov/geo). GEO accession number: GSE225884.

### Differential expression analysis

Differential expression analysis was performed using edgeR (3.30.3) [15] and DESeq2 (1.28.1) [16]. Genes with sufficient counts to be considered in the statistical analyses were filtered manually for the DESeq2 analysis (genes with at least one normalized count in more than 5 samples), and using the filterByExpr edgeR function. Normalization factors were calculated with the “trimmed mean of M value” (TMM) method in the edgeR analysis. Significantly “differentially expressed genes” (DEGs) were defined as genes with a false discover rate (FDR) below 0.05 in edgeR outputs (overlap of the respective results), with at least 5 fragments per million (FPM) on average in one of the sample groups considered for a given comparison and with |log2 Fold Change|≥ 0.59.

### Gene ontology enrichment analysis

Gene Ontology (GO) term enrichment analysis was performed with the web server g:Profiler (https://biit.cs.ut.ee/gprofiler/orth) [17]. It was used to determined putatively acidosis-regulated cellular processes (using either all acidosis-sensitive DEGs (433) or all DEGs by lactic acidosis (1633)) and to check in which processes may be involved predicted upstream regulators (output of IPA analysis, see next section in Methods). Only GO terms with 5 to 3000 comprised genes were considered. GO terms were defined as significantly enriched if the adjusted *p*-value ≤ 0.001 and enrichment E ≥ 2.5, with E = (intersection size/query size) / (term size/effective domain size). Revigo (https://revigo.irb.hr) [18] was used to summarize and visualize the significantly enriched and functionally related GO terms. The LogSize scale indicates the number of the included GO terms (the larger, the more GO terms) and the color gradient corresponds to the –log(p-value).

### Ingenuity pathways analysis

Ingenuity pathway analysis (IPA) software (Qiagen, Germany) [19] was used for functional analysis, with a focus on: (i) canonical pathways (CP), (ii) upstream Regulators (UR), (iii) diseases and biofunctions (DBF) and (iv) regulatory elements networks (RE). The following for the lists of DEGs served as input: lactic acidosis DEG (DEG_LA_), hydrochloric acidosis DEG (DEG_HCl_), DEG_LA_ ∩ DEG_HCl_, DEG_LA_-NOT-DEG_HCl_. For the DBF analysis, we excluded cancer- and tumor-related categories (using *cancer*, *tumor* as search keys) to find more relevant targets concerning vascular biology. Obtained RE were filtered for consistency scores (CS). CS are defined as reliability score and a high CS value correspond to a network that is consistent with database elements. Results were filtered for |Z-score|≥ 2 and adjusted (Benjamini–Hochberg) p-value ≤ 0.01 or as it is indicated.

### Western blot

Western blotting was performed according to standard protocols. A detailed description can be found in Additional file 1: supplementary methods as well as all original blots.

### ELISA for 5-Bromo-2’-deoxyuidine (BrdU) incooperation

BrdU incorporation was measured by colorimetric indirect ELISA as described by Dubourg et al. [20]. A detailed description can be found in Additional file 1: supplementary methods.

### Senescence-induced β-galactosidase activity (SA-β-gal)

The protocol for SA-β-gal detection was adapted from Debacq-Chainiaux et al. [21]. HAoSMCs were stained with Hoechst 33342 (8.9 µM) and C_12_FDG (8.25 µM) in HEPES-Ringer buffer for 2 h at 37 °C. After washing, cells were fixed (4% formaldehyde, 30 min, RT) and rewashed. Hoechst 33342 (Excitation/Emission: 358 nm/461 nm) and C_12_FDG (Excitation/Emission: 490 nm/514 nm) were detected by digital fluorescence microscopy (Cytation3, BioTek, Bad Friedrichshall, Germany). Data analysis was performed with the Gen5 software (version 3.08, BioTek, Bad Friedrichshall, Germany).

### Caspase-3 activity assay

HAoSMCs were lysed with caspase lysis buffer (50 µl/cm^2^, 30 min, 4 °C) and centrifuged at 16,000×*g*, 5 min, 4 °C. 40 µl of cleared lysate was incubated with DEVD-AFC (40 µM) in caspase reaction buffer (90 min, 37 °C). Fluorescence of AFC was measured (excitation: 400 nm; emission: 505 nm emission) with a plate reader (Infinite M200, Tecan, Crailsheim, Germany). Cleaved AFC was quantified using a calibration curve and normalized to protein amount of the sample.

### Lactat dehydrogenase (LDH) assay

Lactate dehydrogenase (LDH) activity in media and in cell lysates was measured using standard protocols [22]. A detailed description can be found in Additional file 1: supplementary methods.

### Determination of glucose consumption and lactate production

Supernatants and lysates of HAoSMCs (MOPS-Triton lysis buffer, 50 µl/cm^2^, 30 min, 4 °C) were collected. For determination of glucose consumption: 5 µl of supernatant was incubated in glucose reaction buffer (100 µl; 15 min, 37 °C). For lactate measurement: 3–10 µl of supernatant was incubated in lactate reaction buffer (200 µl; 30 min, 37 °C). The absorbance of NADPDH/NADH was measured with a plate reader (Infinite M200, Tecan, Crailsheim, Germany). The amount of glucose/lactate in the sample was quantified by a calibration curve, subtracted from the values contained in cell culture medium and normalized to the protein amount of the sample.

### Determination of glucose uptake

The initial uptake of 2-deoxyglucose (2DG) for 10 min and the intracellular accumulation of the resulting 2-deoxyglucose-6-phosphat (2DG6P) was determined using a glucose uptake-Glo™ assay (Promega, Mannheim, Germany) according to the manufacturer’s instructions. The intracellular amount of 2DG6P was quantified by a calibration curve.

### Determination of glutamine consumption

Supernatants and lysates of HAoSMCs (RIPA buffer) were collected. Glutamine consumption was determined with the Glutamine/Glutamate-Glo™ kit (Promega, Mannheim, Germany) according to the manufacturer’s instructions. The amount of glutamine in the sample was quantified by a calibration curve, subtracted from the values contained in cell culture medium and normalized to the sample protein amount.

### Determination of oxygen consumption rate (OCR)

HAoSMCs were cultivated in 96-well Seahorse XF96 V3 PS Cell Culture Microplates (Agilent Technologies, Waldbronn, Germany). Real-time OCR was determined by Seahorse XF Cell Mito Stress Test Kit and Seahorse XFe96 Analyzer (Agilent Technologies, Waldbronn, Germany) according to the manufacturer’s instructions.

A detailed description of the measurement can be found in Additional file 1 section: supplementary methods.

### Activity measurements of vascular calcification (VC) related enzymes

Supernatants and lysates of HAoSMCs (MOPS-Triton lysis buffer, 50 µl/cm^2^, 30 min, 4 °C) were collected. The activities of VC-associated enzymes tissue-nonspecific alkaline phosphatase (ALPL) and ectonucleotide pyrophosphatase/ phosphodiesterase 1 (ENPP1) were determined by enzymatic colorimetric assays. To measure ALPL activity, 40 µl of lysate and to measure ENPP1 activity, 30 µl of lysate were incubated with 200 µl of ALPL/ENPP1 reaction buffer. The turnover of the substrates was measured: 405 nm, 1 h, 37 °C and normalized to the protein amount.

### Determination of intracellular pH (pH_i_)

Intracellular pH of single HAoSMCs was determined by the pH-sensitive dye BCECF as described by Gekle et al. [23]. A detailed description of the measurement can be found in Additional file 1: supplementary methods.

### Determination of matrix calcification

After 48 h acidic priming, HAoSMCs obtained a calcifying DMEM medium (5% FCS, β-glycerophosphate (10 mM), ascorbic acid (25 µg/ml) and CaCl_2_ (0.5 mM)) for 14 d. Calcium depositions were visualized by alizarin red staining. Therefore, cells were washed, fixed (4% formaldehyde, 30 min, RT), rewashed and stained with Alizarin red (58 mM, 45 min). Then, the cells were washed with water and photographed.

### Determination of alterations in cell morphology

HAoSMCs were stained with Calcein-AM (2 µM) and Hoechst 33342 (6 µM) in a HEPES-Ringer solution (37 °C, 30 min). After washing, Hoechst 33342 (Excitation/Emission: 358 nm / 461 nm) and calcein (494 nm / 517 nm) signals were detected by digital fluorescence microscopy (Cytation3, BioTek, Bad Friedrichshall, Germany). Calculation of cell circularity was performed with the Gen5 software (version 3.08, BioTek, Bad Friedrichshall, Germany).

### Determination of total cellular ATP content

Quantitative ATP measurement was assessed by the ATP Bioluminescence Assay Kit HS II (Roche, Berlin, Germany) according to the manufacturer’s instructions. The amount of intracellular ATP was quantified using a calibration curve.

### Determination of the NAD^+^/NADH ratio

The cellular NAD^+^/NADH ratio was determined by the NAD/NADH-Glo™ assay (Promega, Mannheim, Germany) according to the manufacturer’s instructions.

### Statistical analysis

For all laboratory confirmation experiments, significant differences in between groups was assessed by Wilcoxon Rank test or ANOVA on Rank with correction for multiple testing (*p* < 0.05). *Chi-*squared test for outlier removal was performed with outliers R package (https://cran.r-project.org/package=outliers). N represents the number of individual experiments and n the number of wells or culture dishes investigated per experiment.

## Results

In order to unveil the specific impact of sepsis-related lactic acidosis on HAoSMCs, we compared the effect of lactic acidosis (metabolic acidosis + hyperlactataemia = LA_pH 6.8 furtherly abbreviated as LA) to the pure metabolic acidotic effect (hydrochloric acidosis_pH 6.8 = furtherly abbreviated as HCl) and hyperlactataemia at pH 7.4 (Na^+^-lactate) as well as to the respective controls with pH 7.4 (ctrl.; mannitol (hyperosmolarity control)).

### Effect of acidosis on viability and intracellular pH (pH_i_) of HAoSMC

Figure 1A–C show the acute (10 min, Fig. 1A, B) and chronic (48 h; Fig. 1C) effect of extracellular acidosis (pH 6.8) on pH_i_. Under control conditions (pH 7.4), pH_i_ was slightly higher (pH 7.53 ± 0.02) compared to the extracellular compartment (pH_e_: 7.4). Both types of acidosis led to a rapid and sustained intracellular acidification of similar magnitude. The change of pH_i_ rapidly follows the extracellular acidosis without a significant counter-regulation of the cell in response to the acidification.

In order to evaluate the influence of acidosis on cell viability, protein content, cell number, necrosis (LDH release) and apoptosis (caspase-3 activity) were analyzed. Figure 1D shows that acidosis did not reduce cellular protein content. On the contrary, the protein amount was increased by about 20–35% due to acidotic treatment. Cell number/cm^2^ was unaffected by acidosis (HCl: 98 ± 3% of ctrl., LA: 99 ± 3% of ctrl., N = 6, n = 48–84). Calculation of the relative protein amount/cell (hypertrophy marker) revealed that both forms of acidosis led to cell hypertrophy (Fig. 5D).

The reduction of pH_e_ by either HCl or LA did not enhance cellular LDH release after 48 h (Fig. 1E, F). Instead, both types of acidosis reduced cell necrosis at pH 7.1 and pH 6.8, as did mannitol treatment. Na^+^-Lactate, but not equimolar mannitol, induced a slight increase in LDH release (Fig. 1E). Caspase-3-activity was not enhanced substantially by acidosis (Fig. 1F) and only to *a minor extent* by Na^+^-lactate (Fig. 1F). The values for 24 and 72 h incubation are shown in Additional file 1: Figs. S1 and S2. We observed a trend to enhanced cell death and reduced cellular protein abundance at pH 6.4 after 72 h. Thus, for all further experiments experimental acidosis of pH 6.8 for 48 h was applied. These data show, that our experimental acid–base status conditions, which are compatible with a clinical situation during severe sepsis, did not induce a generalized, non-specific cell damage but inhibit necrotic and apoptotic cell death with concomitant development of cell hypertrophy (Fig. 5D).

### Acidosis-induced alterations of the HAoSMC transcriptome

RNA-sequencing of HAoSMCs samples showed that a much larger number of genes were differentially expressed under lactic acidosis (1633 DEGs) than under pure hydrochloric acidosis (492 DEGs), whereas hardly any genes were affected under hyperlactacemia (55 DEGs) (Fig. 2A). The principal component analysis (PCA) also showed that the variation between the sample groups is probably mostly due to the different pH values (PC1) (Fig. 2A). In addition, it reveals that the type of acidosis is of essential importance. These data already show that lactic acidosis influences VSMC to a greater extent than hydrochloric acidosis, despite similar changes in pH_i_ (Fig. 1C). Analysis of the genes affected by the different conditions (Fig. 2B) showed that about 95% of the DEGs affected by hydrochloric acidosis are also and concordantly affected by lactic acidosis (Fig. 2C) and can be considered therefore as acidosis-sensitive. Similarly, 85% of DEGs affected by lactic acidosis showed concordant changes during hydrochloric acidosis, with 75% of cases failing to reach the level of significance in HCl-induced acidosis (Fig. 2C). Thus, acidosis and lactate act synergistically with respect to transcriptome changes. GO-term enrichment analysis of the lactic acidosis-sensitive genes (Fig. 2D) predicted major changes in carbohydrate and energy metabolism, as well as phenotypic dedifferentiation (osteogenic, senescent phenotype, cell size and shape alterations) of VSMC. GO term enrichment analysis of the acidosis-sensitive genes is shown in Additional file 1: Fig. S3A.

Further functional analysis was performed with IPA®. Canonical pathway (CP) analysis for the set of DEG_HCl_ and DEG_LA_ revealed only few pathways with low adjusted p-values and Z-scores (see Additional file 1: Fig. S3B). The analysis of the DEG regulated by lactic acidosis but not by hydrocholoric acidosis (DEG_LA_-NOT-DEG_HCl_) resulted in one CP associated with mitochondrial function (Z-score = − 1.75; see Additional file 1: Fig. S3C).

Upstream regulator (UR) analysis for DEG_LA_ and DEG_HCl_ resulted in 104 predicted UR with in at least one of the two gene sets (adjusted p-values: < 0.01, |Z-scores|> 2, Additional file 1: Table S1). 15 UR were not concordant. 41 UR shown |Z-scores|> 1 in both gene sets. These data show that acidosis per se exerts a strong transcriptional effect that is augmented synergistically by lactate, because lactate per se exerted no effect.

Then, we used the two sets of predicted UR for a g:Profler multiquery to compare the putative GO term enrichment (Additional file 1: Table S2). Analysis of the set of genes regulated by both types of acidosis (DEG_LA_ ∩ DEG_HCl_, data not shown separately) yielded the same results as the one of the set DEG_HCl_, which was not surprising given the 95% overlap of DEG_LA_ and DEG_HCl_ (Fig. 2C). Analysis results of the DEG_LA_-NOT-DEG_HCl_ set, which represent the lactic acidosis-specific effect (i.e. the synergism of lactate and acidosis), as well as the GO term enrichment analysis of the UR results, are listed in Additional file 1: Tables S4 and S5 showing a trend towards ossification and MAPK signaling.

Disease and biofunction (DBF) analysis resulted in 43 functions predicted for at least one of the two sets DEG_LA_ or DEG_HCl_. The number of DBF with |Z-score|> 2 was larger for the DEG_LA_ set. Excluding cancer related terms resulted in 21 DBF, including 7 related to vasculature, 6 to cell motility and 3 to energy substrate metabolism (in Additional file 1: Table S3). Finally, regulatory elements networks (RE) were predicted by IPA, an additional feature, which has the advantage to link hereinabove mentioned predictions and therefore allows the formulation of advances hypothesis. Shortly, this networking makes literature- and evidence-based links between predicted upstream regulators, regulated genes (served as input for the analysis) and predicted regulated functions. Obtained RE were filtered for consistency scores (CS) > 10 (see Additional file 1). No RE were predicted for DEG_HCl_ whereas three RE were predicted for DEG_LA_ (see Additional file 1: Fig. S3D). This bioinformatic analysis did not predict UR or DBF in the opposite direction (activation vs. inhibition) for the two types of acidosis. Rather, the predicted values for UR/DBF are stronger for lactic acidosis compared to acidosis alone, indicating a synergistic effect related to disturbed energy metabolism and dedifferentiation.

### Acidosis-induced alterations of HAoSMC glucose and glutamine consumption

In order to validate the bioinformatics predictions, we analysed VSMC glucose and energy metabolism. Acidosis reduced glucose consumption (Δ glucose) and lactate production (Δ lactate) during 48 h significantly (Fig. 3A), without affecting the molar ratio Δlactate/Δglucose of 2. The inhibitory effect of acidosis on carbohydrate metabolism could be observed already after 6 h (Fig. 3B). Due to the high background level of lactate during lactic acidosis, reliable determination of Δlactate could be performed only for hydrochloric acidosis. Of note, the inhibitory effect of lactic acidosis on glucose consumption was significantly larger as compared to hydrochloric acidosis (Fig. 3A). These data show that an equivalent to the glucose consumed is converted into lactate under control and hydrochloric acidosis, whereas there is virtually no measurable glycolytic digestion of glucose during lactic acidosis.

Reduced glucose metabolism was not the result of impaired glucose uptake, because GLUT1 expression and the initial 2DG6P uptake rate (a measure for glucose transport activity) were not reduced by acidosis (Fig. 3C). But we observed a reduced NAD^+^/NADH ratio by LA and lactate treatment (Fig. 3C). The measured cellular NAD^+^ and NADH concentrations, which contribute to the calculated NAD^+^/NADH ratio, are shown in Additional file 1: Fig. S4. Due to the high lactate concentrations, it is likely that the GAPDH-catalysed glycolytic reaction was inhibited [24], which completely prevented glycose consumption. In contrast, HCl treatment did not reduce the NAD^+/^NADH ratio, allowing glycolysis to continue, albeit reduced due to intracellular acidification.

Interestingly, GLUT1 expression and 2DG6P uptake were enhanced by hydrochloric acidosis, to a much larger extent as compared to lactic acidosis. This difference may result from the upregulation of TXNIP (Fig. 3D; Additional file 1: Fig. S5), an inhibitor of GLUT1-expression and glycolysis [25], which is more upregulated under lactic acidosis than in hydrochloric acidosis. The transcription factor c-MYC is known to reduce TXNIP protein expression, thereby increasing the glycolytic rate [26]. Western blot analyses showed a strongly reduced c-MYC2 expression, particularly during lactic acidosis (Fig. 3D; Additional file 1: Fig. S5), which may explain increased TXNIP expression during lactic acidosis.

These data confirm the bioinformatics prediction regarding impaired carbohydrate metabolism and suggest that lactic acidosis leads to an inhibition of glucose metabolism and thus more severe impairment of energy homeostasis. The stronger upregulation of HIF-1α, an indicator of energy shortage [27], during lactic acidosis compared to hydrochloric acidosis (Fig. 3D; Additional file 1: Fig. S5) supports this conclusion. Besides glucose, glutamine is another possible source of energy and lactate. Similar to glucose, acidosis reduced glutamine consumption and the inhibitory effect of lactic acidosis was significantly stronger as compared to hydrochloric acidosis (Fig. 3B).

### Acidosis-induced alterations of HAoSMC energy homeostasis

Figure 4A shows the mitochondrial function of HAoSMCs after 48 h treatment, assessed as the mitochondrial oxygen consumptions rate (OCR) with the Seahorse® technology. We observed a slight but significant acidosis-induced increase in basal mitochondrial respiration and in the mitochondrial proton leakage. Coupling efficiency was also reduced slightly. Furthermore, we observed a substantial acidosis-induced increase in maximal mitochondrial respiration and thus in spare respiratory capacity. Figure 4B shows the quantitative comparison of maximum possible glycolytic ATP production (panel 2), calculated maximal ATP production capacity from glucose (= glycolysis + OXPHOS, panel 3), mitochondrial ATP production (according to the OCR, panel 4) and measured cellular ATP content (panel 8). To make OCR measurements comparable with glucose consumption, we converted the results obtained for glucose consumption during 48 h (Fig. 3A) to pmol/min/1000 cells (panel 1 in Fig. 4B). Glycolytic ATP production was calculated assuming that 2 mol ATP are formed when 1 mol glucose is consumed. As shown in panel 2 of Fig. 4B, acidosis led to a reduction in glycolytic ATP production, with lactic acidosis having a significant stronger effect than hydrochloric acidosis. From the glucose consumed, the maximum possible amount of ATP produced by mitochondria was calculated (30 mol ATP per mol glucose consumed; panel 3 in Fig. 4B). Panel 4 in Fig. 4B shows that VSMCs use only a minor fraction of glucose for OXPHOS-derived ATP production according to the ATP-synthesis dependent OCR. Besides glucose, another substrate that can serve for mitochondrial ATP production or lactate formation is glutamine. The determined glutamine consumption (Fig. 3B) was also normalized to pmol/min/1000 cells (panel 5 in Fig. 4B). Mitochondria produce 10 mol ATP from 1 mol of glutamine using 2 mol of O_2_. Panel 6 in Fig. 4B shows the maximum mitochondrial ATP production from the glutamine consumed. The additional red lines in panel 5 and 6 of Fig. 4B indicate the amount of glutamine required to fuel mitochondrial ATP production derived from OCR (panel 4 in Fig. 4B). Under control conditions, glutamine is more than sufficient to fuel mitochondrial ATP production in contrast to acidotic conditions. The lower glutamine consumption under hydrochloric acidosis is still sufficient for mitochondrial ATP production. However, during lactic acidosis this is no longer the case (marked by the red line in panel 5 in Fig. 4B)

According to the calculated ATP production capacities (assuming that lactate is derived from glucose), lactic acidosis leads to a more pronounced shortage of energy supply (ATP production from glycolysis + ATP production from OXPHOS) than hydrochloric acidosis (panel 7 in Fig. 4B). To test this prediction we measured the cellular ATP content after 48 h exposure to acidosis. As shown in panel 8 of Fig. 4B, cellular ATP content was reduced significantly during lactic acidosis but not during hydrochloric acidosis. Thus, lactic acidosis leads to a critical shortage of ATP-regeneration that cannot be compensated, resulting in reduced ATP availability, which represents a severe threat and impairs cellular function. In the case of hydrochloric acidosis, VSMC are able to maintain the ATP level stable. This is most probably the result of a less pronounced reduction in glucose and glutamine consumption (Fig. 4B) and a restriction of ATP-consuming processes (not determined in this study). Furthermore, under acidosis, the relative contribution of glycolysis and OXPHOS to ATP production changed. Under control conditions, ATP was produced by both glycolysis and OXPHOS in equal proportions, whereas under acidic conditions the relative contribution of OXPHOS increased significantly (panel 9 in Fig. 4B). The results for the three conditions are graphically summarized in Fig. 4C.

### Acidosis-induced transdifferentiation of HAoSMC from the contractile to a senescent, osteo-/chondrogenic phenotype

Figure 5A, Additional file 1: Figs. S6 and S7 show the acidosis-induced changes in mRNA abundance of biomarkers for vascular calcification (VC). With the exception of ENPP1, all changes indicate enhanced calcification activity under acidic conditions, with a stronger impact of lactic acidosis as compared to hydrochloric acidosis.

To investigate whether these changes in mRNA abundance translate in enhanced calcification activity in our experimental system, we determined the activity of two crucial enzymes for VC, ENPP1 and ALPL. Figure 5B shows that acidosis leads to an upregulation of the activity of both enzymes. The impact of lactic acidosis was stronger as compared to hydrochloric acidosis. Furthermore, we determined the deposition of extracellular calcium complexes and observed enhanced calcification during lactic acidosis and to a smaller extent also during hydrochloric acidosis (Fig. 5C).

It is notable that ENPP1 mRNA expression and ENPP1 enzyme activity are inversely regulated (Fig. 5A, B). This may be the result of a negative feedback loop where increased ENPP1 activity leads to decreased ENPP1 mRNA expression.

Villa-bellosta et al. also reported an inverse correlation between ENPP1 mRNA expression and activity. In rats injected with calcitriol, leading to media calcification after 3 days, ENPP1 mRNA levels were increased while ENPP1 enzymatic activity was decreased [28]. In addition, post-translational modifications of ENPP1 can lead to an increased ENPP1 activity. Stefan et al. showed that autophosphorylation of threonine 256 resulted in inhibition of ENPP1 activity at low ATP concentrations. At higher cellular ATP concentrations, as was the case in our cells, ENPP1 was dephosphorylated, resulting in ATP hydrolysis and PP_i_ formation [29].

**Fig. 6:**
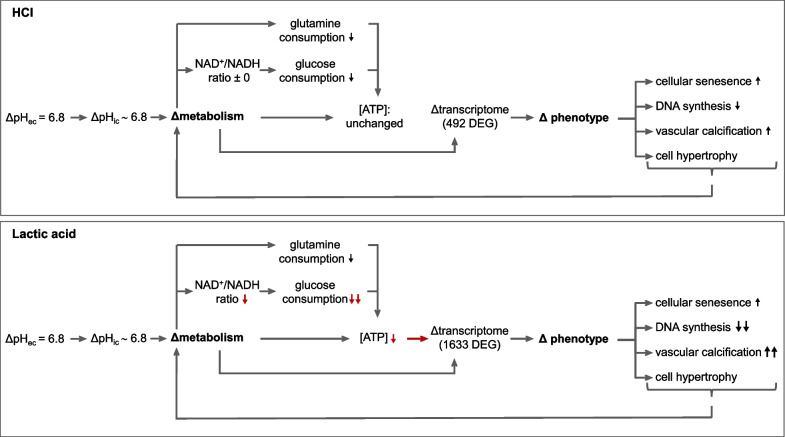
Graphical summary: impact of hydrochloric- and lactic acidosis on HAoSMCs. Extracellular acidification (pH 6.8) resulted in intracellular acidification by both forms of acidosis, without counter-regulation of the cells, leading to a pH_i_ of 6.8. Hydrochloric and lactic acidosis treatment led to an altered cell metabolism with reduced glucose and glutamine consumption. However, the effect is much more pronounced in lactic acidosis than in hydrochloric acidosis due to a reduced NAD^+^ /NADH ratio leading to inhibition of glycolysis. In hydrochloric acidosis, the cells manage to keep the ATP content constant, whereas this is no longer possible in lactic acidosis. This results in a substantially larger number of DEG for lactic acidosis (1633 DEG) compared to hydrochloric acidosis (492 DEG). Cells respond to metabolic-induced transcriptome changes with phenotypic changes (e.g. cellular senescence, vascular calcification) that are again more pronounced in lactic acidosis

Finally, we determined VSMC senescence by measuring two typical markers: senescence-associated β-galactosidase activity and BrdU-incorporation into DNA (Fig. 5D). Acidosis enhanced senescence-associated β-galactosidase activity and reduced BrdU incorporation. Furthermore, we observed a slight change in cell morphology/cell shape, which we quantified by determining cellular circularity (Fig. 5D). During acidosis circularity increased, indicating a less spindle-shaped phenotype. Furthermore, we found an increase of protein/ cell indicating an induction of cellular hypertrophy due to acidotic treatment (Fig. 5D). These data show that phenotypic changes in VSMCs occur during acidosis, typically leading to senescent, osteo-/chondrogenic, and hypertrophic VSMCs that may occur during sepsis (Fig. 5A-D). Figure 6 illustrates the phenotypic changes in relation to the metabolic alterations. Despite equal acidification, LA treatment leads to a complete inhibition of glycolysis due to a reduced NAD^+^/NADH ratio, which reduces the cellular ATP content. This induces significantly stronger transcriptional and phenotypic changes in the HAoSMCs than pure acidosis treatment (Fig. 6).

## Discussion

During sepsis the vascular system is critically affected resulting in vasoplegia, hypotension and organ hypoperfusion, which often determines the clinical outcome [1, 3]. However, the underlying molecular mechanisms involved are incompletely understood, making rational intervention approaches limited. The septic patient has an altered metabolism leading to lactic acidosis that is associated with high mortality and poor prognosis [30, 31]. Various causes leading to lactic acidosis during sepsis are discussed: (i) accelerated aerobic glycolysis from adrenergic stress (lung, skeletal muscle, liver and leukocytes), (ii) impaired lactate clearance, (iii) disorders of microcirculation, (iv) mitochondrial impairments, (v) tissue malperfusion (hypoxia) and (vi) elevated ADP/ATP ratio [7, 32]. To date, it is not clear whether lactate per se, pure acidosis, or the combination of the both—lactic acidosis—causes, at least in part, the deleterious effects of sepsis. We investigated the influence of sepsis-induced lactic acidosis on transcriptome, metabolism and phenotype of primary human VSMCs in comparison to plain acidosis and hyperlactatemia.

Extracellular acidification by lact- or hydrochloric acidosis resulted in the same intracellular acidification without major cellular counter-regulation and did not lead to an impairment of cell viability. This corresponds well to the situation in septic patients since the cell death in the affected organs is only slightly pronounced [33].

### Transcriptome alterations

Sole hyperlactatemia resulted in almost no DEGs, whereas hydrochloric acidosis resulted in 492 DEGs. A combination of lactate and acidosis exerted a synergistic (= overadditive) effect and resulted in 1633 DEGs. Thus, lactate needs an acidic pH in order to become effective with respect to transcriptome alterations [34]. Comparison of DEG_HCl_ with DEG_LA_ revealed that ~ 95% of the genes were regulated concordantly, with a stronger effect size in lactic acidosis (= quantitative effect). A similar result is seen in the analysis of patients who survived sepsis compared to non-survivors, which exhibit similar metabolic and proteomic patterns but with a greater magnitude of changes [35]. The changes of the transcriptome by lactic-/ acidosis were predicted to lead to alterations of cellular metabolism and phenotype by GO-term enrichment and pathway analysis, whereby the changes are expected to be more pronounced during lactic acidosis.

### Metabolic alteration

Acidosis-induced suppression of glucose consumption and lactate production was not the result of impaired glucose uptake because neither 2DG uptake nor GLUT1 protein expression were decreased. On the contrary, acidosis even increased GLUT1 protein expression and glucose uptake activity in attempt to provide more energy sources. It may be partly explained by the acidosis-induced HIF1α protein abundance, a transcription factor known to increase GLUT1 expression [27]. Although lactic acidosis causes stronger HIF1α protein expression compared to hydrochloric acidosis, it does not lead to increased glucose uptake due to the strong upregulation of TXNIP, a very potent suppressor of GLUT1 and a HIF1α destabilizing protein [25, 36]. Thus, more energy source in form of glucose is provided during hydrochloric acidosis but not during lactic acidosis, potentially leading to a critical shift in energy homoeostasis in the latter case.

Inhibition of the glycolytic regulatory enzyme phosphofructokinase 1 (PFK-1) by acidosis is a very well know phenomenon [37] that can be assumed to occur also under our experimental conditions. This is supported by the rapid inhibition of lactate production already after 6 h. We propose that a PFK-1-induced reduction of glucose consumption leads to a compensatory increase in HIF1α abundance, which elevates the expression of glucose transporters as well as glycolytic enzymes [27, 38], trying to counteract the reduced glucose metabolism. However, during lactic acidosis, the parallel upregulation of TXNIP counteracts this mechanism and glucose consumption is reduced to a larger extent compared to hydrochloric acidosis. The function of TXNIP to prevent glucose uptake by downregulating GLUT1 and the expression of key glycolytic enzymes through reduction of HIF1α expression has attracted increasing attention over the last years [25]. A further explanation for inhibited glucose consumption during lactic acidosis is the lactate-induced inhibition of the glycolytic glyceraldehyde-3-phosphate dehydrogenase (GAPDH) forward reaction due to elevated NADH concentrations [24]. Hereby, intracellular lactate is converted by LDH into pyruvate [39] accompanied by an increase in NADH concentrations. An important prerequisite for extracellular lactate-induced inhibition of glycolysis is its uptake by H^+^-monocarboxylate cotransporters (MCTs) [40]. In the case of Na^+^-Lactate exposure at pH 7.4, lactate uptake is low and therefore no decrease in glucose consumption was observed. During lactic acidosis (pH 6.8), the higher proton availability and the pH dependence of MTCs lead to a significantly higher lactate uptake activity, resulting in a stronger decline in glycolysis during lactic acidosis compared to hydrochloric acidosis as a result of a reduced NAD^+^/NADH ratio followed by GAPDH inhibition. Further support for our hypothesis of a disturbed NAD^+^/NADH ratios under lactic acidosis comes from altered mRNA expression of NAD^+^ consuming enzymes, such as phosphoglycerate dehydrogenase (PHGDH) and sirtuin-1 (SIRT-1; Additional file 1: Fig. S8).

The reason for the limited increase in OXPHOS during lactic acidosis despite increased NADH levels needs to be investigated in further studies. One possible explanation is insufficient mitochondrial import of cytosolic NADH across NADH-impermeable membrane by specialized shuttle systems (malate-aspartate shuttle; glycerol-3 phosphate shuttle). As transporters and enzymes of the malate aspartate shuttle (SLC25A12, SLC25A13, GOT1) seem to be downregulated (Additional file 1: Fig. S9) a reduced NADH import is conceivable. Another possibility is that there is a compartmentalisation of non-oxidative and mitochondria-associated oxidative glycolytic pathways as described by Barron et al. as well as by Fuller et al. [41, 42]. Therefore, an increase of the cytosolic NADH must not necessarily result in increased ATP generation by OXPHOS. In T cells it has been shown that the reductive stress can lead to a depletion of post GAPDH intermediates, which could prevent the compensatory OXPHOS increase [24]. Additionally, NAD^+^ regeneration can be disturbed, e.g. by alterations of the mitochondrial membrane potential that affect by complex I. For septic patients, it is described that inhibition of tricarboxylic acid cycle (TCA) and OXPHOS occurs and an increase of mitochondrial activity by dichloroacetate improves their outcome [43, 44]. In our study, no direct inhibition of mitochondrial activity by both types of acidosis was observed. The spare respiratory capacity was even higher. Furthermore, only a small increase of the proton leak was detectable. Thus, if mitochondria are inhibited during sepsis this seems not to be the results of acidosis. As the molar glucose consumption corresponds to twofold the molar lactate production, additional substrates are required for the complete energy balance, including mitochondrial ATP production. A relevant additional source is glutamine, whose consumption is also reduced by acidosis, with more pronounced effect of lactic acidosis. Thus, there is not only a critical reduction of glucose consumption by lactic acidosis but also of the second relevant energy source, glutamine.

It is conceivable that the transcription factor c-MYC plays a role not only for altered glucose consumption but also during the reduced glutaminolysis. C-MYC has been shown to promote glutaminolysis by inducing glutaminase1 (GLS1) expression and to increase glycolytic rate by inhibiting TXNIP expression [26]. Thereby, three different isoforms of c-MYC exist due to alternative initiation of translation: c-MYC1, c-MYC2 and C-MYCS [45]. Western blot analyses revealed a down-regulation of C-MYC2. Decreased C-MYC2 expression reduced its inhibitory effect on TXNIP expression, which in turn inhibits the glycolytic rate. In addition, during lactic acidosis reduced c-MYC2 expression also slows down glutaminolysis.

Finally, during hydrochloric acidosis VSMCs are able to maintain the cellular ATP content despite reduced utilization of energy substrates. By contrast, VSMCs are no longer able to maintain ATP homeostasis during lactic acidosis, resulting in a condition of disturbed function and structure. We propose that this critical metabolic decompensation during lactic acidosis is responsible for the much more extensive transcriptional changes that lead to phenotypic changes of VSMCs (Fig. 6).

### Phenotypic alterations

Under pathophysical stress conditions, VSMCs change their phenotype, switching from the contractile phenotype to (i) a dedifferentiated secretory, or (ii) senescent, or (iii) migrating /proliferating, or (iv) osteo-/chondrogenic phenotype [3, 46]. We observed a dedifferentiation to a mixed senescent, osteo-/chondrogenic phenotype. Dedifferentiation to the osteo-/chondrogenic phenotype is mainly due to the enhanced expression of calcifying genes (producing phosphate (P_i_)) and a reduced expression of anti-calcifing enzymes (producing pyrophosphate (PP_i_)) [47] which finally generates extracellular hyperphosphatemia [48] that leads to the shown VC [49]. Important proteins regulating the P_i_/PP_i_ ratio and affected by acidosis include: ENPP1, ALPL, NT5E (5ʹ-nucleotidase ecto), SLC20A1/2 (solute carrier family 20 member 1/2) and SLC17A9 (solute carrier family 17 member 9) [47]. SLC17A9, a vesicular ATP transporter [50] can lead to increased extracellular ATP levels (ATP_e_)—the substrate for ENPP1. ENPP1 hydrolyses ATP_e_ to AMP and PP_i_. Most PP_i_ is degraded to P_i_ by ALPL [28]. ENPP1 as well as ALPL activity are increased during acidosis whereby lactic acidosis exerts the strongest effect. The enhanced activity of ENPP1 and ALPL led to an elevated P_i_ /PP_i_ ratio promoting Ca^2+^-phosphate deposition. It has been shown that ALPL overexpression in VSMCs is sufficient for ex vivo calcification of aortic rings [28]. The increase in extracellular P_i_ is furthered by reduced expression of SLC20A1/2, which normally clears P_i_ from the extracellular space [47]. Moreover, acidosis-induced decreased NT5E expression (converts AMP to adenosine + P_i_) results in decreased adenosine production, thus lacking another anti-calcifying substance. Normally, adenosine counteracts VC in HAoSMCs by reducing ALPL expression and activity [51, 52]. Finally, elevated extracellular P_i_ promotes the expression of osteo-/chondrogenic transcription factors like SRY-box transcription factor 9 (SOX9) and runt-related transcription factor 2 (RUNX2) in VSMCs what was also detectable after lactic acidotic treatment. Increased RUNX2 and SOX9 expression likewise increase ALPL activity [48] leading to a vicious circle. The transdifferentiation process is accompanied by the induction of cellular senescence, reduced DNA synthesis/DNA repair capacity and cell hypertrophy. Because glycolysis and glutaminolysis are essential to fuel the biosynthesis of nucleotides, amino acids and lipids to support cell viability and growth [26], the reduced glycolytic flux and glutaminolysis may also be responsible for transdifferentiation. Additionally, a reduced NAD^+^/NADH ratio leads to a reduced activity of PHGDH, a NAD^+^-dependent enzyme, which catalyzes the first step of the serine biosynthesis pathway and is a key contributor to purine synthesis [24]. The stronger effect of lactic acidosis on the metabolic pathways is in line with its stronger effect on DNA synthesis. This VSMC transdifferentiation may provide an explanation why sepsis survivors have a 3.3-fold increased risk of suffering from limitations in daily living after recovery compared to the normal population [3, 53]. VSMC transdifferentiation seems to persist even after surviving sepsis and thus may have a long-term effect on the VSMCs and vessels.

## Conclusion

Overall, our study investigate for the first time in a structured and comprehensive manner the influence of lactic acidosis on primary human VSMCs and contrasts it with plain acidosis. We clearly showed that lactate alone cannot cause sepsis-induced VSMC changes but that a synergistic combination of acidosis and lactate (lactic acidosis) is required.

Lactic acidosis induces numerous metabolic and phenotypic changes in VSMC. The high lactate concentrations lead to a complete inhibition of the glycolysis due to a reduced NAD^+^/NADH ratio. The additionally decreased glutaminolysis results in a reduced cellular ATP content which may explain the acute vascular dysfunction. The metabolic alterations lead to transcriptome dysregulation and finally to senescent, osteo-/chondrogenic transdifferentiation. These transcriptome, proteome and phenotype changes in VSMCs result in vascular remodelling and can explain the post-sepsis suffering of patients after surviving the acute phase of sepsis.

### Supplementary Information

**Additional file 1: Figure S1.** Cell viability after 24 h. **Figure S2.** Cell viability after 72 h. **Figure S3.** Acidosis-induced changes of the HAoSMC transcriptome. **Figure S4.** Determination of the cellular NAD+ and NADH concentration. **Figure S5.** Expression changes of genes involved in metabolic regulation. **Figure S6.** Expression changes of additional calcification-associated genes. **Figure S7.** Expression changes of osteochondrogenic genes. **Figure S8.** Expression changes of NAD+-consuming enzymes. **Figure S9.** Expression changes of genes involved in the malate-aspartate shuttle. **Table S1.** upstream regulator (UR) for DEGLA and DEGHCl which were identified by IPA. **Table S2.** g:Profler multiquery to compare the putative GO term enrichment of the two sets of predicted UR. **Table S3.** Disease and biofunction (DBF) analysis which were generated by IPA. **Table S4.** DEGLA-NOT-DEGHCl set (lactacidosis-specific effect) by IPA. **Table S5.** GO term enrichment analysis of the UR results.

## Data Availability

The data underlying this article are available in the manuscript and in its online additional material. Raw RNA sequencing data and annotated counts are publicly available on Gene Expression Omnibus (GEO) database (https://www.ncbi.nlm.nih.gov/geo). GEO accession number: GSE225884.

## Abbreviations

ALPL: Tissue-nonspecific alkaline phosphatase
AFC: 7-Amino-4-trifluoromethyl coumarin
BCECF: 2′,7′-Bis-(2-carboxyethyl)-5-(and-6)-carboxyfluorescein
BrdU: 5-Bromo-2'-deoxyuridine
C_12_FDG: 5-Dodecanoylaminofluorescein di-β-d-galactopyranosid
DEGs: Differentially expressed genes
2DG: 2-Desoxyglucose
2DG6P: 2-Desoxyglucose-6-phosphate
DMEM: Dulbecco's modified eagle's medium
ENPP1: Ectonucleotide pyrophosphatease/phosphodiesterase 1
FCCP: P-trifluoromethoxy carbonyl cyanide phenylhydrazone
GLS1: Glutaminase 1
G6P: Glucose-6 phosphate
G6PD: Glucose 6 phosphate dehydrogenase
HAoSMCs: Human aortic smooth muscle cells
HEPES: 4-(2-Hydroxyethyl)-1-piperazineethanesulfonic acid
IPA: Ingenuity pathway analysis
LDH: Lactate dehydrogenase
c-MYC: MYC proto-oncogene
NAD^+^/NADH: Nicotinamide adenine dinucleotide
NT5E: 5'-Nucleotidase ecto
OCR: Oxygen consumption rate
pHe: Extracellular pH
PHGDH: Phosphoglycerate dehydrogenase
pHi: Intracellular pH
RIPA: Radioimmunoprecipitation assay buffer
RUNX2: Runt-related transcription factor 2
SIRT1: Sirtuin 1
SLC17A9: Solute carrier family 17 member 9
SLC20A1: Solute carrier family 20 member 1
SLC20A2: Solute carrier family 20 member 2
SOX9: SRY-box transcription factor 9
TGM2: Transglutaminase 2
VC: Vascular calcification
VSMCs: Vascular smooth muscle cells

## References

[1] Arina P, Singer M (2021). Pathophysiology of sepsis. Curr Opin Anaesthesiol.

[2] Sun J, Zhang J, Tian J, Virzi GM, Digvijay K, Cueto L, Yin Y, Rosner MH, Ronco C (2019). Mitochondria in sepsis-induced AKI. J Am Soc Nephrol.

[3] Strela FB, Brun BF, Berger RCM, Melo S, de Oliveira EM, Barauna VG, Vassallo PF (2020). Lipopolysaccharide exposure modulates the contractile and migratory phenotypes of vascular smooth muscle cells. Life Sci.

[4] Burgdorff AM, Bucher M, Schumann J (2018). Vasoplegia in patients with sepsis and septic shock: pathways and mechanisms. J Int Med Res.

[5] Kraut JA, Madias NE (2010). Metabolic acidosis: pathophysiology, diagnosis and management. Nat Rev Nephrol.

[6] Casserly B, Phillips GS, Schorr C, Dellinger RP, Townsend SR, Osborn TM, Reinhart K, Selvakumar N, Levy MM (2015). Lactate measurements in sepsis-induced tissue hypoperfusion: results from the surviving sepsis campaign database. Crit Care Med.

[7] Suetrong B, Walley KR (2016). Lactic acidosis in sepsis: it's not all anaerobic: implications for diagnosis and management. Chest.

[8] Handy JM, Soni N (2008). Physiological effects of hyperchloraemia and acidosis. Br J Anaesth.

[9] Schulz MC, Voss L, Schwerdt G, Gekle M (2022). Epithelial-fibroblast crosstalk protects against acidosis-induced inflammatory and fibrotic alterations. Biomedicines.

[10] Schulz MC, Dubourg V, Nolze A, Kopf M, Schwerdt G, Gekle M (2023). Acidosis activates the Nrf2 pathway in renal proximal tubule-derived cells through a crosstalk with renal fibroblasts. Antioxidants (Basel).

[11] Wesson DE, Buysse JM, Bushinsky DA (2020). Mechanisms of metabolic acidosis-induced kidney injury in chronic kidney disease. J Am Soc Nephrol.

[12] Kim D, Paggi JM, Park C, Bennett C, Salzberg SL (2019). Graph-based genome alignment and genotyping with HISAT2 and HISAT-genotype. Nat Biotechnol.

[13] Liao Y, Smyth GK, Shi W (2014). featureCounts: an efficient general purpose program for assigning sequence reads to genomic features. Bioinformatics.

[14] Durinck S, Spellman PT, Birney E, Huber W (2009). Mapping identifiers for the integration of genomic datasets with the R/Bioconductor package biomaRt. Nat Protoc.

[15] Robinson MD, McCarthy DJ, Smyth GK (2010). edgeR: a bioconductor package for differential expression analysis of digital gene expression data. Bioinformatics.

[16] Love MI, Huber W, Anders S (2014). Moderated estimation of fold change and dispersion for RNA-seq data with DESeq2. Genome Biol.

[17] Reimand J, Arak T, Adler P, Kolberg L, Reisberg S, Peterson H, Vilo J (2016). g:Profiler-a web server for functional interpretation of gene lists (2016 update). Nucleic Acids Res.

[18] Supek F, Bosnjak M, Skunca N, Smuc T (2011). REVIGO summarizes and visualizes long lists of gene ontology terms. PLoS ONE.

[19] Kramer A, Green J, Pollard J, Tugendreich S (2014). Causal analysis approaches in ingenuity pathway analysis. Bioinformatics.

[20] Dubourg V, Schreier B, Schwerdt G, Rabe S, Benndorf RA, Gekle M (2022). The functional interaction of EGFR with AT1R or TP in primary vascular smooth muscle cells triggers a synergistic regulation of gene expression. Cells.

[21] Debacq-Chainiaux F, Erusalimsky JD, Campisi J, Toussaint O (2009). Protocols to detect senescence-associated beta-galactosidase (SA-betagal) activity, a biomarker of senescent cells in culture and in vivo. Nat Protoc.

[22] Bergmeyer H, Bernt E. Laktat-dehydrogenase. In: Bergmeyer HU, editor. Methoden der enzymatischen Analyse. Weinheim: Verlag Chemie; 1974. pp. 607–612

[23] Gekle M, Mildenberger S (2021). Glomerular mesangial cell pH homeostasis mediates mineralocorticoid receptor-induced cell proliferation. Biomedicines.

[24] Quinn WJ, Jiao J, TeSlaa T, Stadanlick J, Wang Z, Wang L, Akimova T, Angelin A, Schafer PM, Cully MD, Perry C, Kopinski PK, Guo L, Blair IA, Ghanem LR, Leibowitz MS, Hancock WW, Moon EK, Levine MH, Eruslanov EB, Wallace DC, Baur JA, Beier UH (2020). Lactate limits T cell proliferation via the NAD(H) Redox State. Cell Rep.

[25] Alhawiti NM, Al MS, Aziz MA, Malik SS, Mohammad S (2017). TXNIP in metabolic regulation: physiological role and therapeutic outlook. Curr Drug Targets.

[26] Qu X, Sun J, Zhang Y, Li J, Hu J, Li K, Gao L, Shen L (2018). c-Myc-driven glycolysis via TXNIP suppression is dependent on glutaminase-MondoA axis in prostate cancer. Biochem Biophys Res Commun.

[27] Chen S, Sang N (2016). Hypoxia-inducible factor-1: a critical player in the survival strategy of stressed cells. J Cell Biochem.

[28] Villa-Bellosta R (2018). Synthesis of extracellular pyrophosphate increases in vascular smooth muscle cells during phosphate-induced calcification. Arterioscler Thromb Vasc Biol.

[29] Stefan C, Stalmans W, Bollen M (1996). Threonine autophosphorylation and nucleotidylation of the hepatic membrane protein PC-1. Eur J Biochem.

[30] Garcia-Alvarez M, Marik P, Bellomo R (2014). Sepsis-associated hyperlactatemia. Crit Care.

[31] Wong HR, Lindsell CJ, Pettila V, Meyer NJ, Thair SA, Karlsson S, Russell JA, Fjell CD, Boyd JH, Ruokonen E, Shashaty MG, Christie JD, Hart KW, Lahni P, Walley KR (2014). A multibiomarker-based outcome risk stratification model for adult septic shock*. Crit Care Med.

[32] Wardi G, Brice J, Correia M, Liu D, Self M, Tainter C (2020). Demystifying lactate in the emergency department. Ann Emerg Med.

[33] Singer M (2014). The role of mitochondrial dysfunction in sepsis-induced multi-organ failure. Virulence.

[34] Lee TY (2021). Lactate: a multifunctional signaling molecule. Yeungnam Univ J Med.

[35] Langley RJ, Tsalik EL, van Velkinburgh JC, Glickman SW, Rice BJ, Wang C, Chen B, Carin L, Suarez A, Mohney RP, Freeman DH, Wang M, You J, Wulff J, Thompson JW, Moseley MA, Reisinger S, Edmonds BT, Grinnell B, Nelson DR, Dinwiddie DL, Miller NA, Saunders CJ, Soden SS, Rogers AJ, Gazourian L, Fredenburgh LE, Massaro AF, Baron RM, Choi AM, Corey GR, Ginsburg GS, Cairns CB, Otero RM, Fowler VG, Rivers EP, Woods CW, Kingsmore SF (2013). An integrated clinico-metabolomic model improves prediction of death in sepsis. Sci Transl Med.

[36] Wu N, Zheng B, Shaywitz A, Dagon Y, Tower C, Bellinger G, Shen CH, Wen J, Asara J, McGraw TE, Kahn BB, Cantley LC (2013). AMPK-dependent degradation of TXNIP upon energy stress leads to enhanced glucose uptake via GLUT1. Mol Cell.

[37] Trivedi B, Danforth WH (1966). Effect of pH on the kinetics of frog muscle phosphofructokinase. J Biol Chem.

[38] Kappler M, Pabst U, Weinholdt C, Taubert H, Rot S, Kaune T, Kotrba J, Porsch M, Guttler A, Bache M, Krohn K, Bull F, Riemann A, Wickenhauser C, Seliger B, Schubert J, Al-Nawas B, Thews O, Grosse I, Vordermark D, Eckert AW (2019). Causes and consequences of a glutamine induced normoxic HIF1 activity for the tumor metabolism. Int J Mol Sci.

[39] Sun F, Dai C, Xie J, Hu X (2012). Biochemical issues in estimation of cytosolic free NAD/NADH ratio. PLoS ONE.

[40] Kelly RA, Leedale J, Harrell A, Beard DA, Randle LE, Chadwick AE, Webb SD (2018). Modelling the impact of changes in the extracellular environment on the cytosolic free NAD+/NADH ratio during cell culture. PLoS ONE.

[41] Barron JT, Gu L, Parrillo JE (2000). NADH/NAD redox state of cytoplasmic glycolytic compartments in vascular smooth muscle. Am J Physiol Heart Circ Physiol.

[42] Fuller GG, Kim JK (2021). Compartmentalization and metabolic regulation of glycolysis. J Cell Sci.

[43] Arnon S, Litmanovits I, Regev R, Elpeleg O, Dolfin T (2001). Dichloroacetate treatment for severe refractory metabolic acidosis during neonatal sepsis. Pediatr Infect Dis J.

[44] McCall CE, Zhu X, Zabalawi M, Long D, Quinn MA, Yoza BK, Stacpoole PW, Vachharajani V (2022). Sepsis, pyruvate, and mitochondria energy supply chain shortage. J Leukoc Biol.

[45] Ibrahim D, Prevaud L, Faumont N, Troutaud D, Feuillard J, Diab-Assaf M, Oulmouden A (2022). Alternative c-MYC mRNA transcripts as an additional tool for c-Myc2 and c-MycS production in BL60 tumors. Biomolecules.

[46] Ribeiro-Silva JC, Nolasco P, Krieger JE, Miyakawa AA (2021). Dynamic crosstalk between vascular smooth muscle cells and the aged extracellular matrix. Int J Mol Sci.

[47] Nitschke Y, Rutsch F (2017). Inherited arterial calcification syndromes: etiologies and treatment concepts. Curr Osteoporos Rep.

[48] Lang F, Leibrock C, Pelzl L, Gawaz M, Pieske B, Alesutan I, Voelkl J (2018). therapeutic interference with vascular calcification-lessons from klotho-hypomorphic mice and beyond. Front Endocrinol (Lausanne).

[49] Hofmann Bowman MA, McNally EM (2012). Genetic pathways of vascular calcification. Trends Cardiovasc Med.

[50] Inoue A, Nakao-Kuroishi K, Kometani-Gunjigake K, Mizuhara M, Shirakawa T, Ito-Sago M, Yasuda K, Nakatomi M, Matsubara T, Tada-Shigeyama Y, Morikawa K, Kokabu S, Kawamoto T (2020). VNUT/SLC17A9, a vesicular nucleotide transporter, regulates osteoblast differentiation. FEBS Open Bio.

[51] Fujimoto K, Shioi A, Miki Y, Kakutani Y, Morioka T, Shoji T, Emoto M, Inaba M (2019). Adenosine attenuates aortic smooth muscle cell calcification through A(3) adenosine receptor. Tohoku J Exp Med.

[52] Gan XT, Taniai S, Zhao G, Huang CX, Velenosi TJ, Xue J, Urquhart BL, Karmazyn M (2014). CD73-TNAP crosstalk regulates the hypertrophic response and cardiomyocyte calcification due to alpha1 adrenoceptor activation. Mol Cell Biochem.

[53] Iwashyna TJ, Ely EW, Smith DM, Langa KM (2010). Long-term cognitive impairment and functional disability among survivors of severe sepsis. JAMA.

