# Supplementary material for: The synergism of cytosolic acidosis and reduced NAD+/NADH ratio is responsible for lactic acidosis-induced vascular smooth muscle cell impairment in sepsis
Source: J Biomed Sci. 2024 Jan 9;31:3. doi: 10.1186/s12929-023-00992-6 (PMC10775599; doi:10.1186/s12929-023-00992-6)
Supplement: Supplementary file 1 — Additional file 1: Figure S1. Cell viability after 24 h. Figure S2. Cell viability after 72 h. Figure S3. Acidosis-induced changes of the HAoSMC transcriptome. Figure S4. Determination of the cellular NAD+ and NADH concentration. Figure S5. Expression changes of genes involved in metabolic regulation. Figure S6. Expression changes of additional calcification-associated genes. Figure S7. Expression changes of osteochondrogenic genes. Figure S8. Expression changes of NAD+-consuming enzymes. Figure S9. Expression changes of genes involved in the malate-aspartate shuttle. Table S1. upstream regulator (UR) for DEGLA and DEGHCl which were identified by IPA. Table S2. g:Profler multiquery to compare the putative GO term enrichment of the two sets of predicted UR. Table S3. Disease and biofunction (DBF) analysis which were generated by IPA. Table S4. DEGLA-NOT-DEGHCl set (lactacidosis-specific effect) by IPA. Table S5. GO term enrichment analysis of the UR results. [file 12929_2023_992_MOESM1_ESM.docx]

**Additional file 1**

**The synergism of cytosolic acidosis and reduced NAD^+^/NADH ratio is responsible for lactacidosis-induced vascular smooth muscle cell impairment in sepsis.**

Philipp Terpe^1,2^ #, Stefanie Ruhs^2^ #, Virginie Dubourg^1^, Michael Bucher^2^ and Michael Gekle^1^

^1^ Julius-Bernstein-Institute of Physiology, Martin Luther University Halle-Wittenberg, 06112 Halle (Saale), Germany

^2^ Department of Anesthesiology and Surgical Intensive Care, University Hospital Halle (Saale), 06120 Halle (Saale), Germany

# equal contribution

*** Corresponding author:** Stefanie.ruhs@medizin.uni-halle.de

**Additional Figures/Tables**

**Additional Figure S1: Cell viability after 24 h**

**
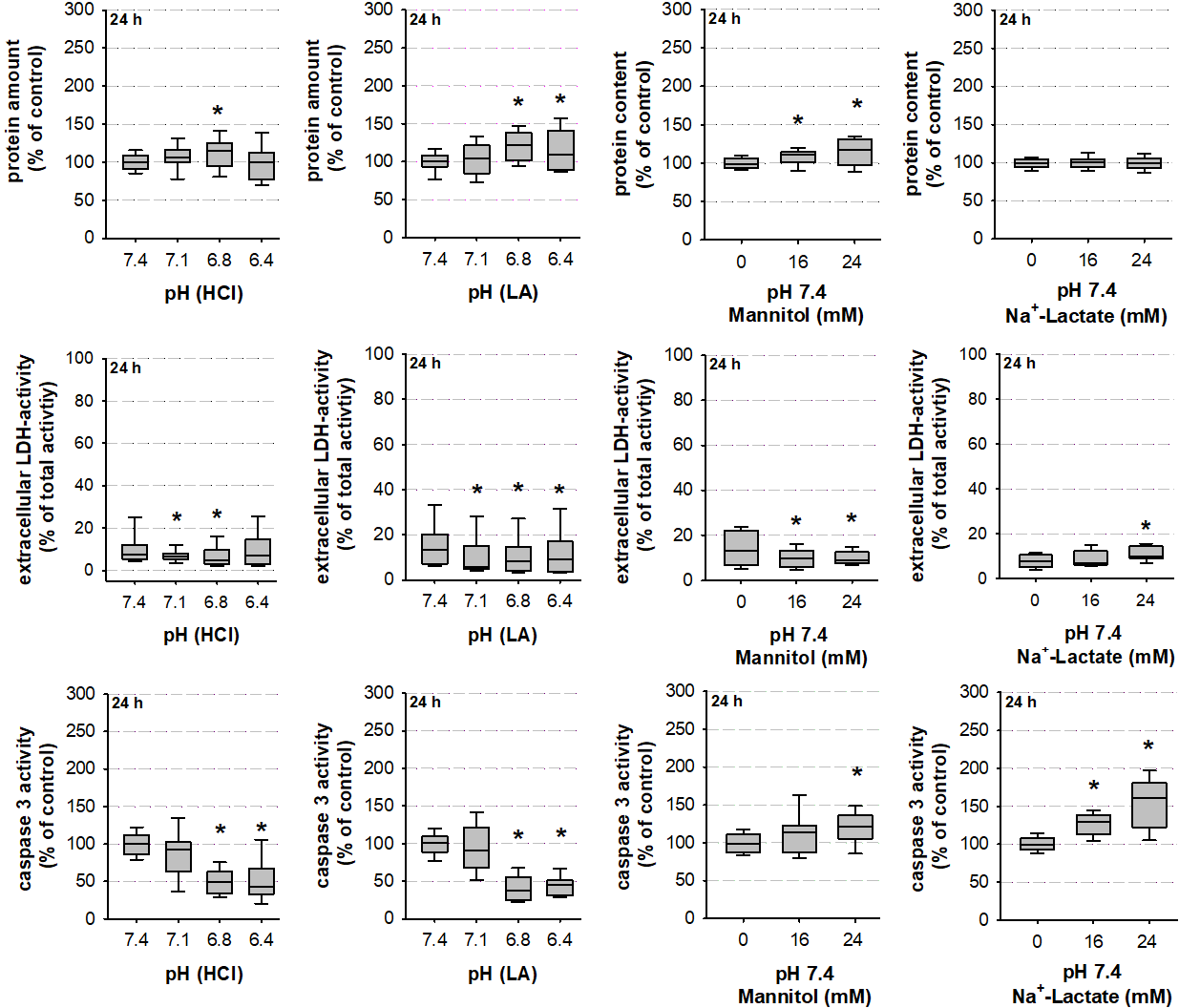
**

Cellular viability was determined by protein content, LDH release, and caspase activity after 24 h of incubation by a pH value range from 7.4 to 6.4. The treatment conditions did not decrease cellular protein content (N=4-10, n=16-28), nor induce necrotic (N=4-9, n=16-26) or noteworthy apoptotic cell death (N=4-10, n=15-29); * p ≤ 0.05 vs. ctrl_pH 7.4.

**Additional Figure S2: Cell viability after 72 h**

**
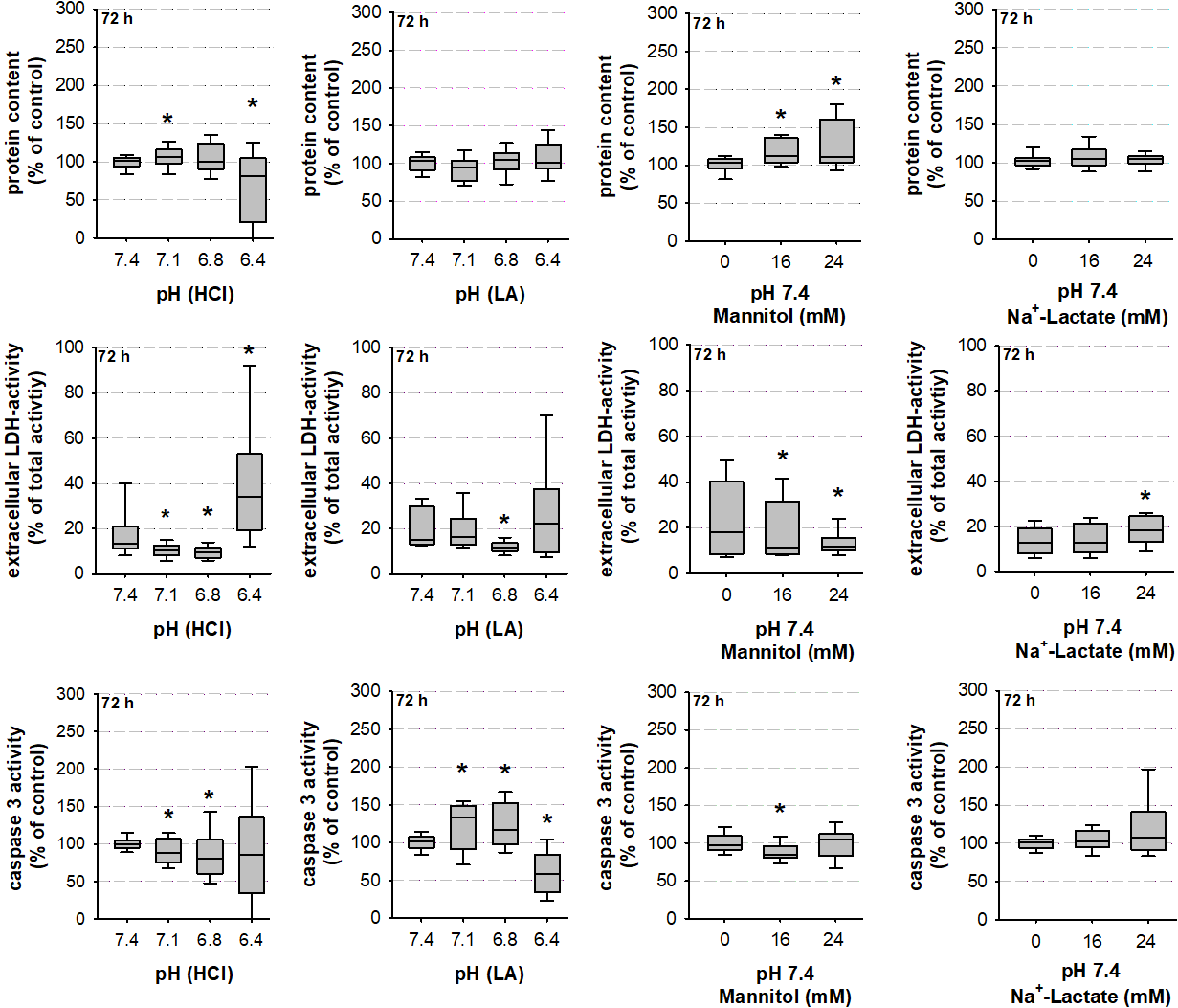
**

Cellular viability was determined by protein content, LDH release, and caspase activity after 72 h of incubation by a pH value range from 7.4 to 6.4. The treatment conditions did not decrease cellular protein content (N=4-10, n=15-30), nor induce necrotic (N=4-10, n=14-30) or noteworthy apoptotic cell death except the pH value of 6.4 (N=3-10, n=12-29); *p ≤ 0.05 vs. ctrl_pH 7.4.

**Additional Figure S3:**

**Acidosis-induced changes of the HAoSMC transcriptome**

**
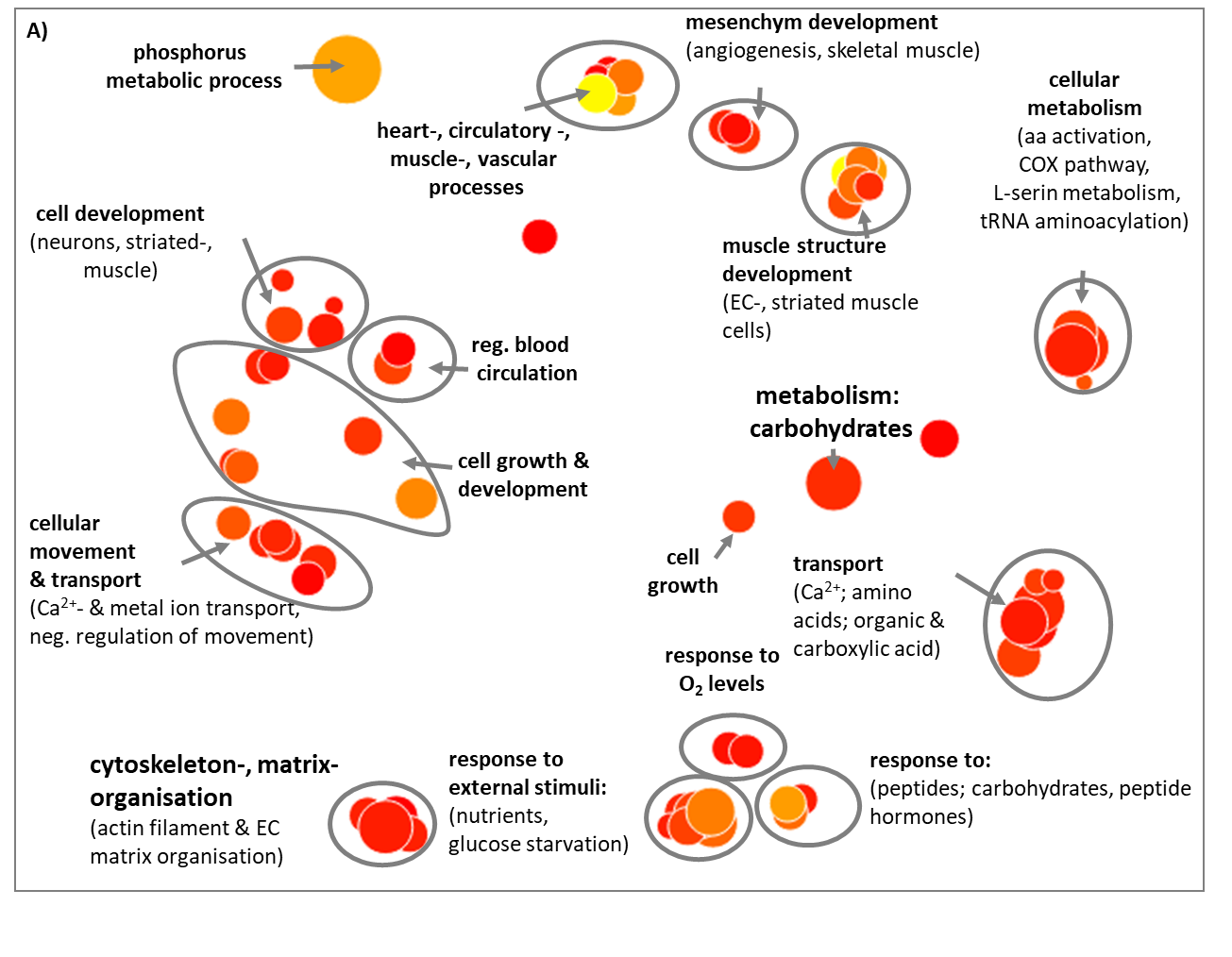
**

**B)**

**
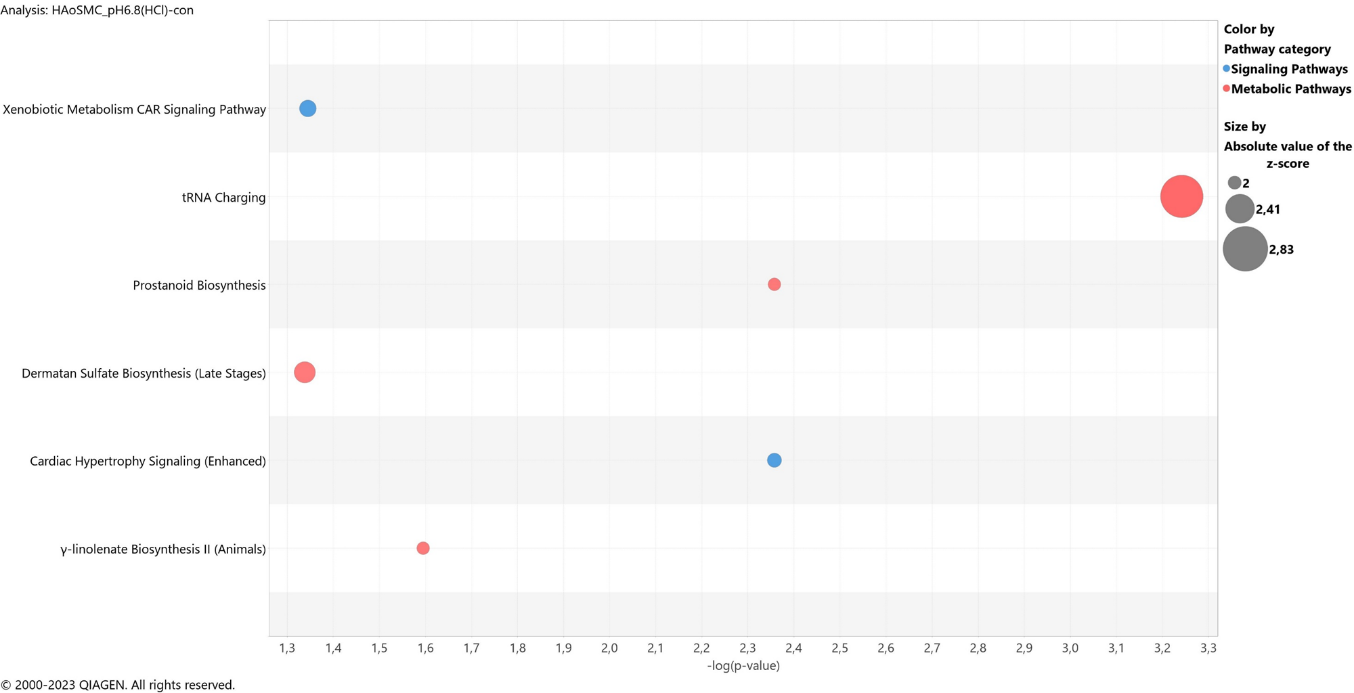
**

**
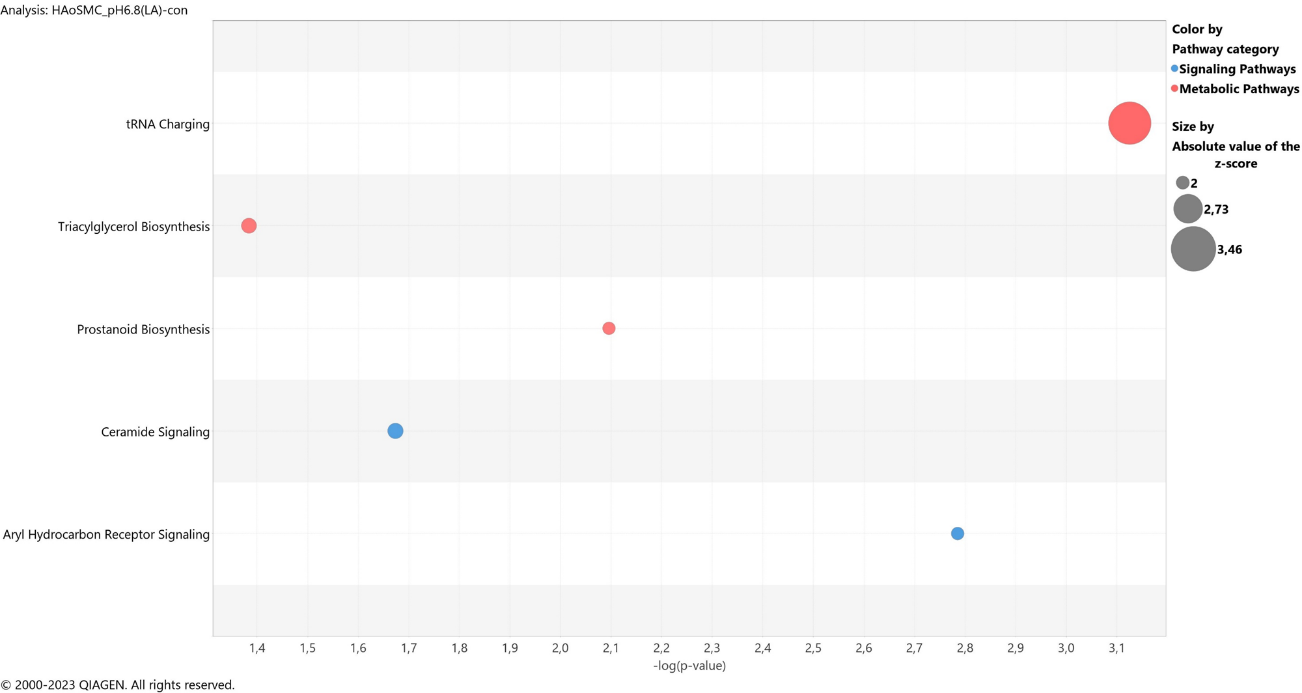
**

**C)**

**
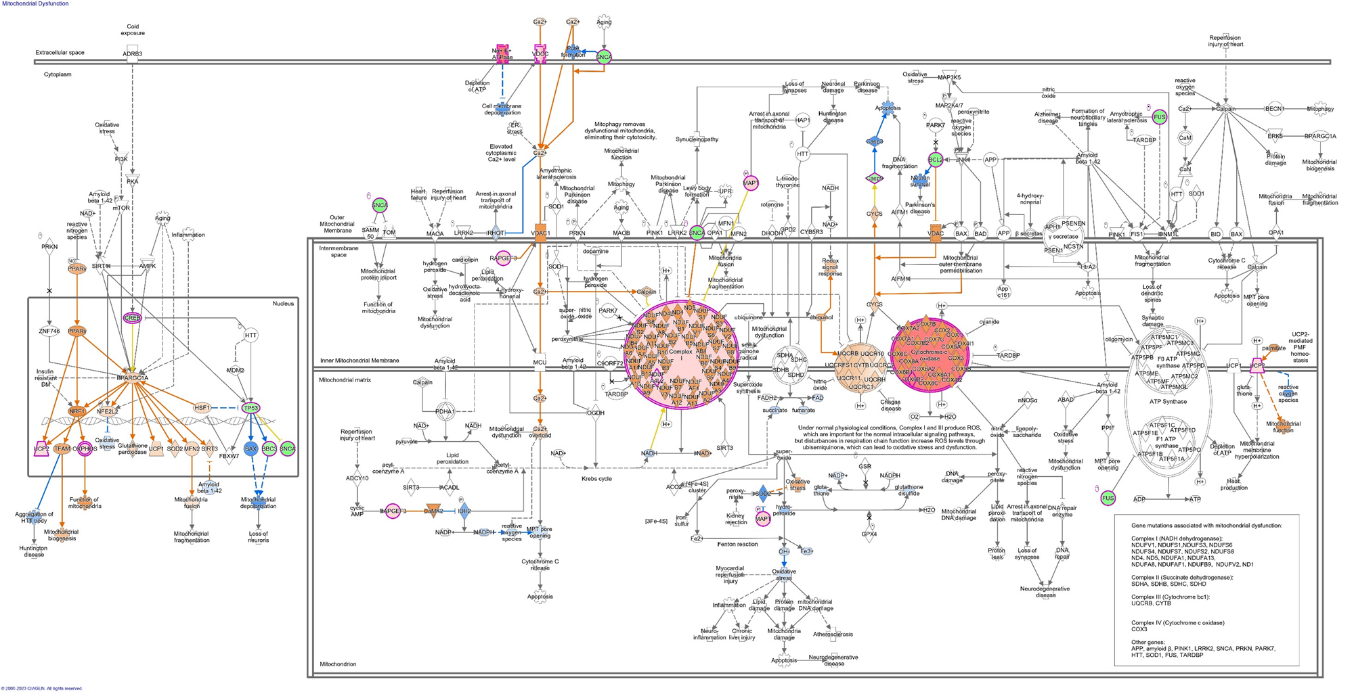
**

**D)**

**
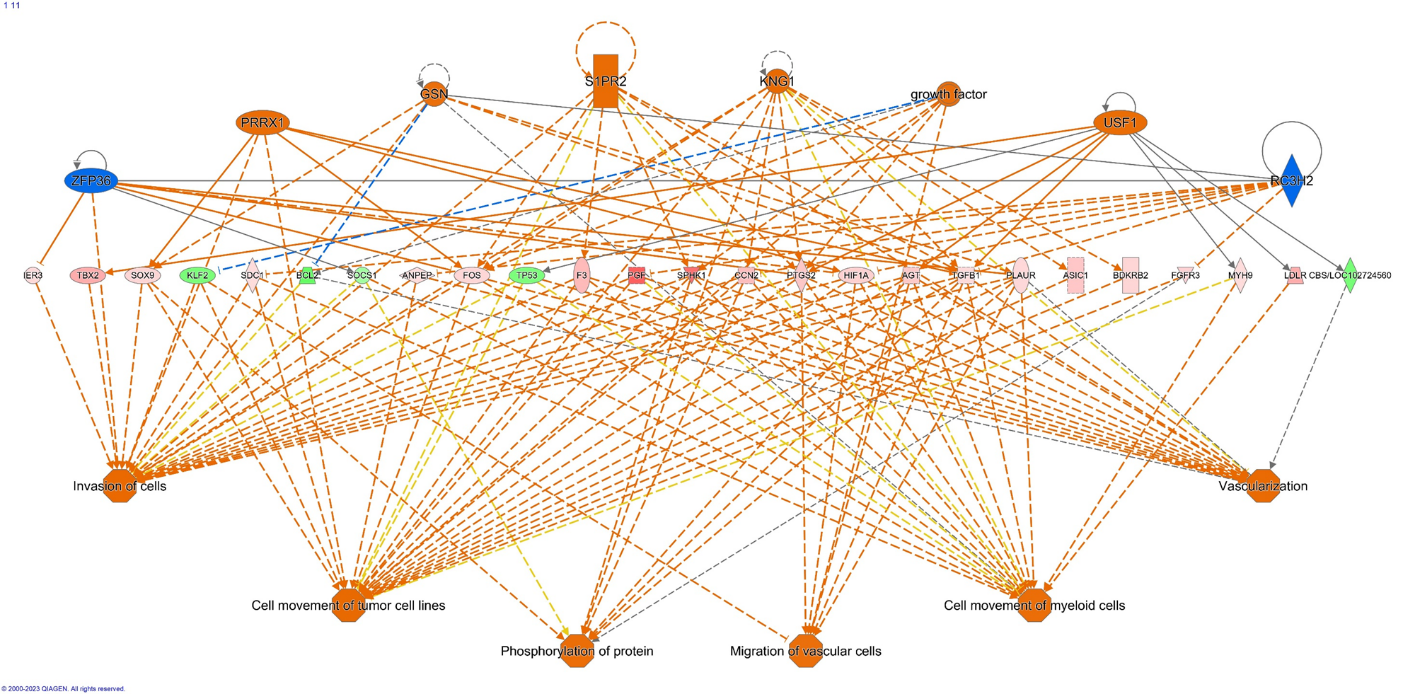
**

**
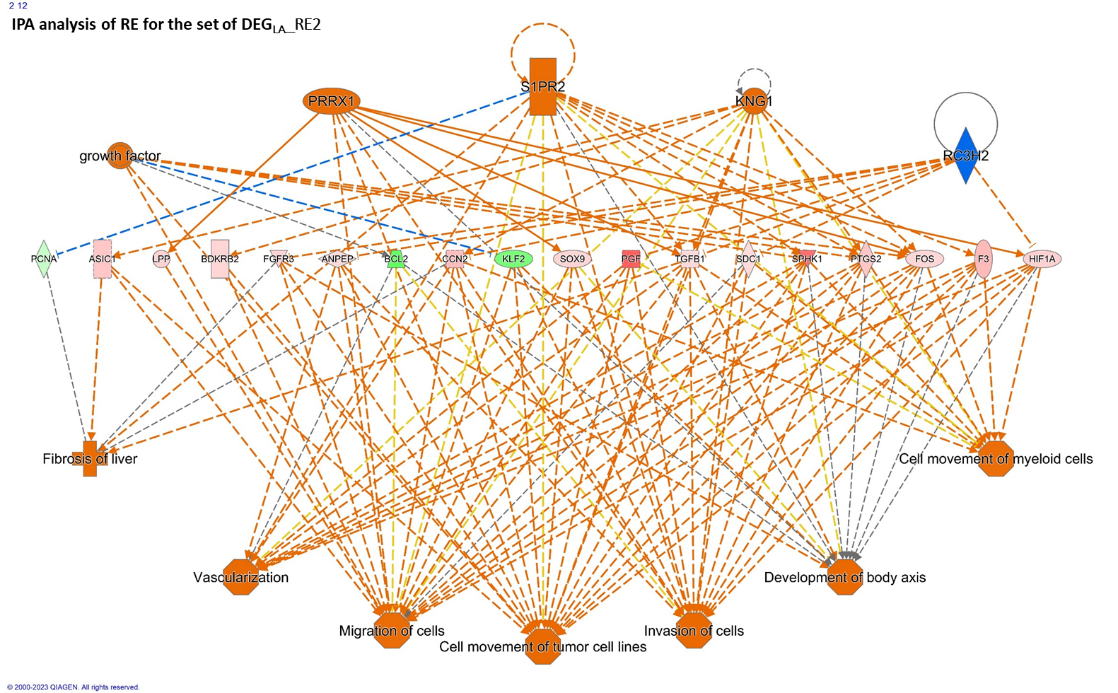
**

**
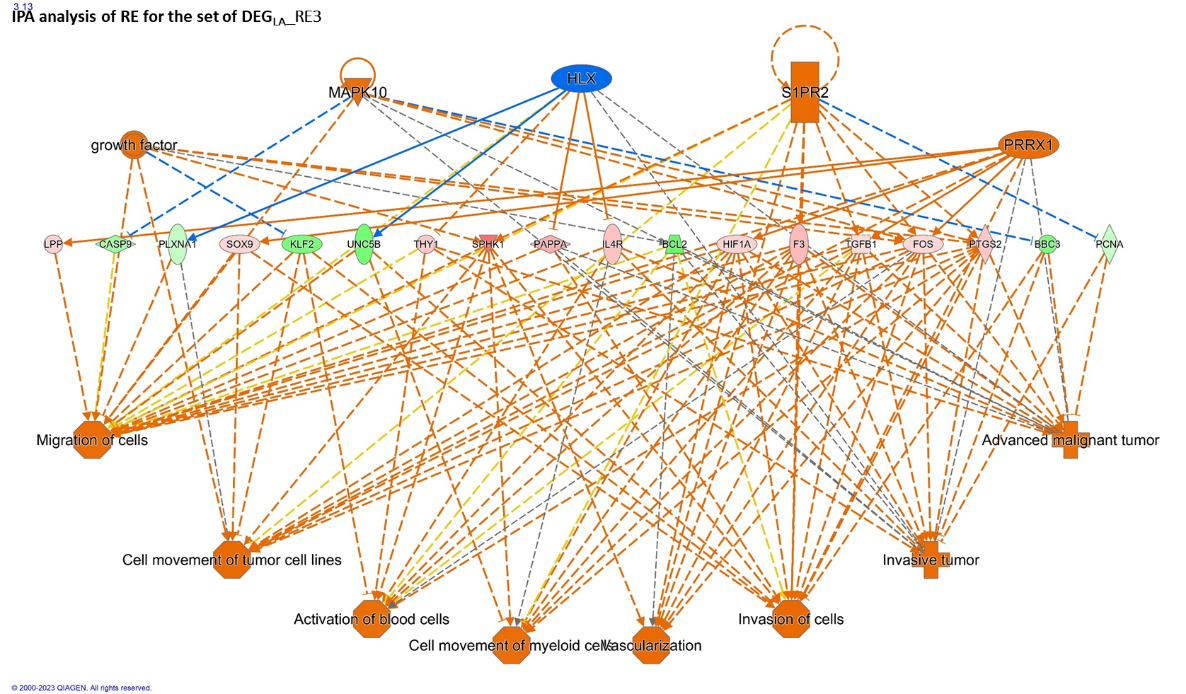
**

**(A)** Illustration of acidosis-induced biological processes which were induced for both HCl and LA treatment obtained by GO term enrichment analysis with gprofiler (Benjamini-Hochberg FDR p<0.001; -log (p)>2; enrichment E >2.5) using Revigo. The LogSize scale indicates by size the included GO terms (the larger the more GO terms) and the color represents the significance (the more yellow the more significant). Acidosis per se resulted in an altered metabolism (carbohydrates, L-Serin, aa, COX) and dedifferentiation of HAoSMCs (phosphorus metabolic process, cell structure, cytoskeleton, EZM organisation).

**(B)** The data sets of DEG_HCl_ and DEG_LA_ were analysed for CP by IPA showing only a few pathways.

**(C)** The data set for DEG_LA_-NOT-DEG_HCl_ was analysed by IPA resulting in only one CP associated with mitochondrial dysfunction (Z-score = - 1.75).

**(D)** The data set for regulatory elements predication (RE) analysis for DEG_LA_ by IPA shows three predicted RE (RE1-RE3).

**Additional Figure S4:**

**Determination of the cellular NAD^+^ and NADH concentration**

Shown are the cellular NAD^+^ and NADH concentrations, which were determined after 48 h treatment. Lactic acid as well as Na^+^-lactate treatment led to a reduced concentration of NAD+ whereas the concentration of NADH increased. (N = 5, n = 14-15; *p ≤ 0.05 vs. ctrl_pH 7.4; #p ≤ 0.05 as indicated).

**Additional Figure S5:**

**Expression changes of genes involved in metabolic regulation.**

**
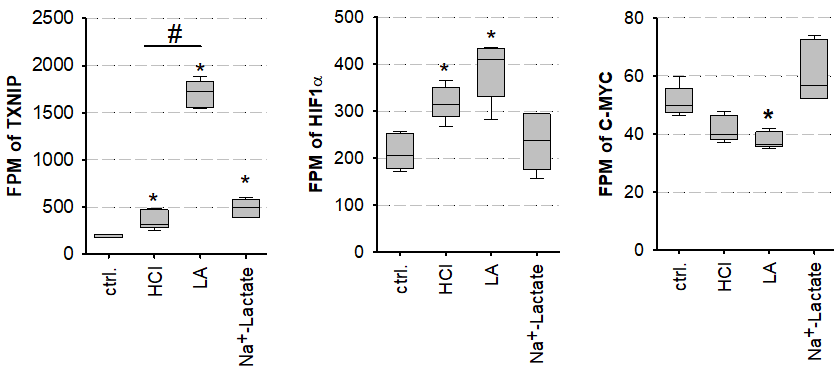
**

Shown are the FPMs obtained by RNA sequencing for TXNIP, HIF1α and c-Myc, genes involved in metabolic regulation. Lactacidosis led to a strong upregulation of TXNIP mRNA whereas C-MYC mRNA was significantly downregulated. HIF1α mRNA expression was increased by both types of acidosis (N = 5, n = 5; *p ≤ 0.05 vs. ctrl_pH 7.4; #p ≤ 0.05 HCl vs. LA).

**Additional figure S6:**

**Expression changes of additional calcification-associated genes.**


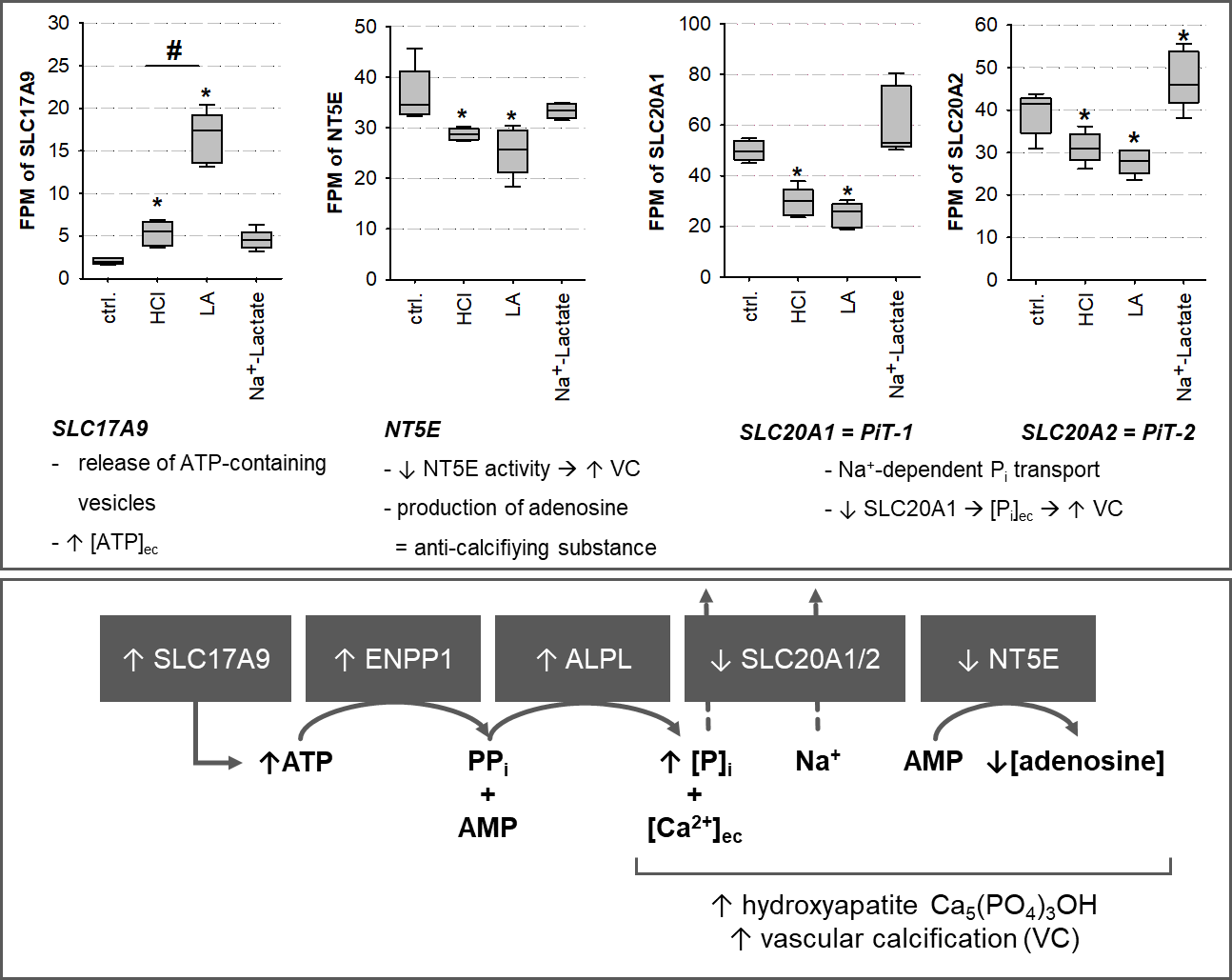


Shown are the FPMs obtained by RNA sequencing for genes involved in the vascular calcification process and a schematic showing the calcification process in more detail. Acidosis resulted in upregulation of SLC17A9 mRNA, while NT5E and SLC20A1/A2 mRNA were downregulated (N = 5, n = 5; *p ≤ 0.05 vs. ctrl_pH 7.4; # p ≤ 0.05 HCl vs.LA). Reduced NT5E expression may lead to decreased production of the anti-calcifying substance adenosine. In addition, reduced expression of the sodium-coupled P_i_ transporters SLC20A1/2 may lead to an increased accumulation of P_i_ in the extracellular space. Both processes favour and enhance VC

**Additional figure S7:**

**Expression changes of osteochondrogenic genes.**


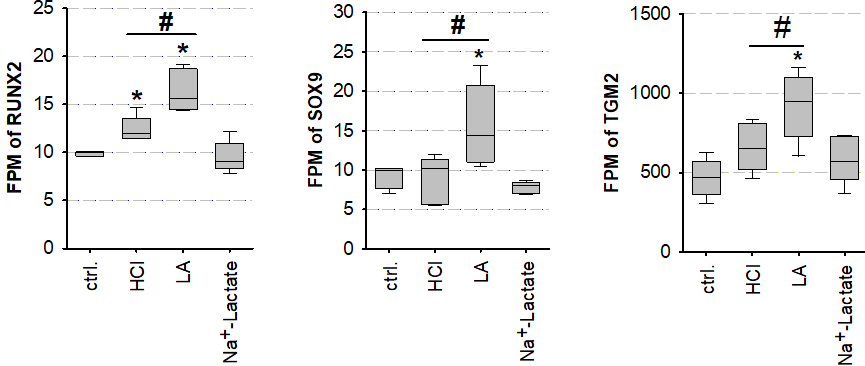


Shown are the FPMs obtained by RNA sequencing for genes involved in dedifferentiation of VSMC to osteo-/chondrogenic-like cells. Lactacidosis led to an upregulation of RUNX2-, SOX9 and TGM2 mRNA (N = 5, n = 5; *p ≤ 0.05 vs. ctrl_pH 7.4; # p ≤ 0.05 HCl vs.LA).

**Additional figure S8:**

**Expression changes of NAD^+^-consuming enzymes.**


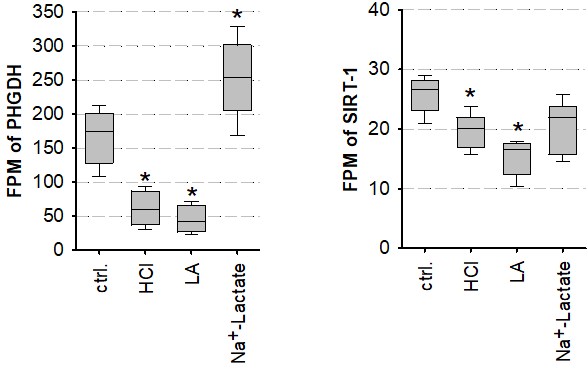


Shown are the FPMs obtained by RNA sequencing for genes encoding NAD^+^-consuming enzymes. Acidosis led to a downregulation of PHGDH and SIRT-1 mRNA (N = 5, n = 5; *p ≤ 0.05 vs. ctrl_pH 7.4).

**Additional figure S9:**

**Expression changes of genes involved in the malate-aspartate shuttle.**

Shown are the FPMs obtained by RNA sequencing for genes involved in the malate-aspartate shuttle. Acidosis led to a downregulation of SLC25A12, SLC25A13 and GOT1 mRNA (N = 5, n = 5; *p ≤ 0.05 vs. ctrl_pH 7.4).

**Additional tables**

**Additional table S1**

**
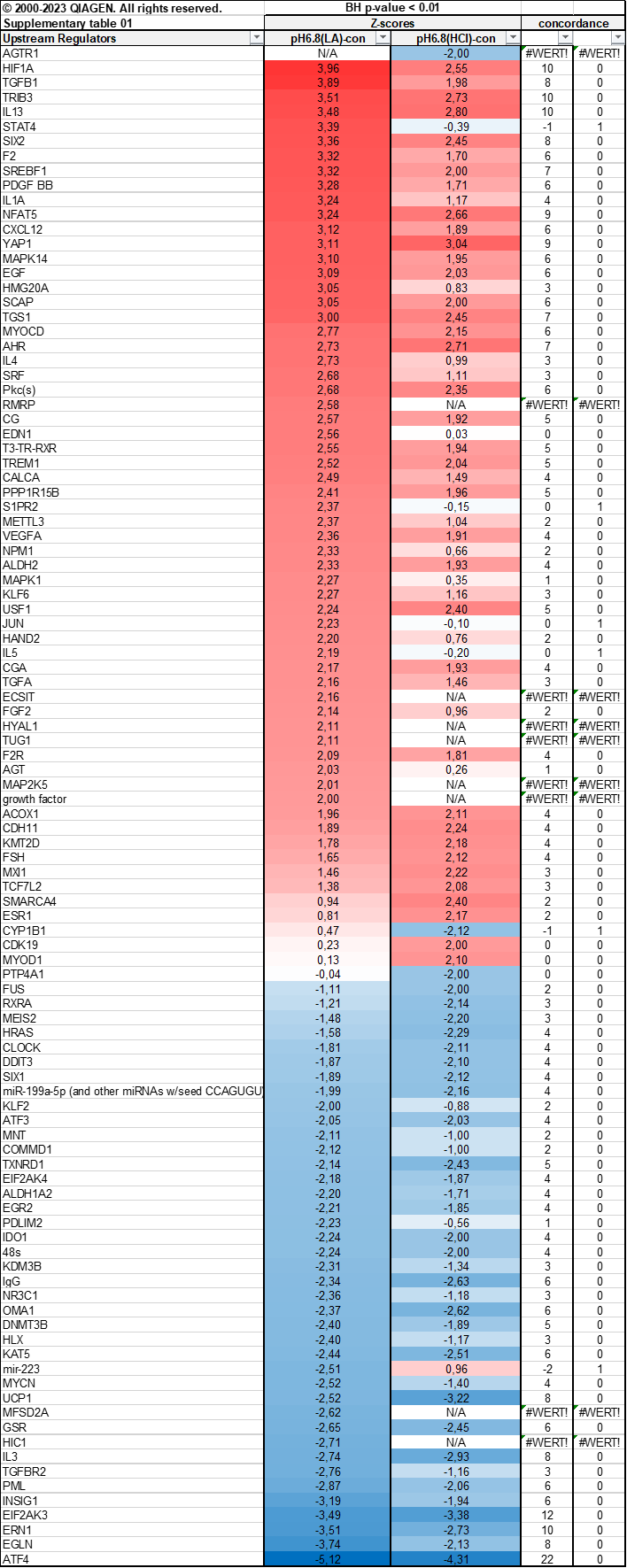
**

**
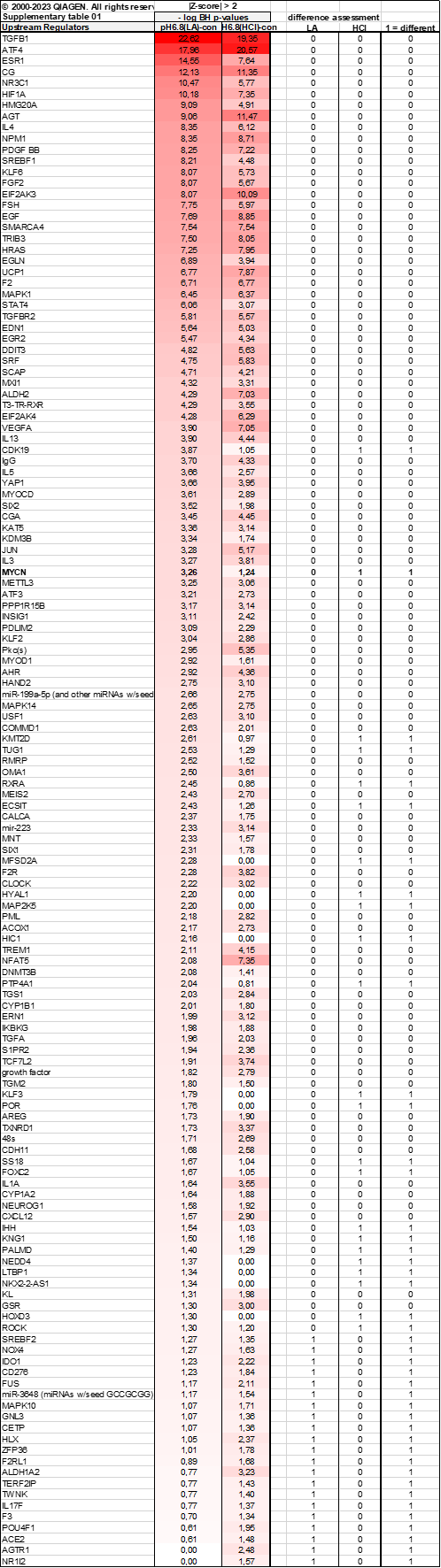
**

Shown are the upstream regulator (UR) for DEG_LA_ and DEG_HCl_ which were identified by IPA.

**Additional table S2**


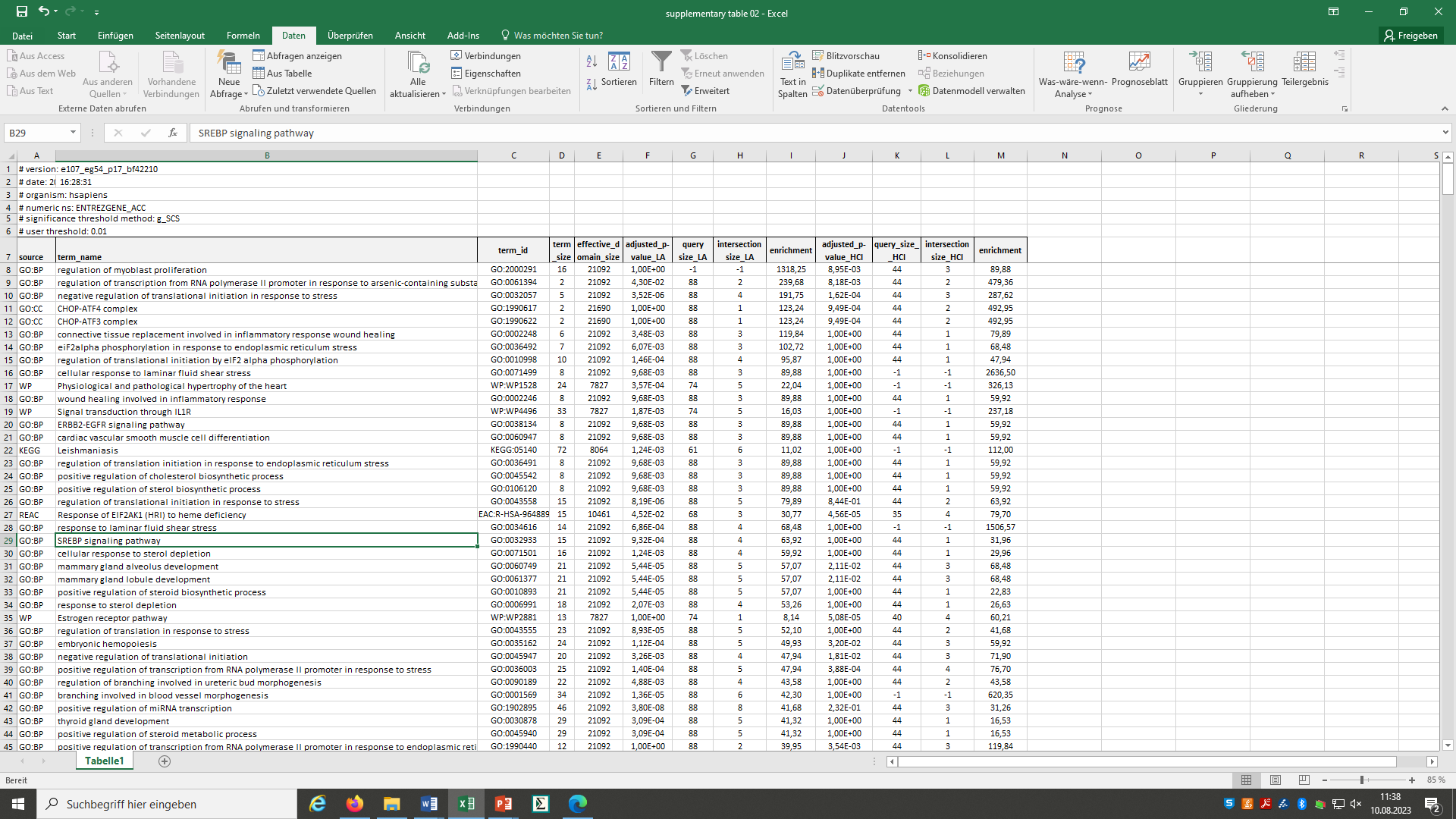

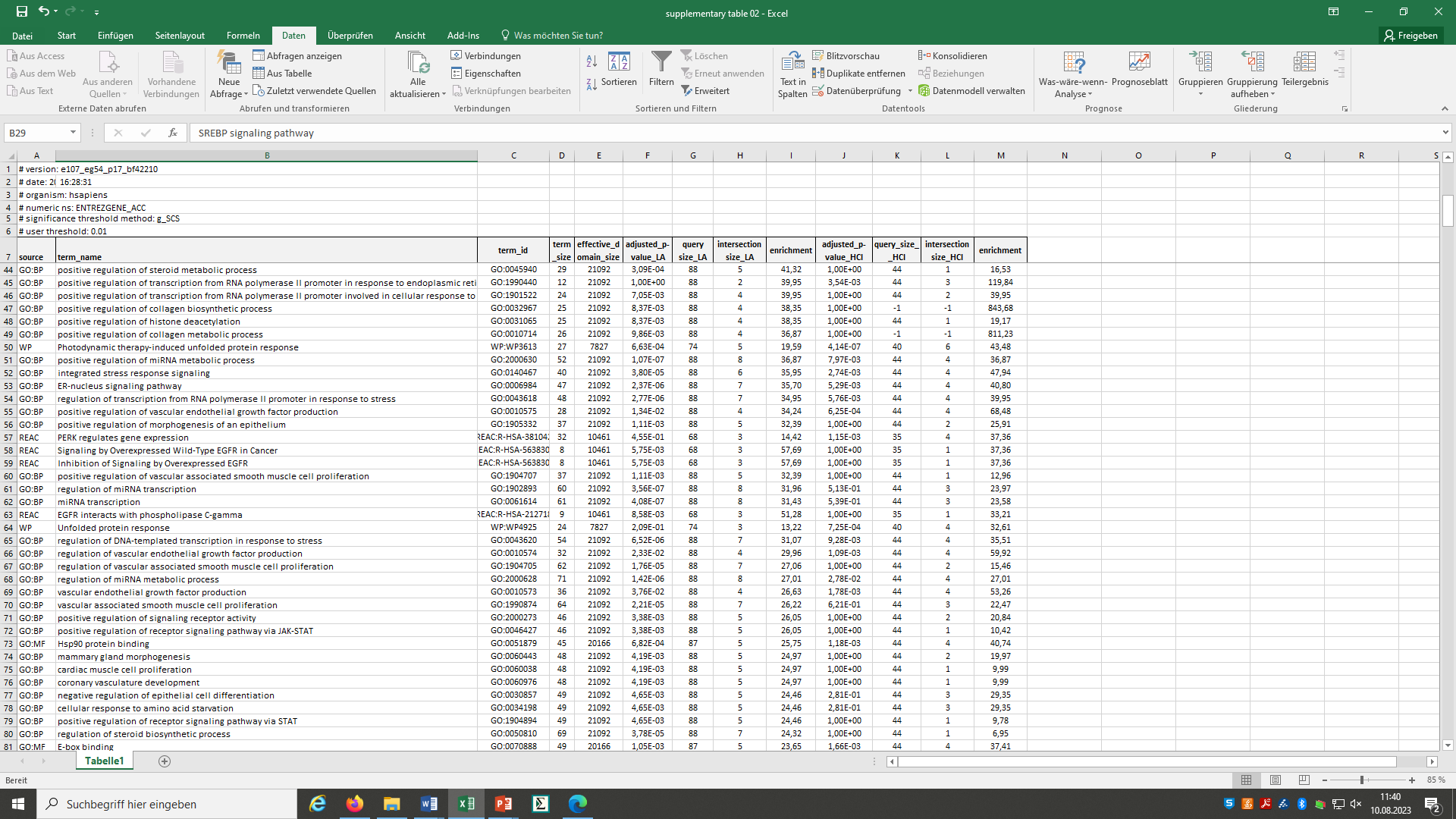

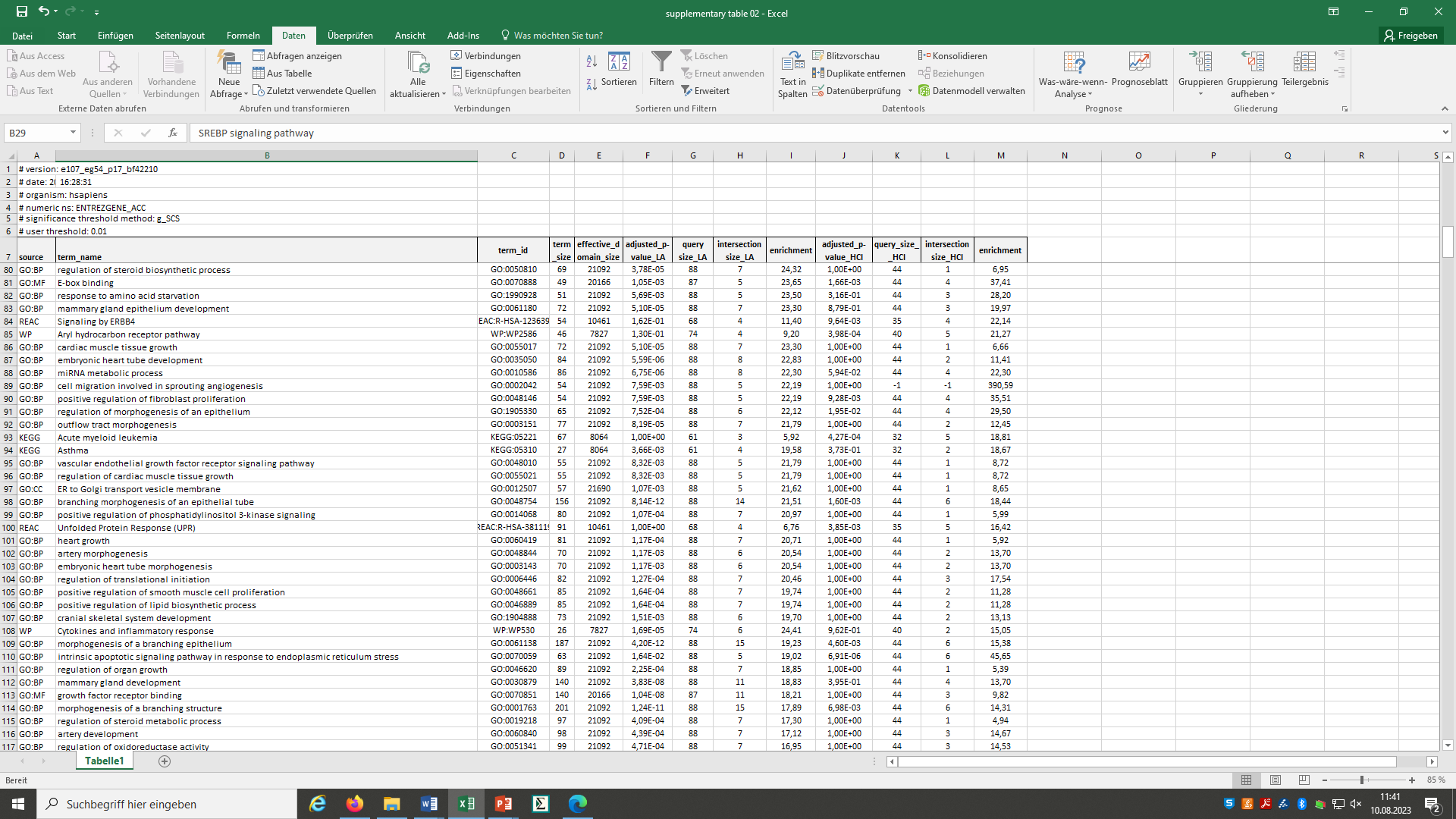

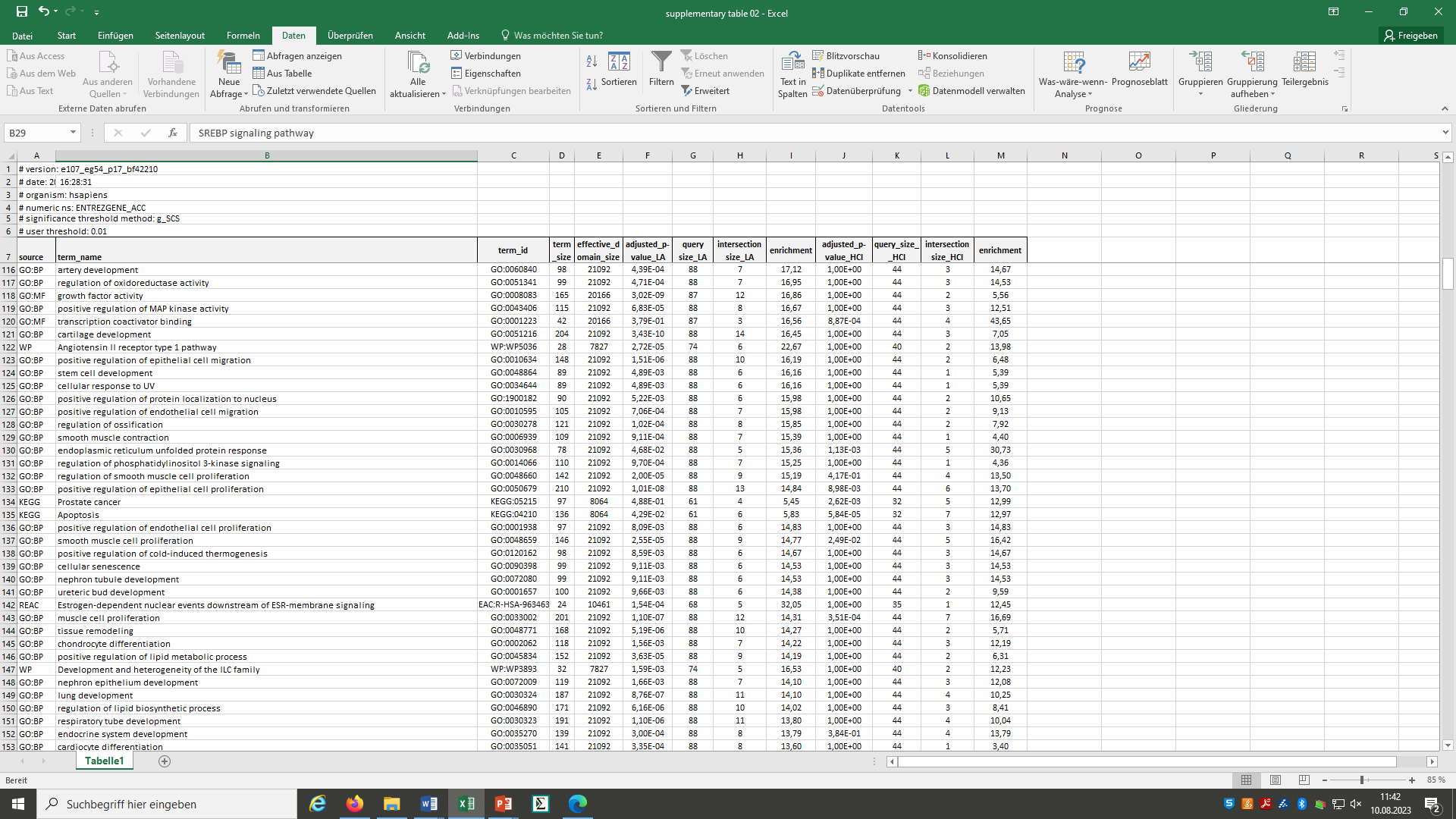


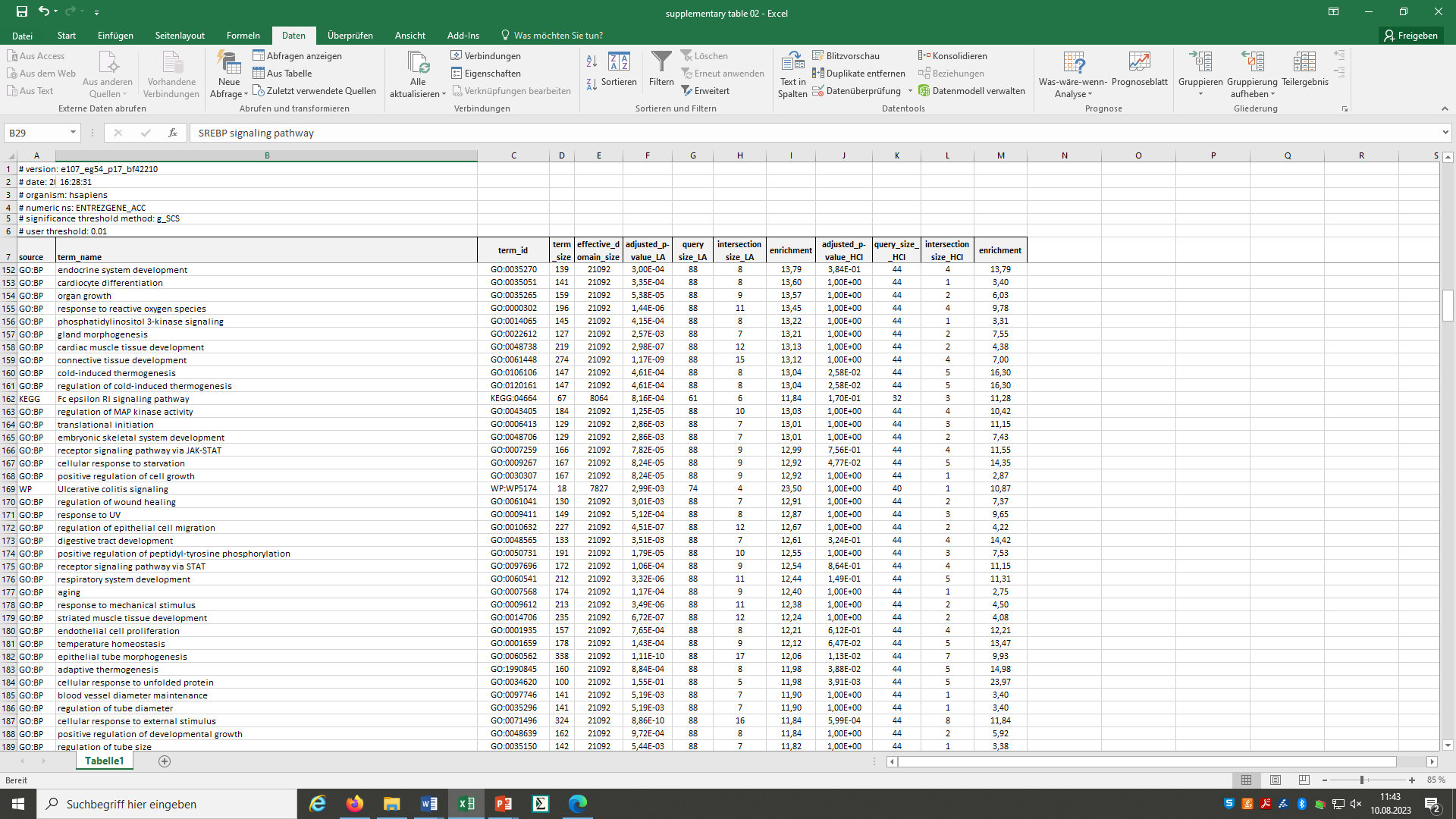

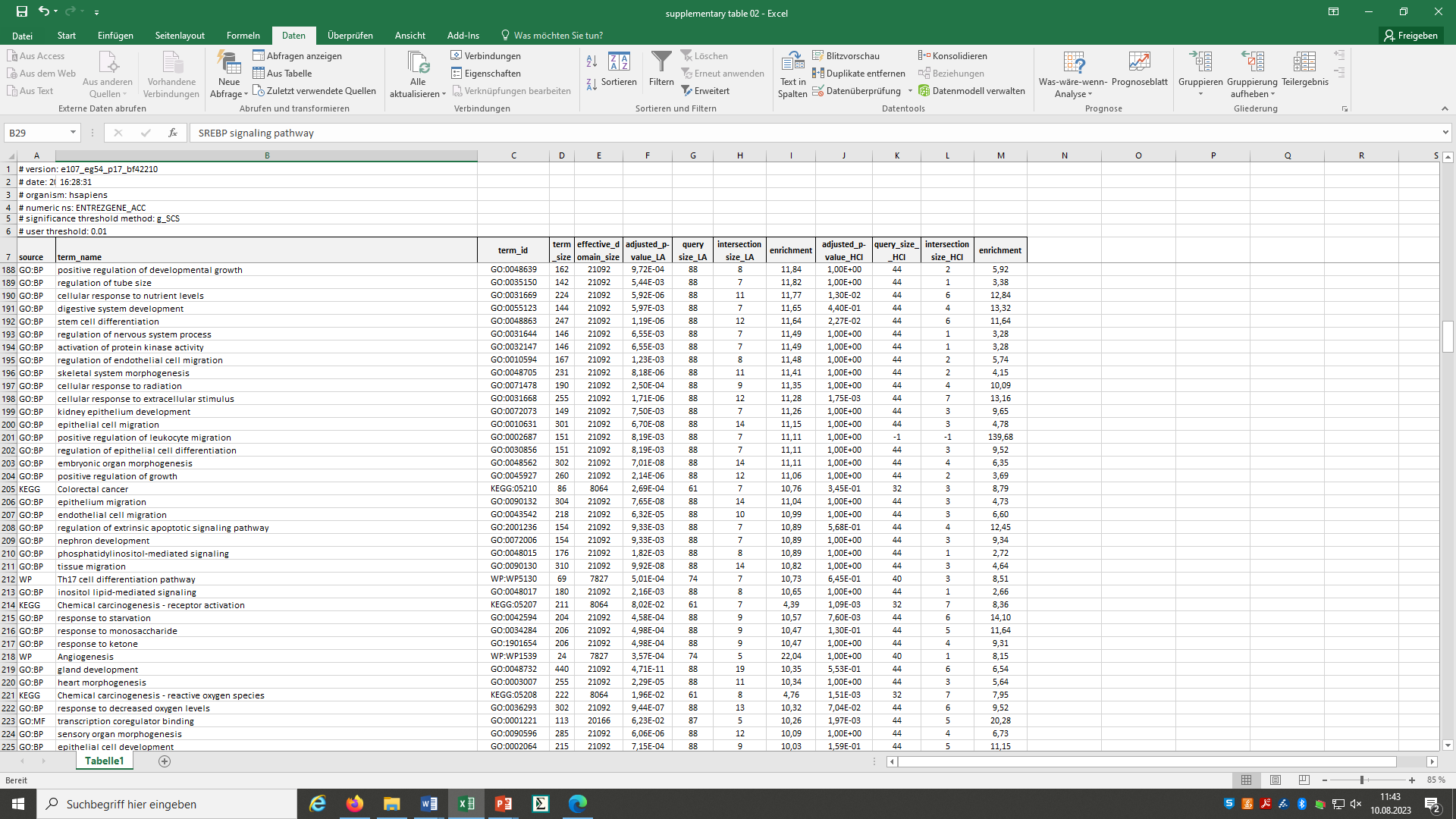

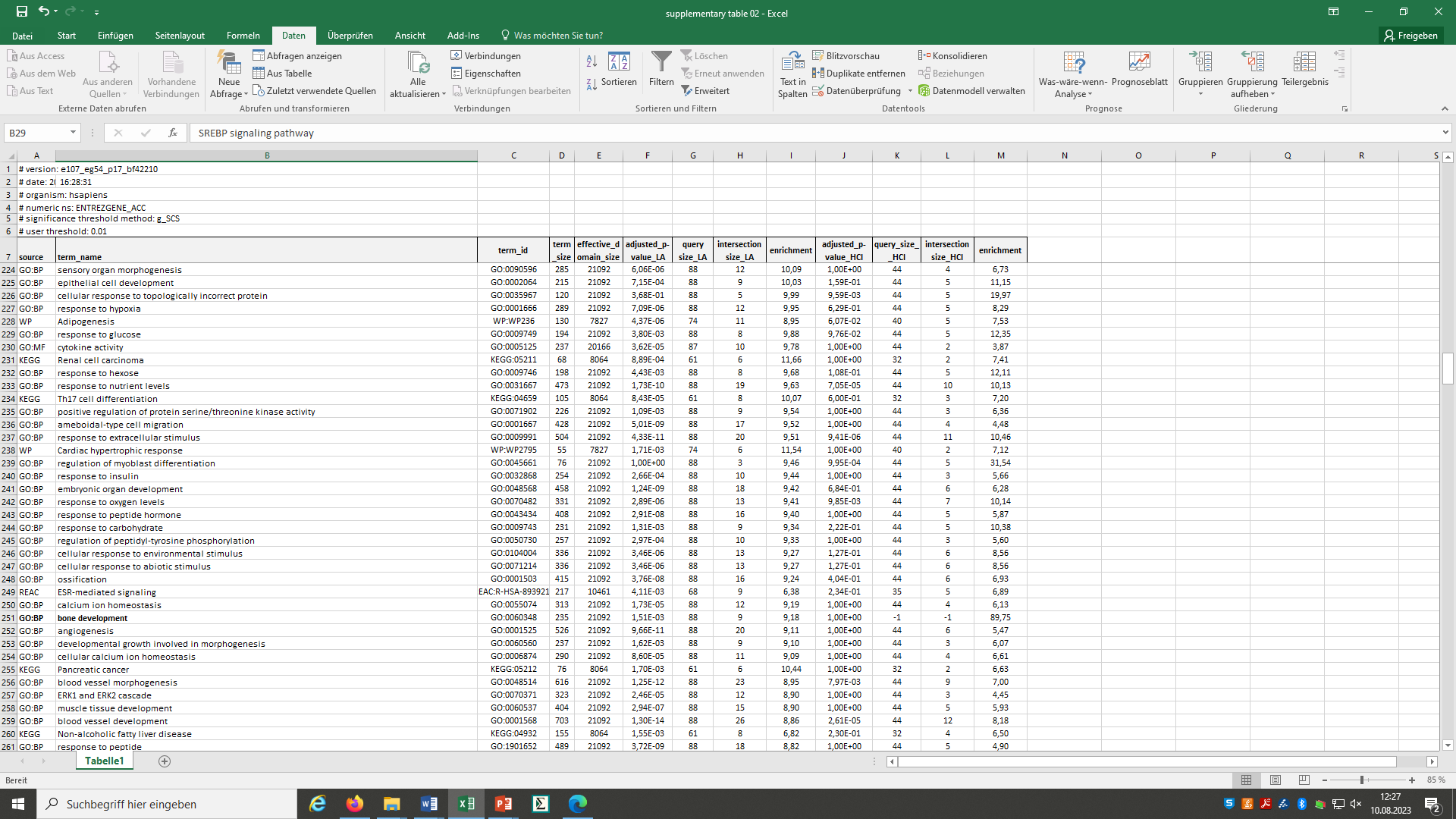

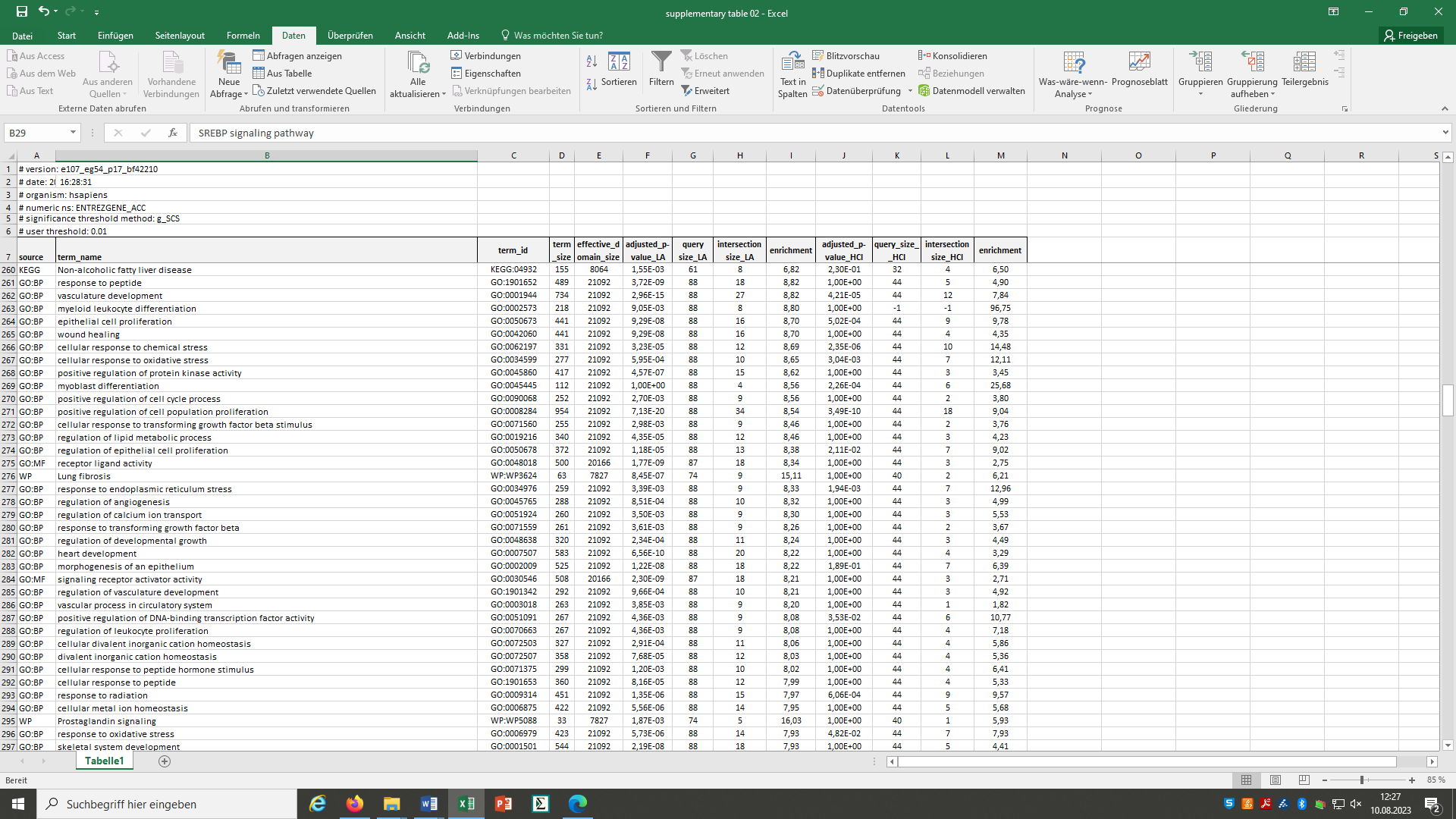


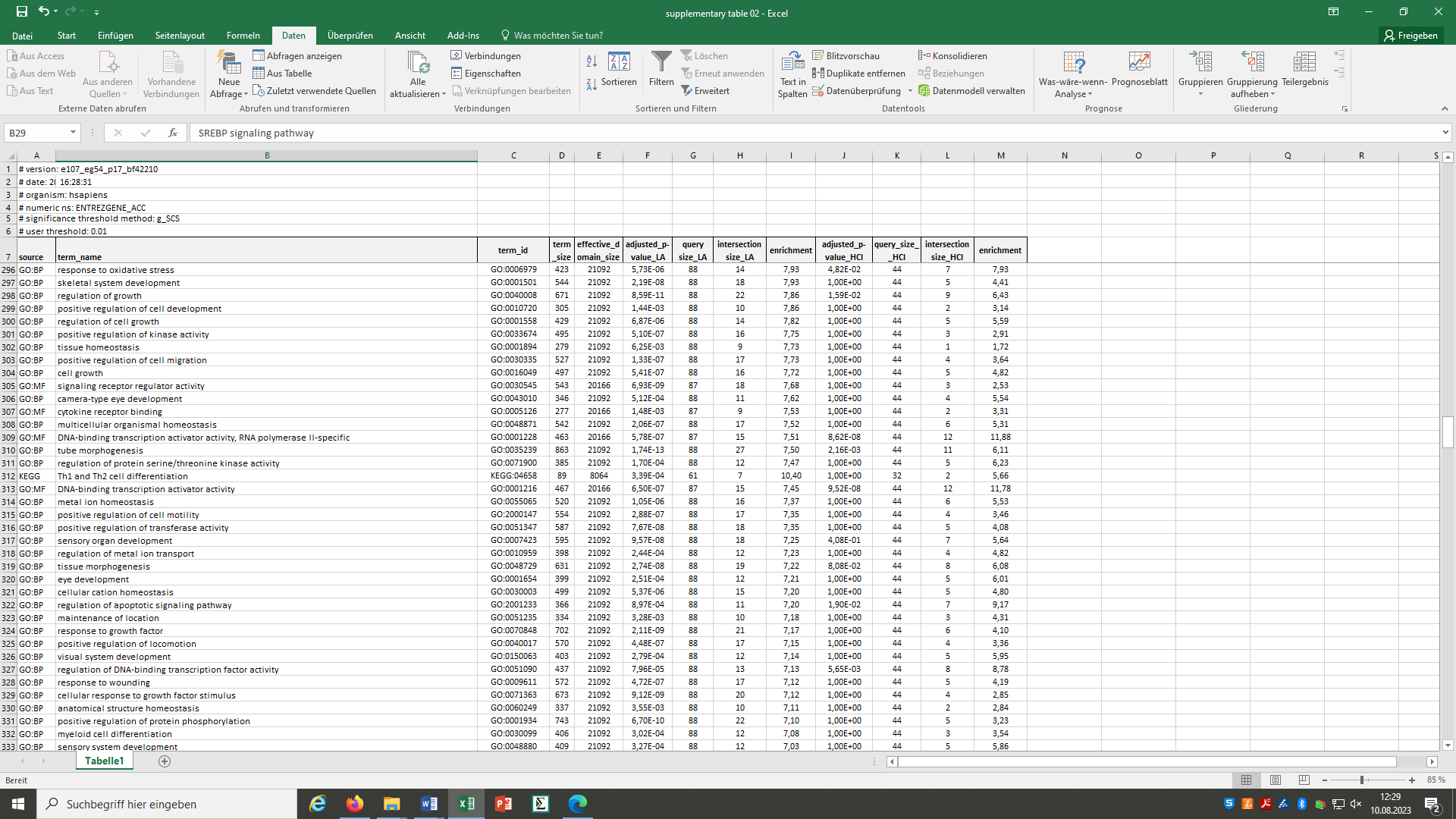

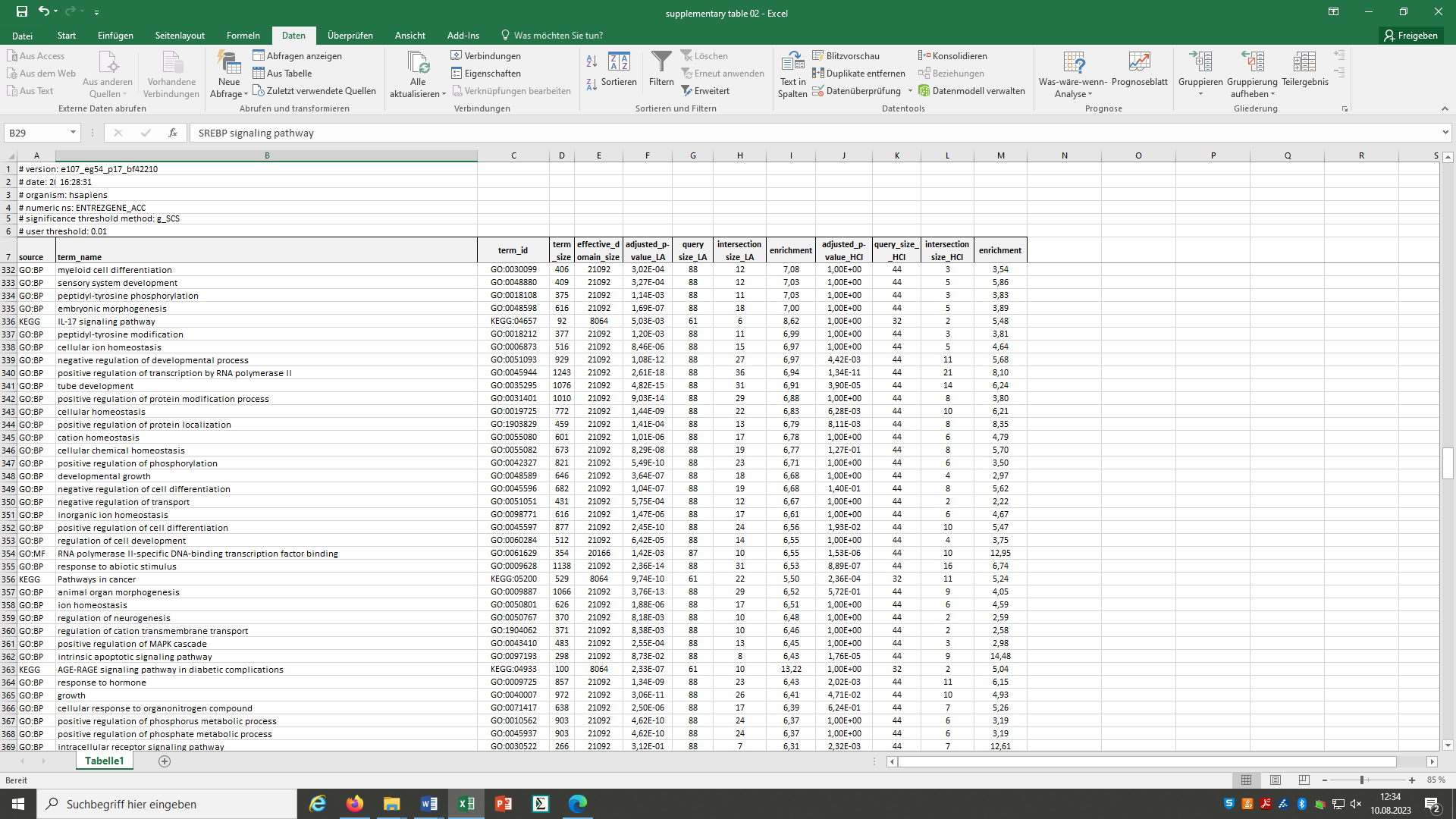

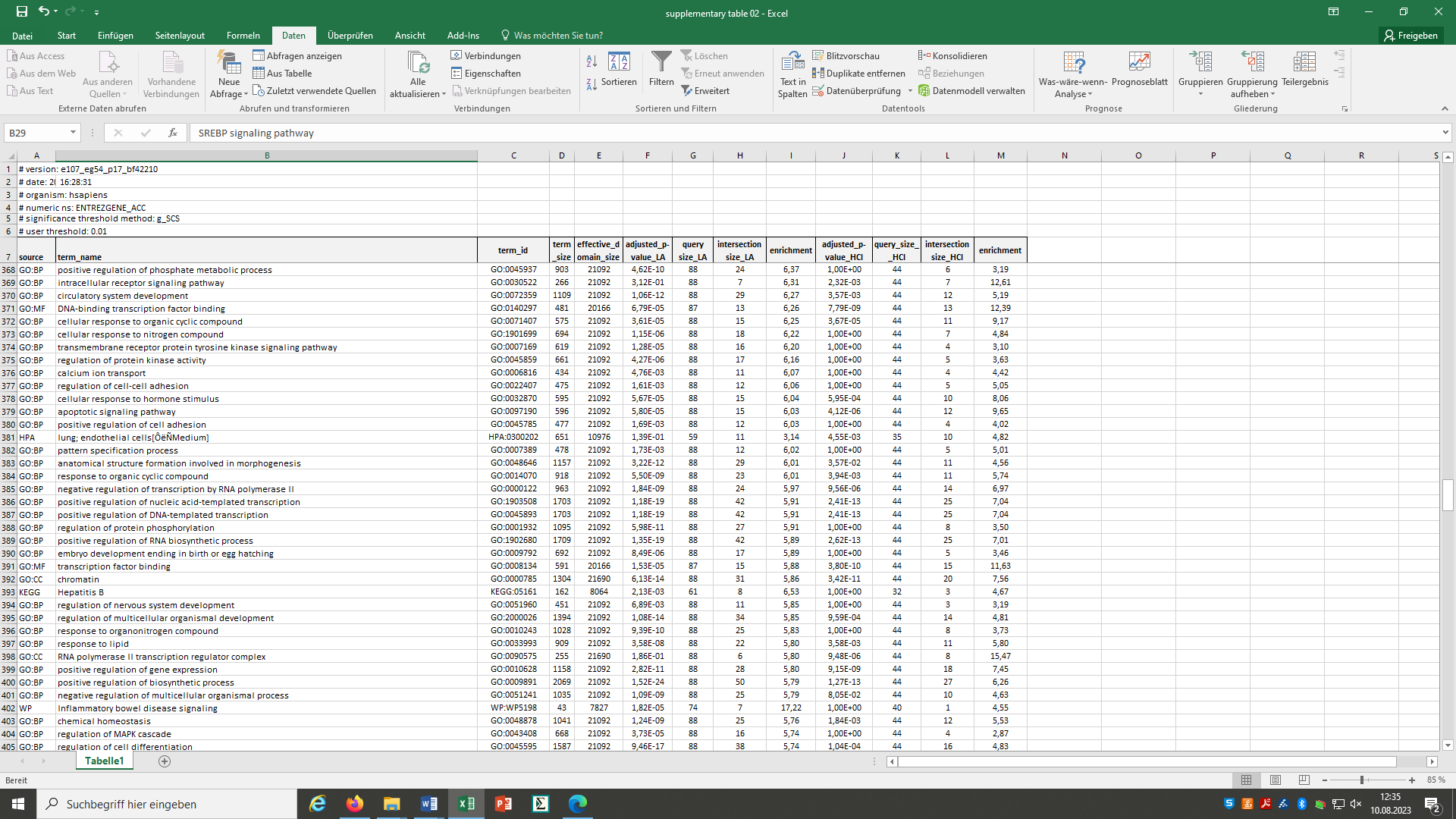

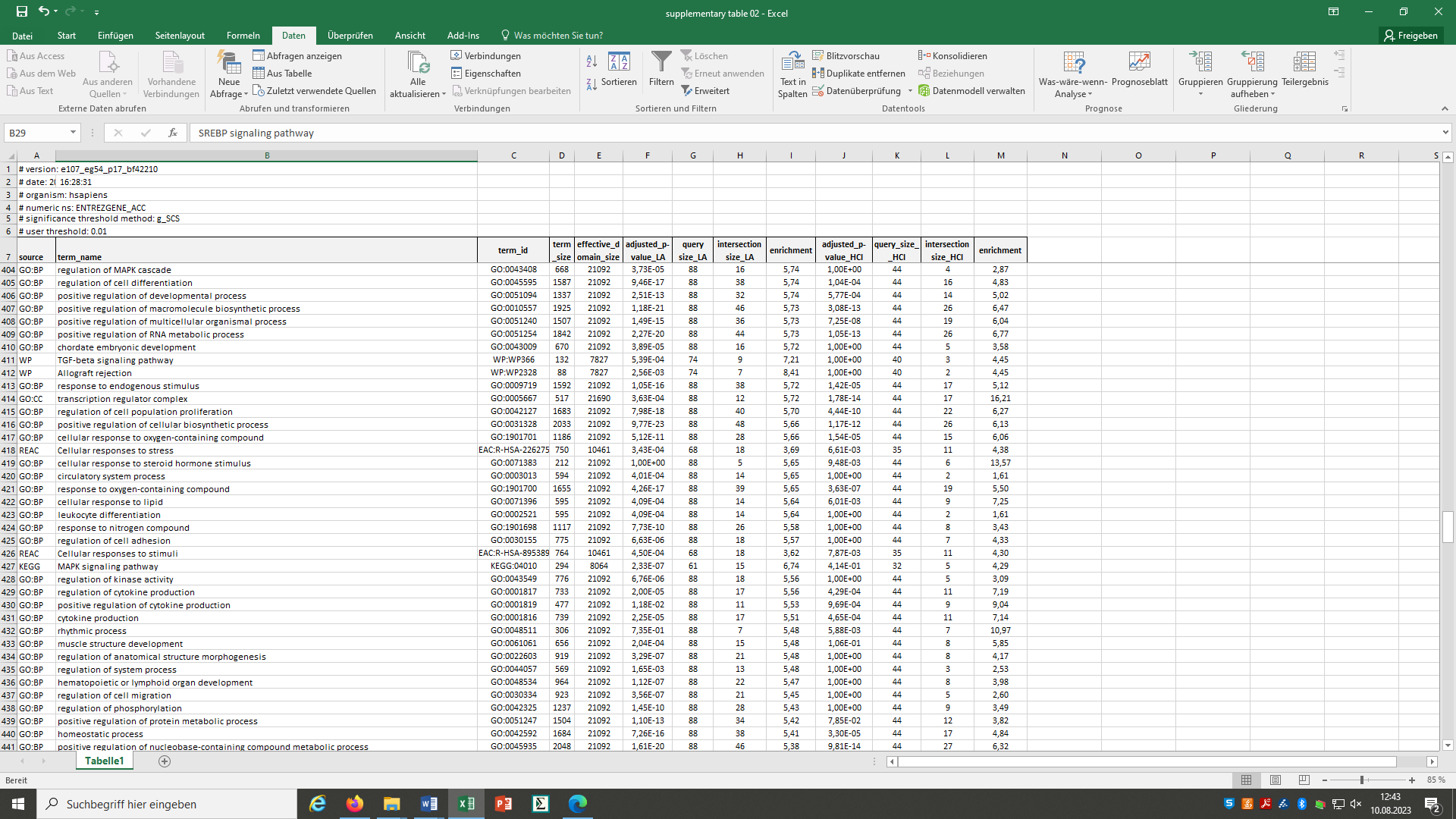


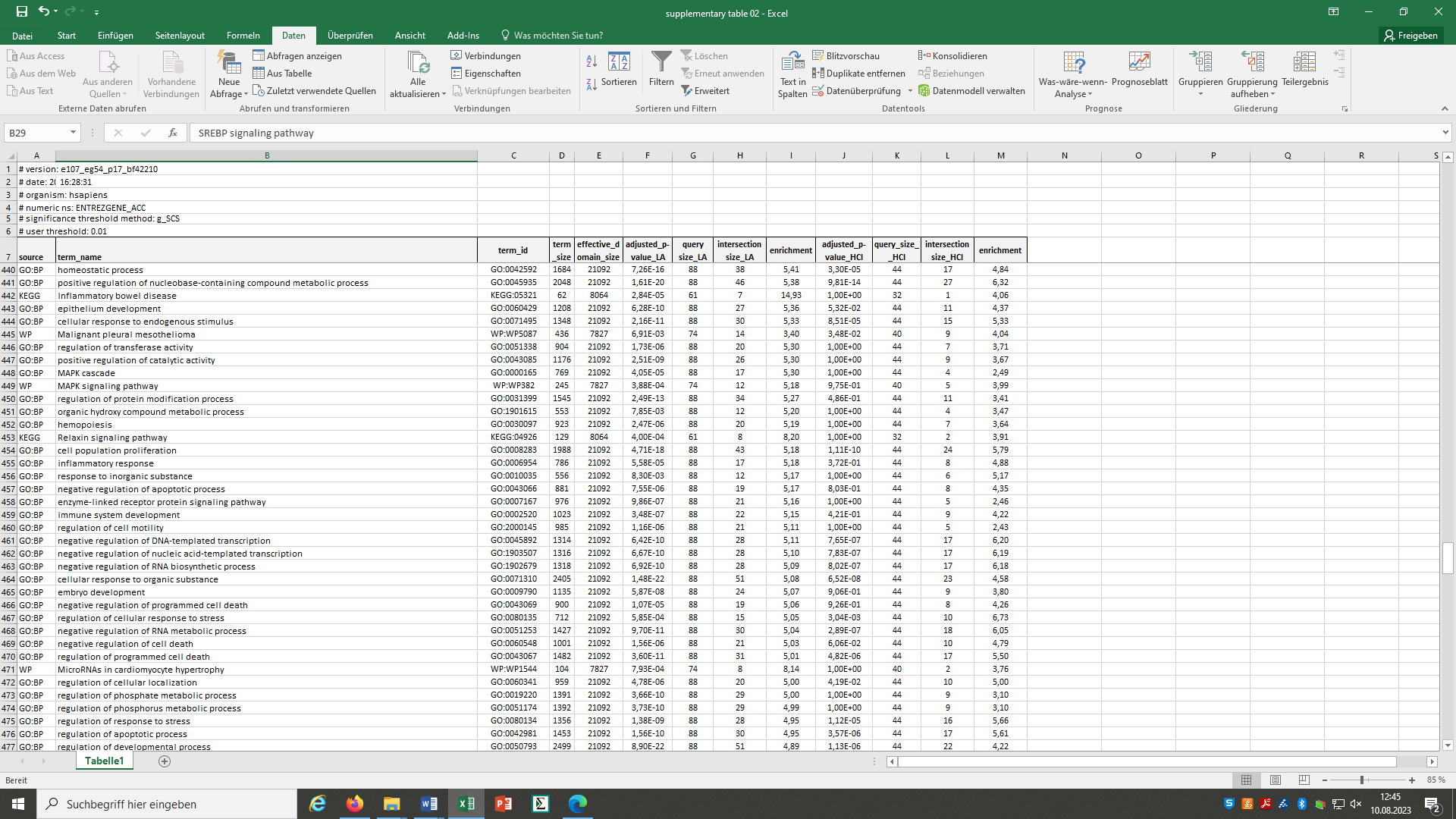

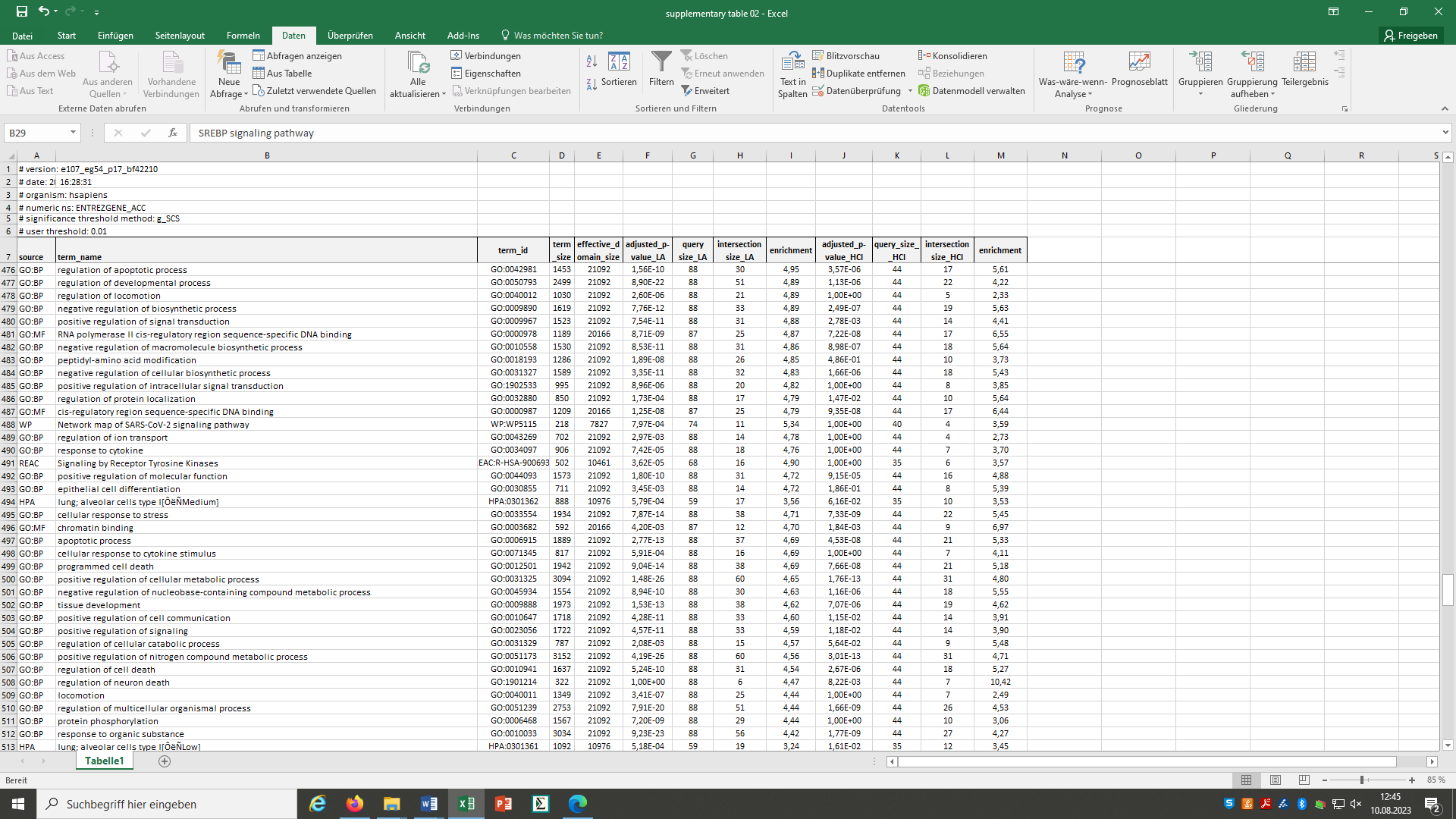

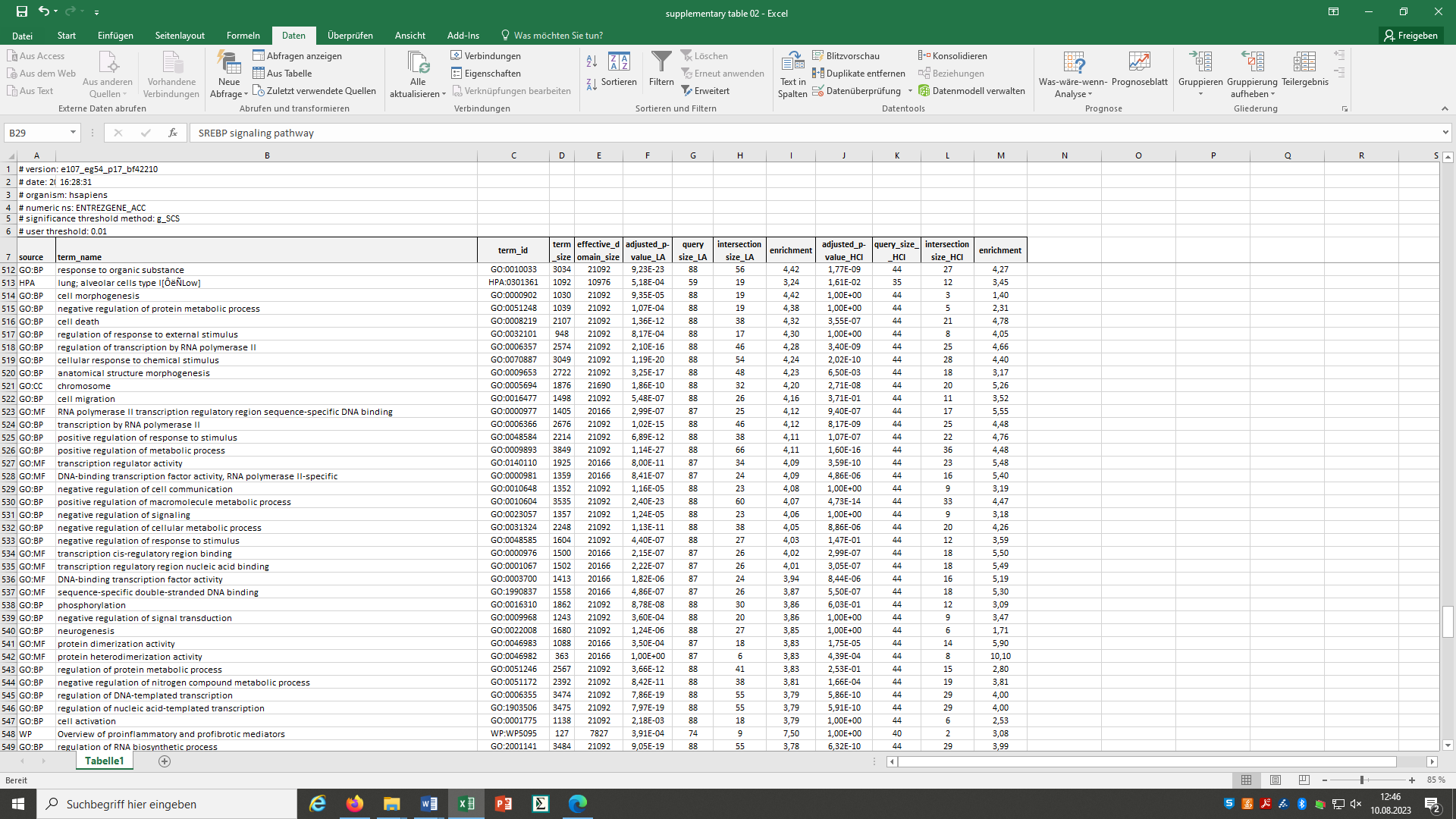

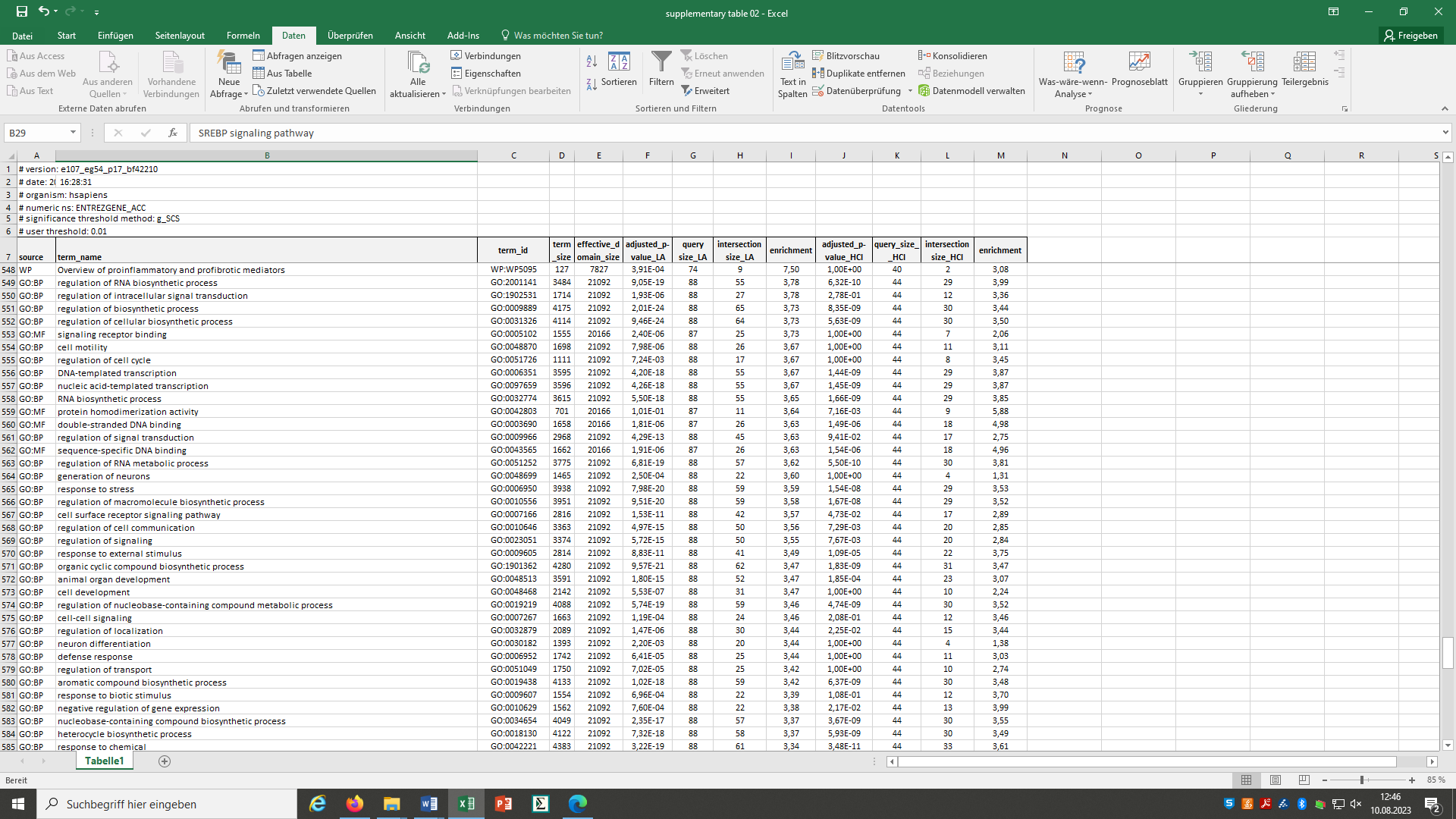

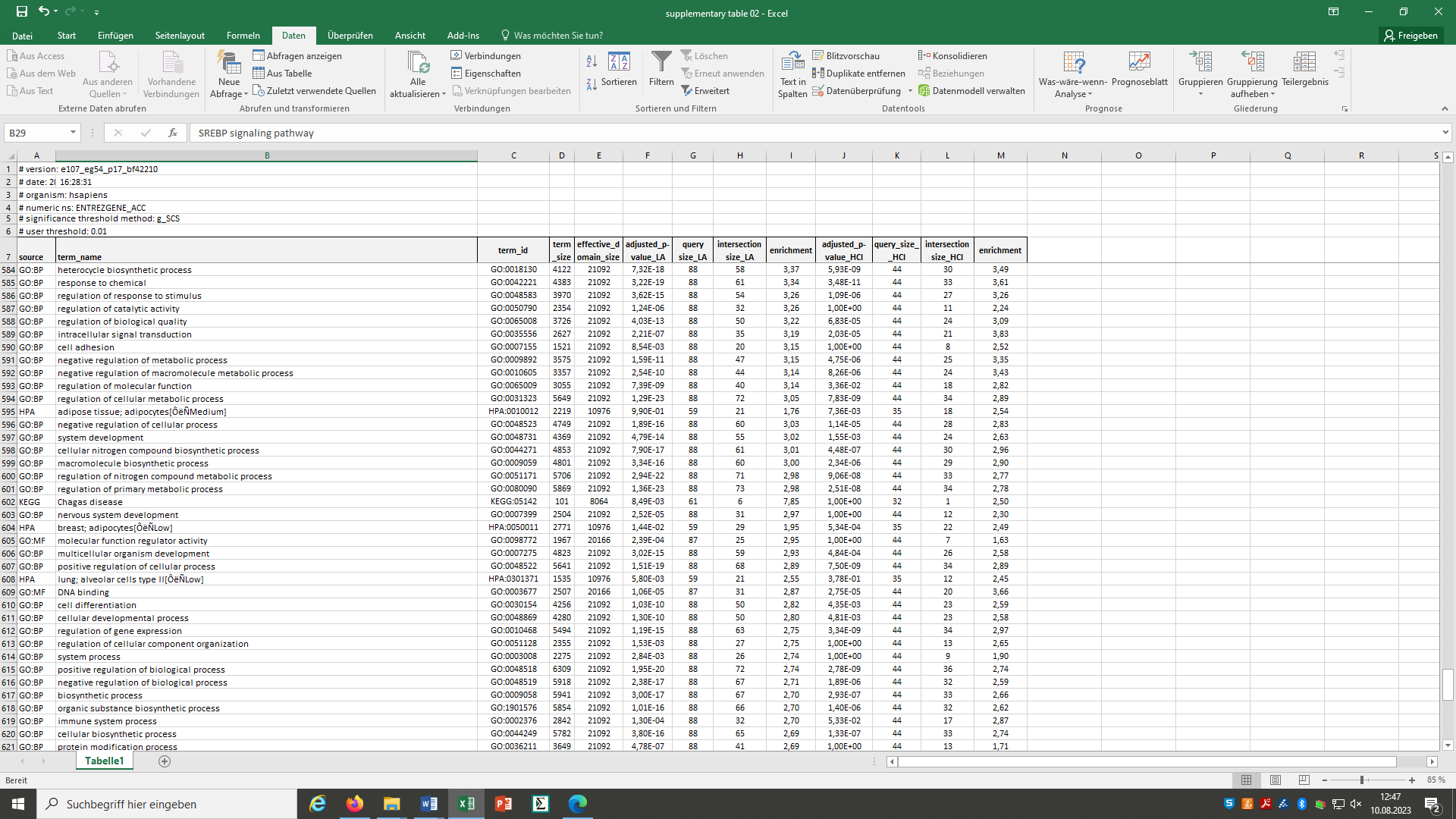

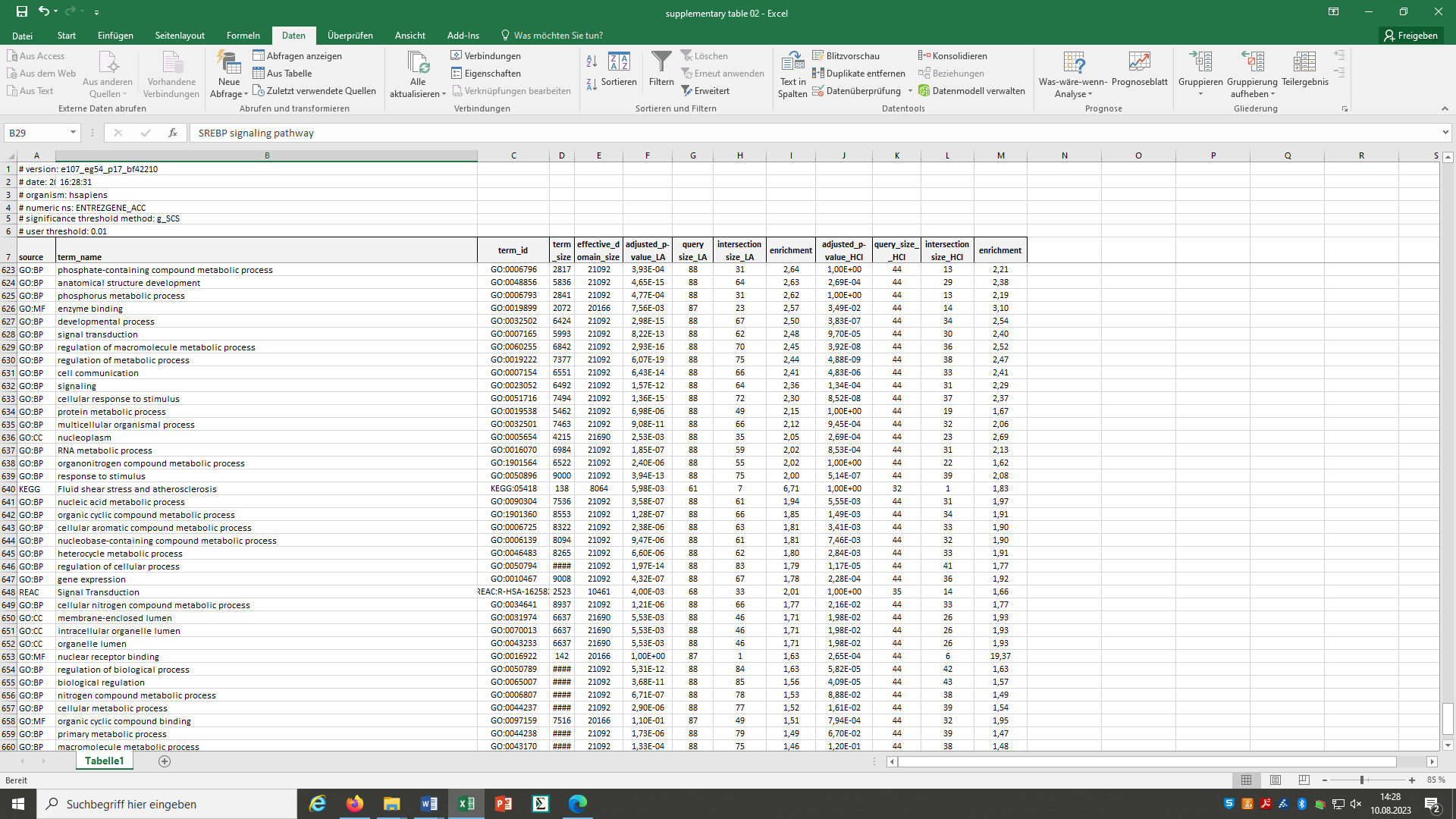

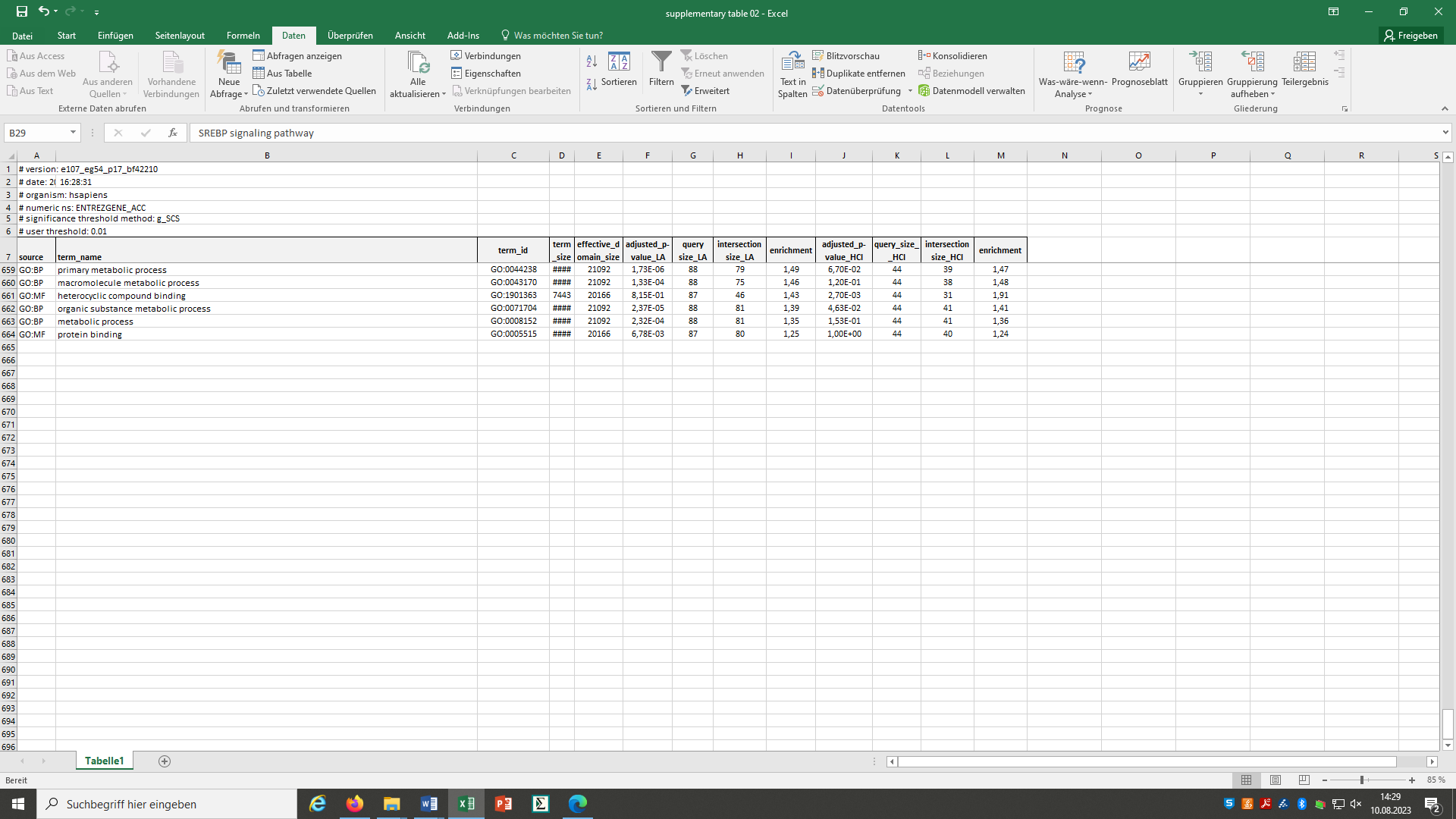


Shown are a g:Profler multiquery to compare the putative GO term enrichment of the two sets of predicted UR.

**Additional table S3**

Shown are the Disease and biofunction (DBF) analysis which were generated by IPA.

**Additiona table S4**

Shown are UR of the DEG_LA_-NOT-DEG_HCl_ set (lactacidosis-specific effect) by IPA.

**Additional table S5**


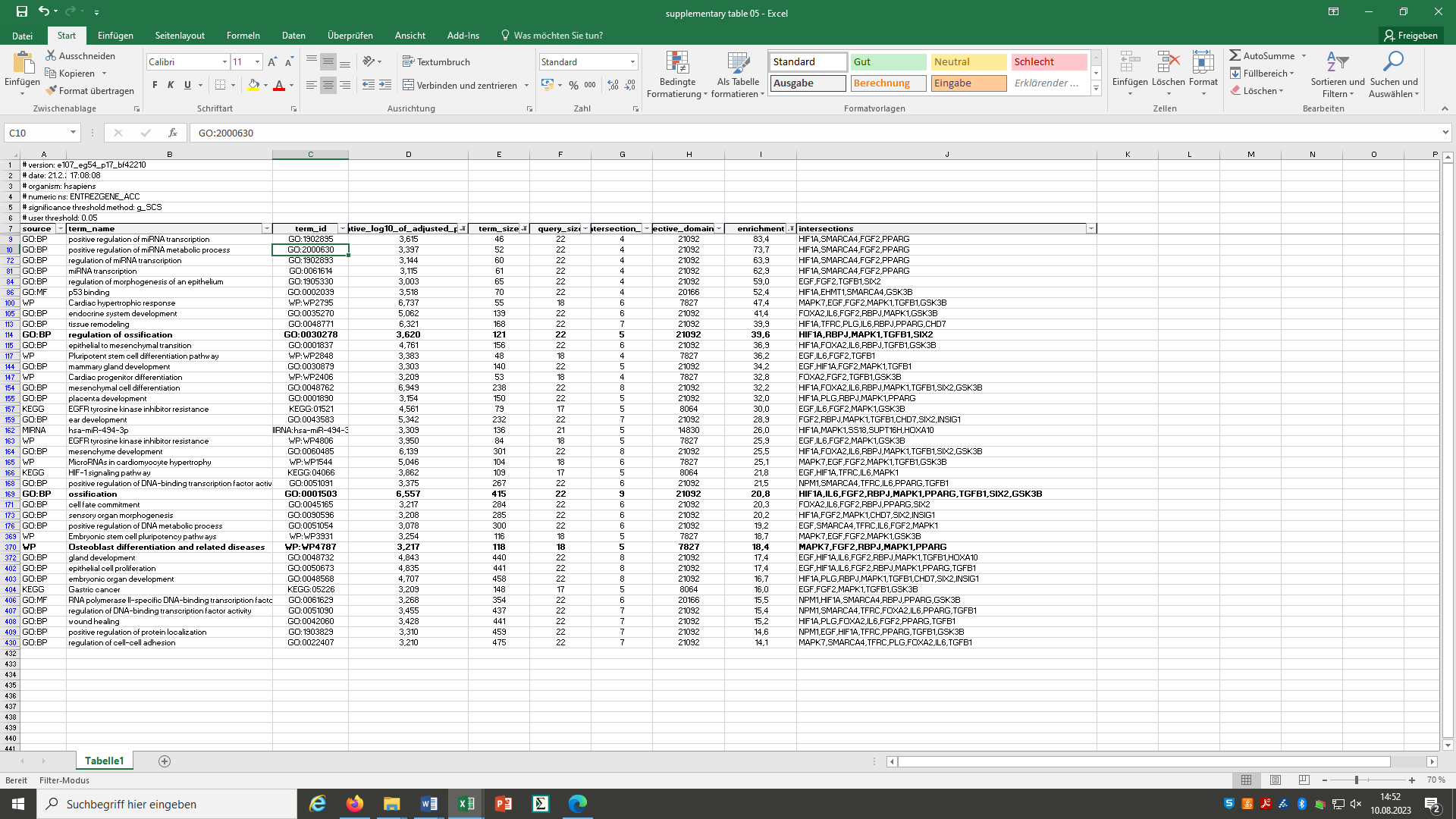


Shown are GO term enrichment analysis of the UR results.

**Additional Methods**

The following methods are described in more detail again:

## **Western Blot (part 2.7)**

HAoSMCs were lysed with RIPA buffer (150 mM NaCl, 10 mM TRIS base (pH 7.4), 1% NP-40 (v/v), 0.1% SDS (v/v), 1% Na-deoxycholate, 0.1% triton X-100 (v/v), 1 mM EDTA, 1 mM Na-orthovanadate, 1:500 protease inhibitor cocktail) and protein content was determined by BCA assay. 40 µg protein/lane was separated by 10% SDS-PAGE, transferred to a nitrocellulose membrane and incubated with primary antibodies (HIF-1α, #610959, Bd Transduction laboratories; TXNIP, #14715, cell signaling; GLUT-1, #12939, cell signaling; β-ACTIN, # 3700, cell signaling; VINCULIN, #13901, cell signaling) overnight at 4°C. The bound primary antibody was visualized by IRDye-secondary antibodies (Licor Biosciences, Lincoln, NE, USA) with the Odyssey imaging system (Licor Biosciences, Lincoln, NE, USA) or were detected in the linear range with the ECL system. Quantitative analysis was performed with Image Studio™ Lite quantification software (version 5.2, Licor Biosciences, Lincoln, NE, USA) or with Quantity One® (Biorad) fort he ECL system. β-actin, Vinculin and Ponceau S staining were used as reference for relative quantification.

## **ELISA for 5-Bromo-2’-deoxyuidine (BrdU) incooperation (part 2.8)**

HAoSMCs cultivated in a 96-well plate were incubated with BrdU (10 µM) and treated for 48 h. After washing, cell fixation (4% formaldehyde, 60 min, RT) and permeabilization, cellular DNA was denatured (2N HCl, 30 min, RT). After washing and incubation of the cells in permeabilization buffer (0.1% triton X-100 (v/v), 37 mg/L Na-orthovanadate in PBS) for 60 min and in blocking solution (0.1% triton X-100 (v/v), 10% FCS (v/v) in PBS buffer; 60 min), cells were incubated with anti-BrdU antibody (#347580, Becton Dickinson) overnight. After washing, a horseradish peroxidase-coupled secondary antibody was added and followed by a specific colored substrate reaction (0.5 mg/ml o-phenylenediamine, 11.8 mg/ml Na_2_HPO_4_ x 2H_2_O plus, 7.3 mg/ml citric acid and 0.015% H_2_O_2_) which was stopped with 1M H_2_SO_4_ and detected at 490 nm in a multiwall reader. For normalization cell densityF was determined by 0.2% Trypan Blue staining for 5 min at 37°C. After washing and incubation with 1% SDS (30 min, room temperature) the absorption was measured at 560 nm.

## **Lactat dehydrogenase (LDH) assay (part 2.11)**

Supernatants and lysates of HAoSMCs were incubated with LDH substrate buffer. Turnover of LDH substrate was measured at 334 nm (NADH) for 30 min at 37°C. Relative LDH release was calculated by correlation to the total cellular LDH content.

## **Determination of glucose consumption and lactate production (part 2.12)**

Glucose consumption determination was based on a two-step coupled involving hexokinase (catalyses the reaction: glucose + ATP 🡪 glucose-6-phosphate (G6P) + ADP) and glucose-6-phosphate dehydrogenase (G6PD, catalyses the reaction: G6P + NADP^+^ 🡪 6-phospho-gluconate + NADPH). Lactate production determination was based on the enzymatic reaction catalysed by lactate dehydrogenase (LDH): Lactate + NAD^+^ 🡪 pyruvate and NADH. NADPH/NADH generation was measured by an absorbance at 340 nm.

## **2.17 Determination of Oxygen Consumption Rate (OCR; part 2.17)**

The mitochondrial stress test was performed by measuring OCR under basal conditions following by the addition of inhibitors. First, oligomycin (2 µM) was added to inhibit complex V (ATP Synthase) of the electron transport chain (ETC). Then, FCCP (4 µM) was added to collapse the proton gradient and to disrupt the mitochondrial membrane potential. Finally, rotenone and antimycin A (0.5 µM) were injected to inhibit complexes I and III, respectively. During last injection, Hoechst 33342 (6 µM) was added to normalize the OCR to the cell number. The Hoechst 33342-induced nuclear staining was detected by a digital fluorescence microscopy (Cytation3, BioTek, Bad Friedrichshall, Germany). Hereby, the non-mitochondrial respiration (OCR_min_Rotenone/AntimycinA_), basal respiration rate (OCR_initial value_ - OCR_min_Rotenone/AntimycinA_), mitochondrial ATP production rate (OCR_initial value_ - OCR_min_Rotenone/AntimycinA_), proton leak (OCR_min Oligomycin_- OCR_min_Rotenone/AntimycinA_), maximal respiration (OCR_max.FCCP_ - OCR_min_Rotenone/AntimycinA_), spare respiratory capacity (maximal respiration – basal respiration), coupling efficiency ((ATP production rate/basal respiration rate) x 100)) were calculated as indicated.

## **Determination of intracellular pH (pH_i_; part 2.17)**

The pH_i_ of single cells was determined by the pH-sensitive fluorophore BCECF-AM (5 µM) which was added to the priming medium for 15 min. After washing once with Ringer solution (adjusted to the corresponding pH) the coverslip was transferred to an inverted Axiovert 100 TV microscope (Zeiss, Oberkochen, Germany) and regions of interests (ROIs) were drawn for individual cells. Under permanent perfusion with Ringer solution (adjusted to the corresponding pH) the BCECF fluorescence intensity ratio was determined by excitation at 495 nm and 440 nm and the emitted light was filtered through a bandpass-filter (515–565 nm) every 10s. To investigate acute changes of pH_i_ the fluorescence intensity ratio was measured after cells were flooded first with control Ringer solution and then with acidic Ringer solution. pH calibration was performed after each experiment using Calibration solution at pH 6.6 and pH 7.6 consisting of a high K^+^-content and the ionophore nigericin (10 µM). Background signals were corrected by subtracting the ratio of a blank ROI from all sample ratios.

The following table summarizes the used buffers:

| **Name** | **Composition** |
| --- | --- |
| ALP reaction buffer | 10 mM p-nitrophenyl phosphate disodium hexahydrate, 1 M diethanolamine, 0.1 mM ZnCl_2_, pH 9.8 HCl |
| Blocking solution | in PBS buffer: 0.1% triton X-100 (v/v), 10% FCS (v/v) |
| Calibration solution | 132 mM KCl,1 mM CaCl2, 1 mM MgCl2,10 mM HEPES, 10 µM nigericin |
| Caspase lysis buffer | 10 mM TRIS, 100 mM NaCl, 1 mM EDTA,  0.01% triton X-100 (v/v), pH 7.5 |
| Caspase reaction buffer | 20 mM piperazine-N,N′-bis(2-ethanesulfonic acid), 4 mM EDTA, 0.2% 3-[(3-cholamidopropyl)dimethylammonio]-1-propanesulfonate (w/v), 10 mM dithiothreitol, pH 7.4 |
| ENPP1 reaction buffer | 1 mg/ml p-nitrophenyl thymidine 5'-monophosphate, 200 mM TRIS base, 0.1 mM ZnCl_2_, pH 8.0 HCl |
| Glucose reaction buffer | in TEA buffer: 4.4 mM ATP, 0.8 mM NADP sodium salt hydrate, 33.04 mU/ml Hexokinase + G6PD |
| HEPES-Ringer buffer | 122.5 mM NaCl, 5.4 mM KCl, 0.8 mM MgCl_2_x6H_2_O, 1.2 mM CaCl_2_x2H_2_O, 1 mM NaH_2_PO_4_xH_2_O, 10 mM HEPES, 5.5 mM D-glucose, pH 7.4 |
| HRP substrate | 11.8 mg/mL sodium phosphate dibasic dehydrate, 7.3 mg/mL citric acid, 0.5 mg/mL o-phenylenediamine, 0.015% H_2_O_2_ (v/v) |
| Hydrazine-glycine buffer | 400 mM hydrazine, 500 mM glycine, pH 9.0 |
| Lactate reaction buffer | in hydrazine-glycine buffer: 4.384 mM NAD hydrate, 68.5 µg/ml LDH |
| 6x Redmix buffer | in 0.5 M TRIS-HCl pH 6.8: 10% SDS (w/v), 30% glycerol (v/v), 0.012% bromophenol blue (w/v), 6% mercaptoethanol (v/v) |
| LDH substrate buffer | in HEPES-Ringer buffer: 0.2 mM NAD hydrate, 1 mM pyruvate |
| MOPS-Triton lysis buffer | 20 mM 3-(N-morpholino)propanesulfonic acid,  0.1% triton X-100 (v/v), pH 7.4 NaOH |
| PBS buffer | 13.7 mM NaCl, 0.27 mM KCl, 0.81 mM Na_2_HPO_4_x2H_2_O, 0.15 mM KH_2_PO_4_, pH 7.4 NaOH |
| Permeabilization buffer | in PBS buffer: 0.1% triton X-100 (v/v), 37 mg/L Na-orthovanadate |
| Ringer solution | 24 mM NaHCO_3_, 0.8 mM Na_2_HPO_4_x2H_2_O, 0.2 mM NaH_2_PO_4_x2H_2_O, 86.5 mM NaCl, 5.4 mM KCl, 1.2 mM CaCl_2_x2H_2_O, 0.8 mM MgCl_2_, 20 mM HEPES,  11 mM D-glucose, pH 7.4 (NaOH) |
| RIPA buffer | 150 mM NaCl, 10 mM TRIS base (pH 7.4), 1% NP-40 (v/v), 0.1% SDS (v/v), 1% Na-deoxycholate, 0.1% triton X-100 (v/v), 1 mM EDTA, 1 mM Na-orthovanadate, 1:500 protease inhibitor cocktail |
| TBS buffer | 17 mM TRIS-HCl, 3 mM TRIS base, 140 mM NaCl, pH 7.4 HCl |
| TBS-Tween | in TBS buffer: 0.1% tween-20 (v/v) |
| TEA buffer | 250 mM triethanolamine hydrochloride, 2.5 mM MgCl_2_x7H_2_O |

Table 1: buffer compositions

**In this part the original Western Blot pictures are summarized:**

Western Blot GLUT-1 protein expression –Figure 4C:

**
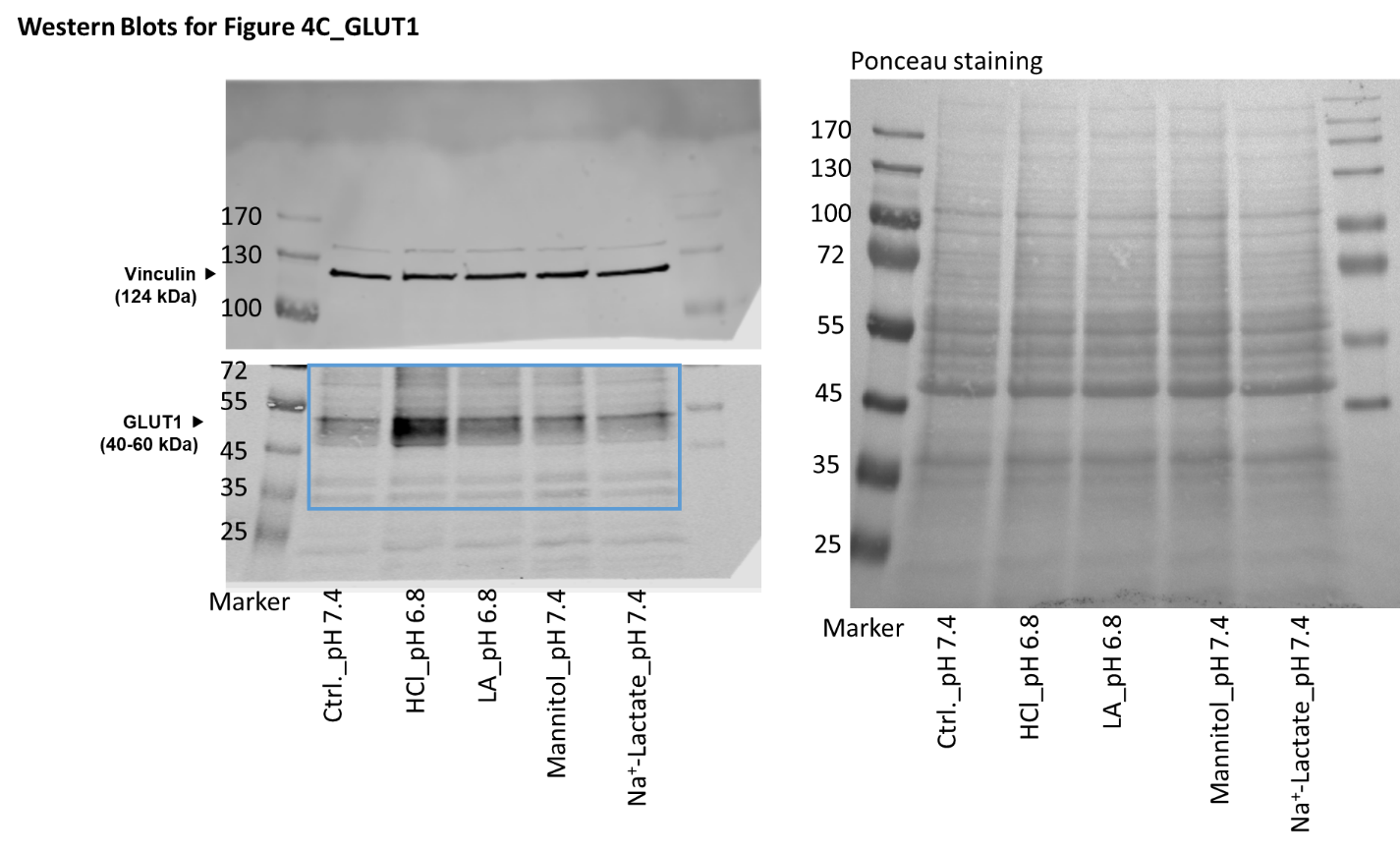
**


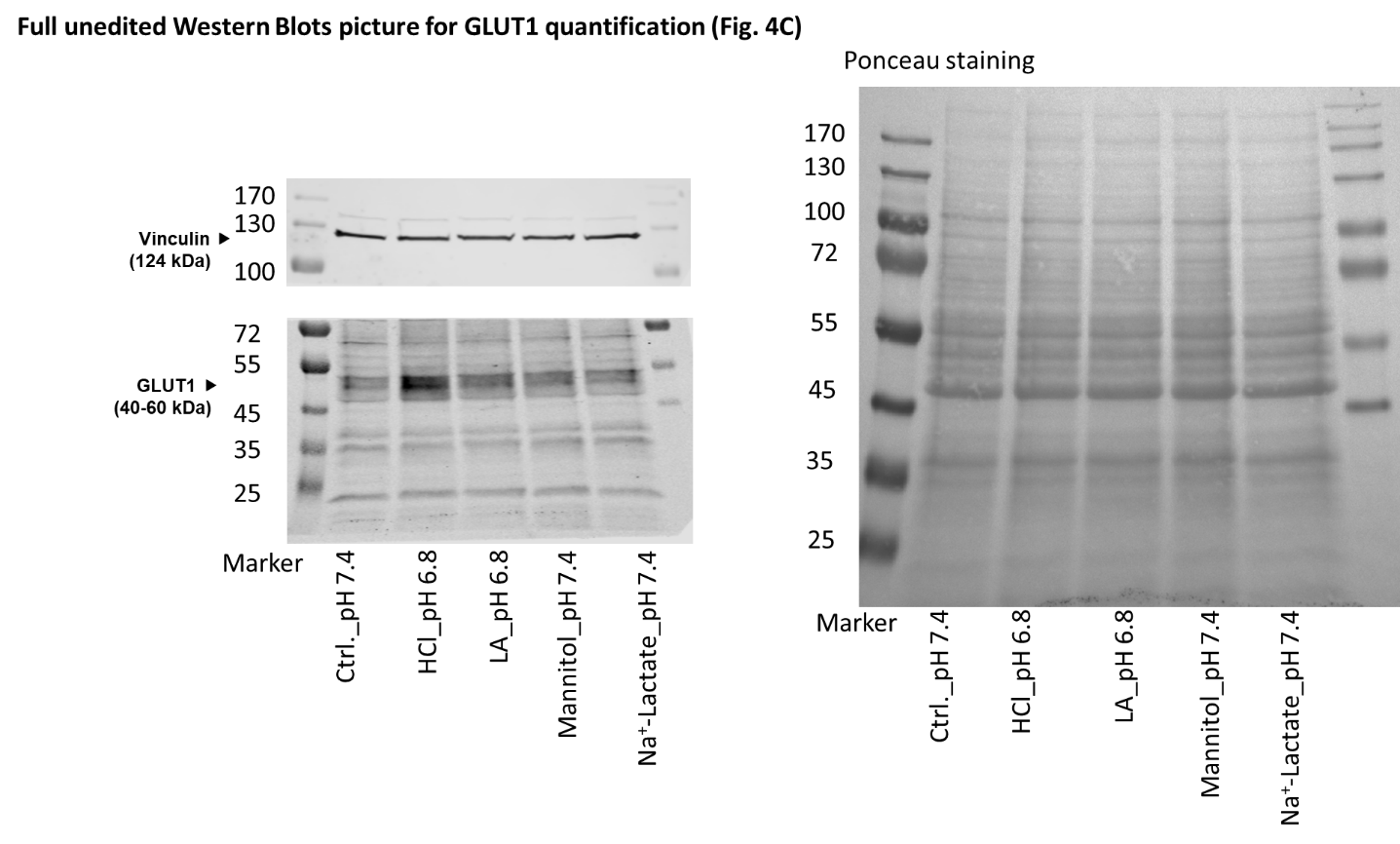


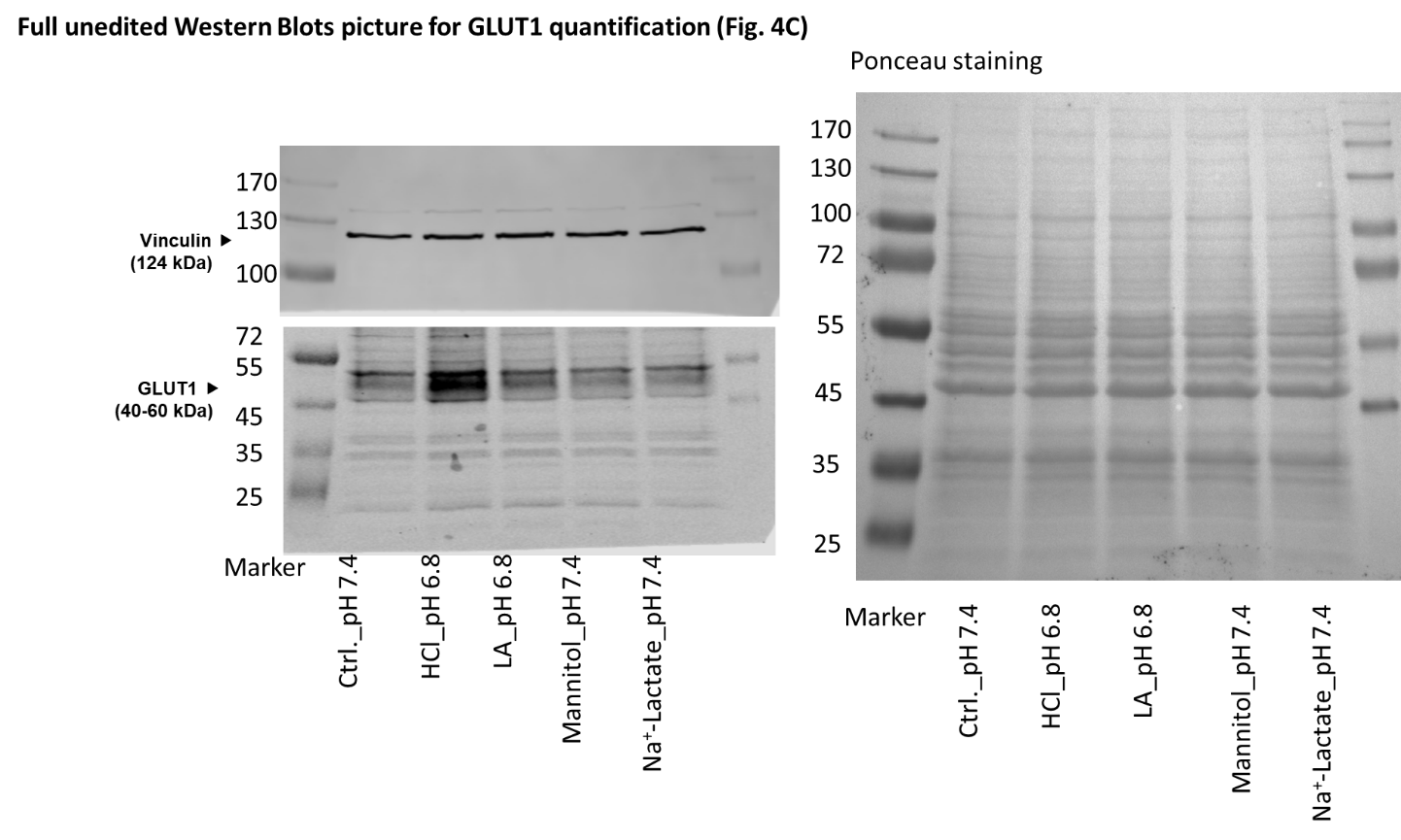


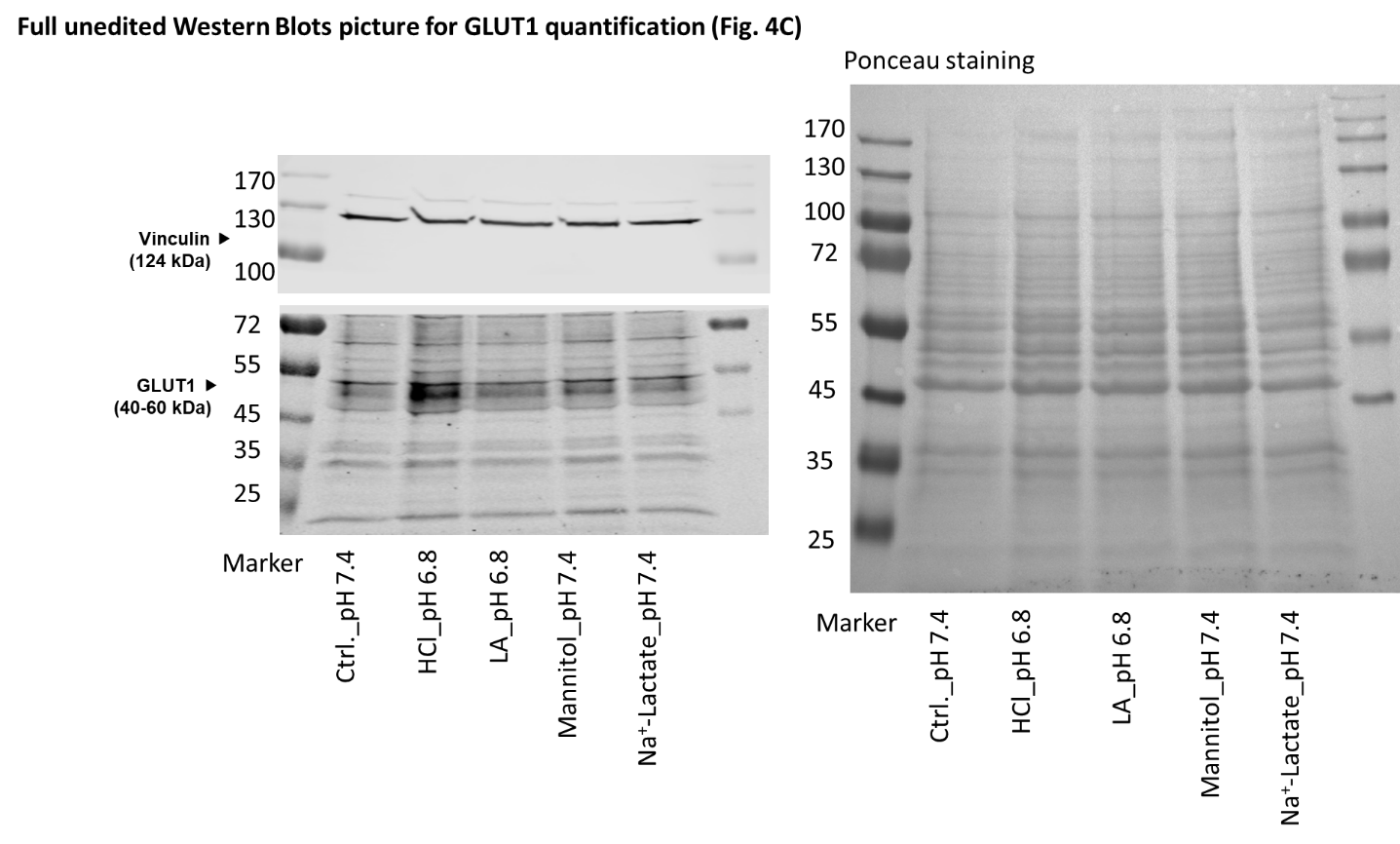


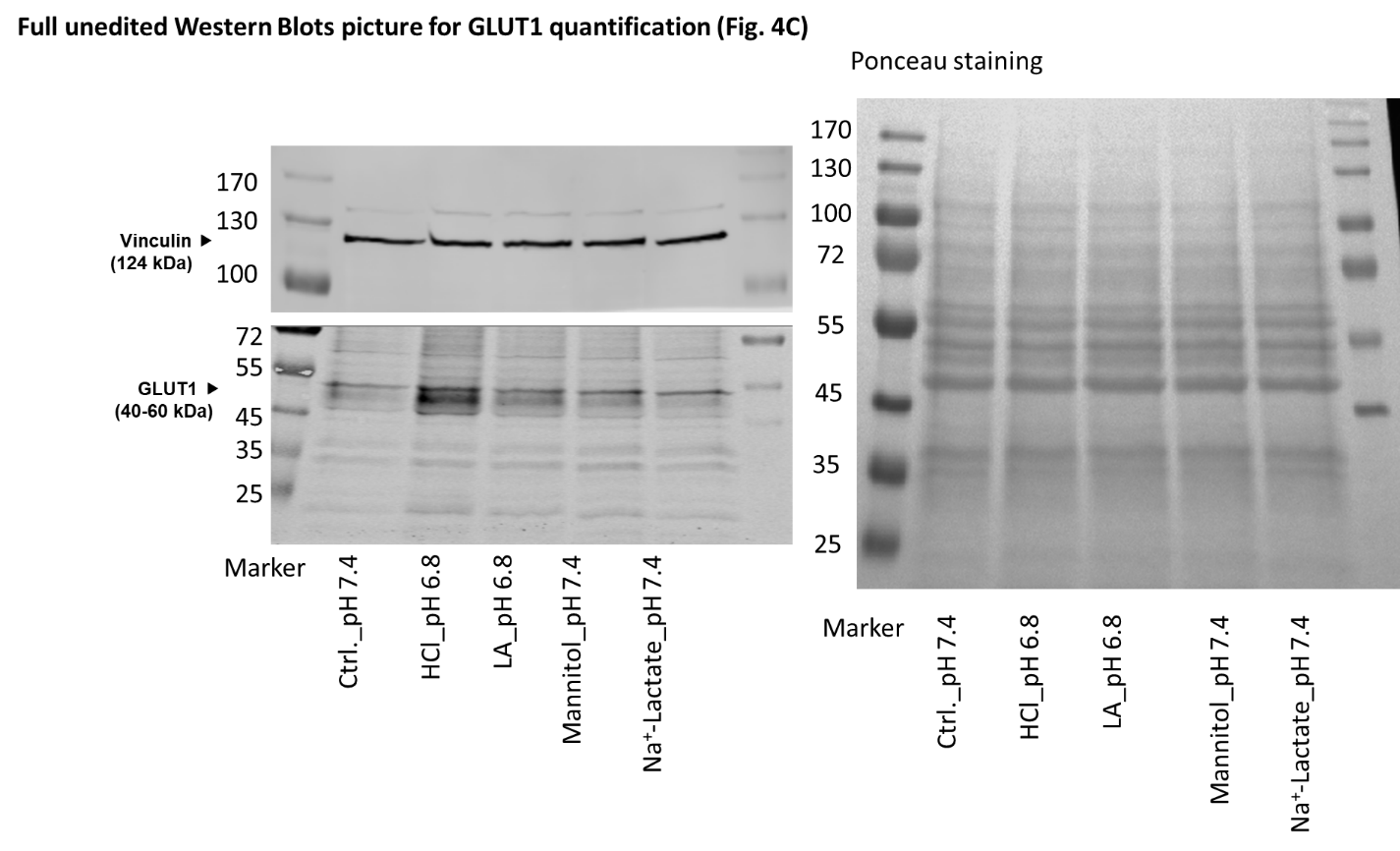


Western Blot TXNIP protein expression –Figure 4D:


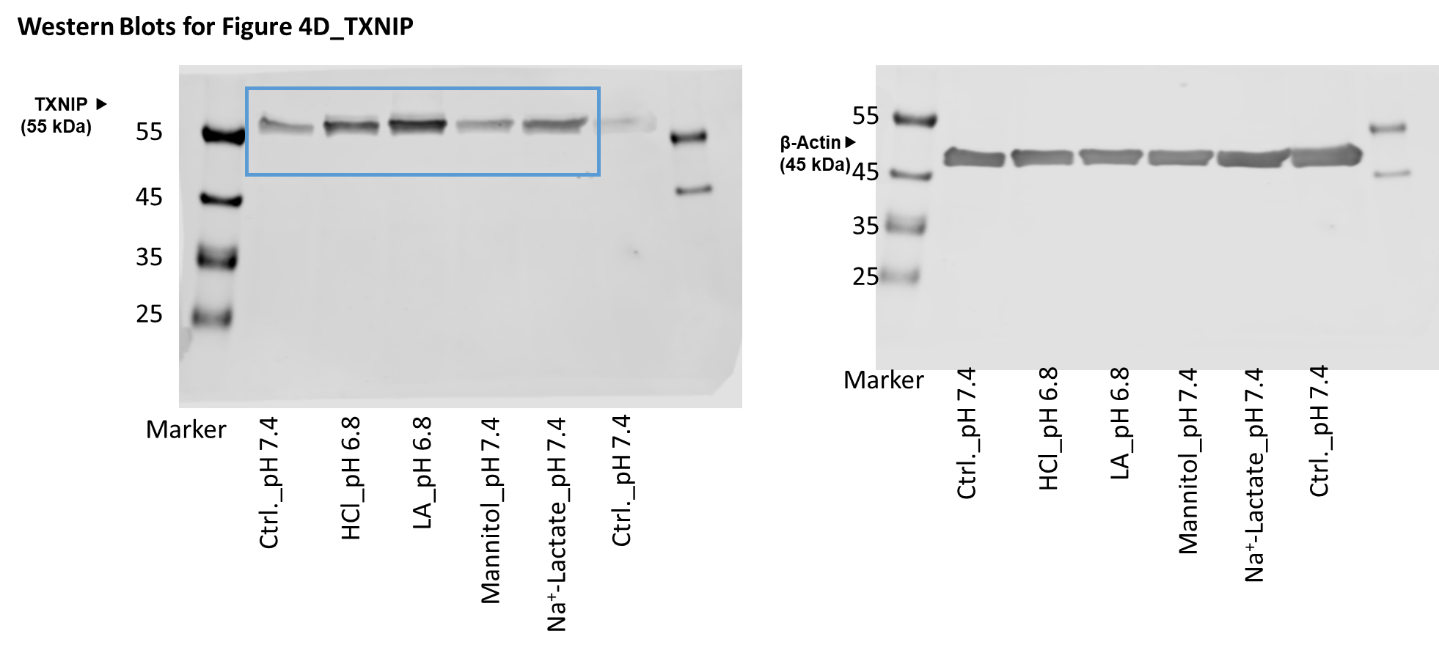


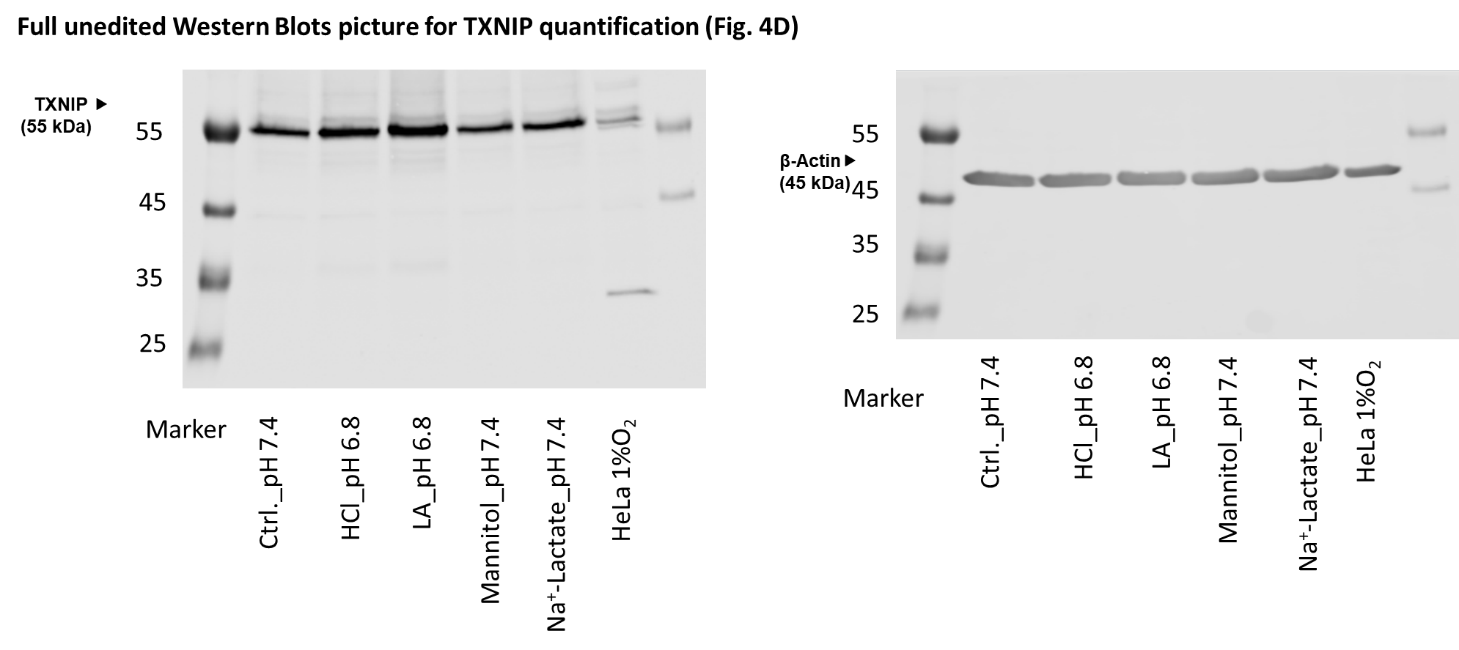


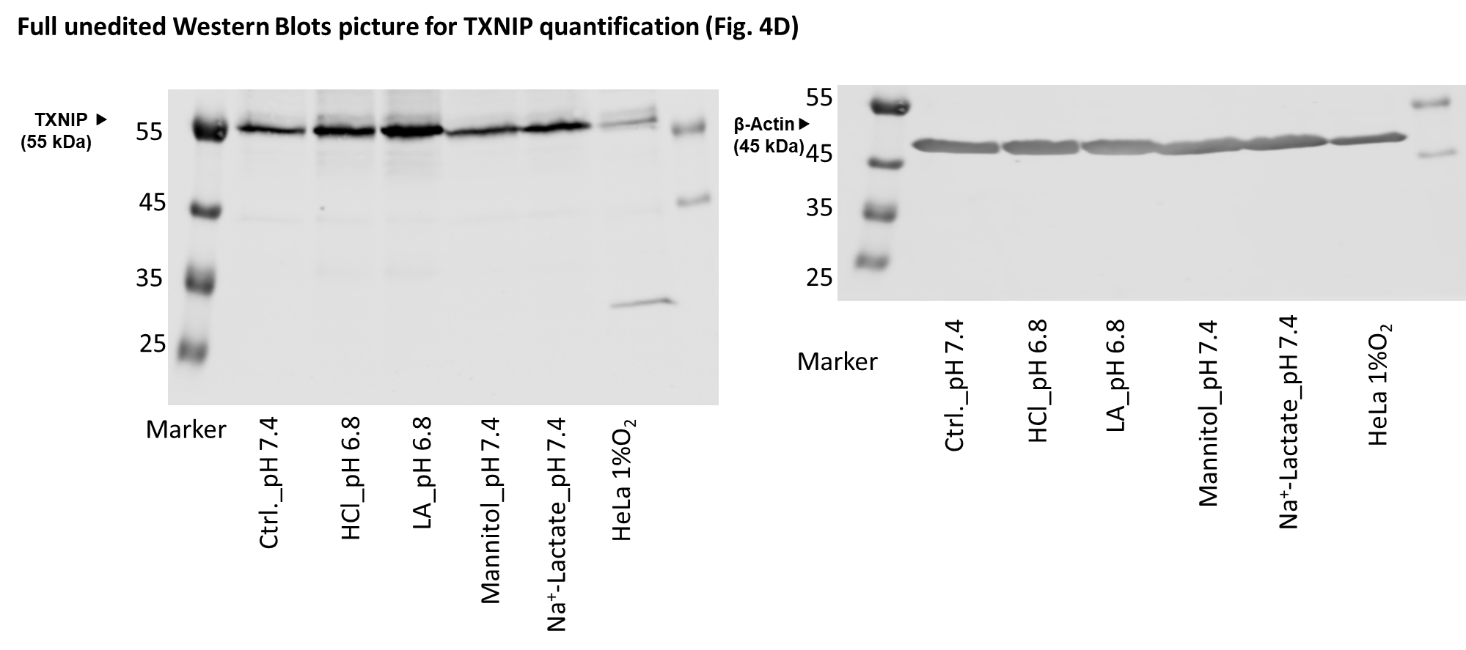


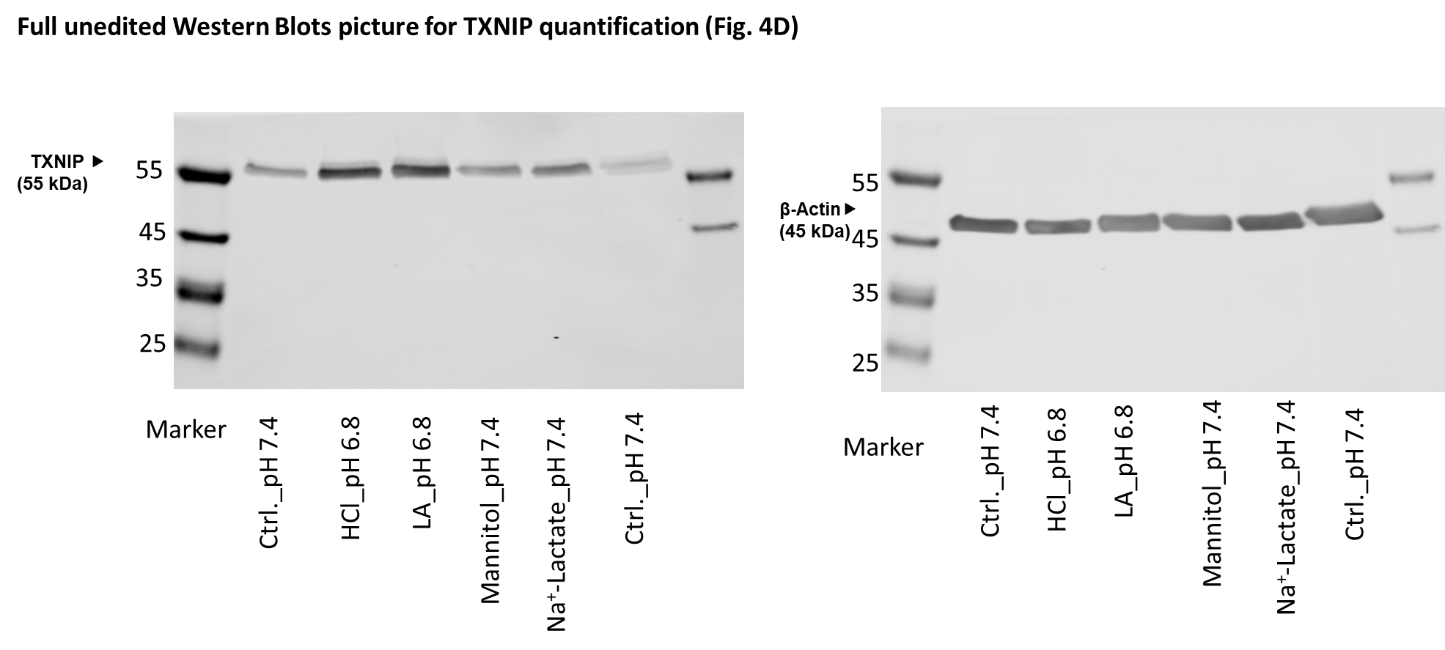


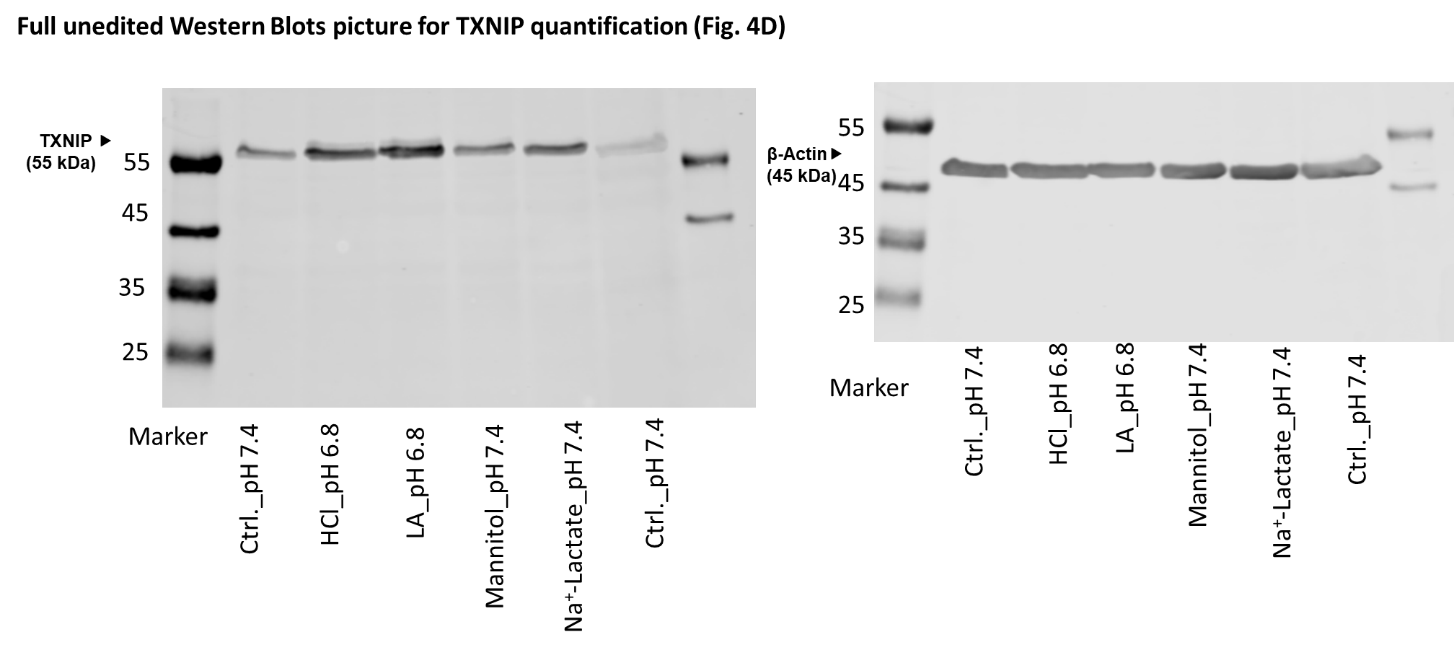


Western Blot HIF1α protein expression –Figure 4D:


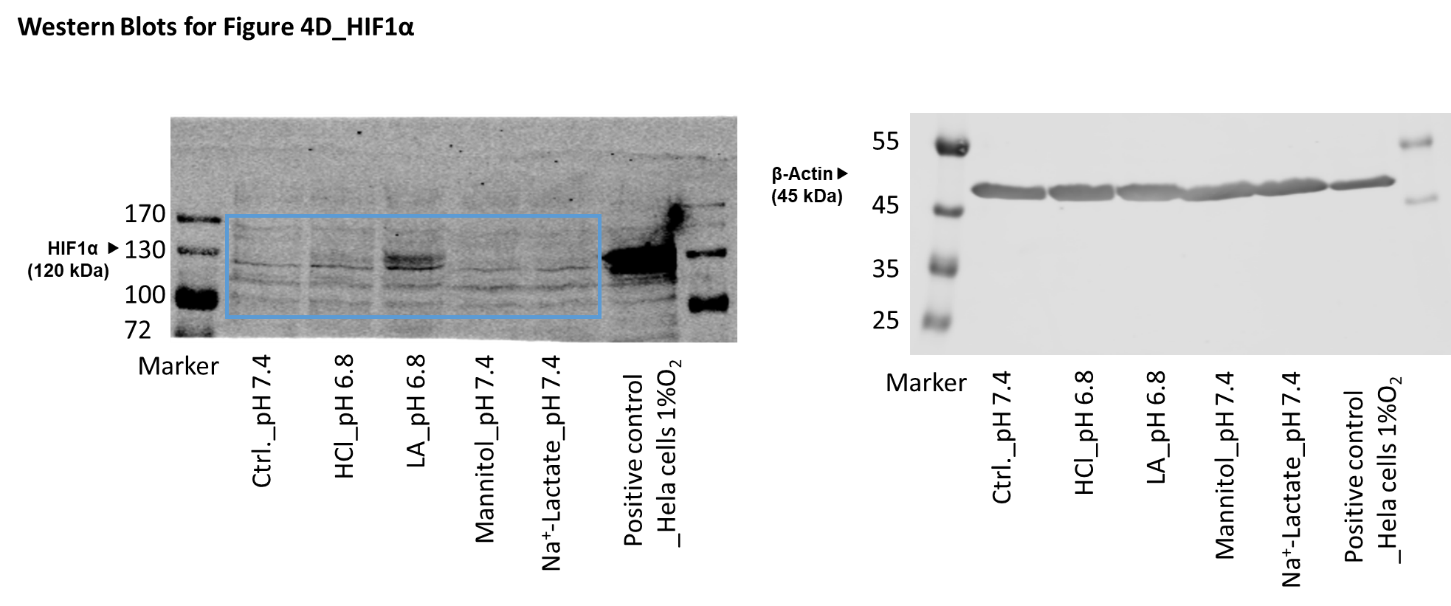


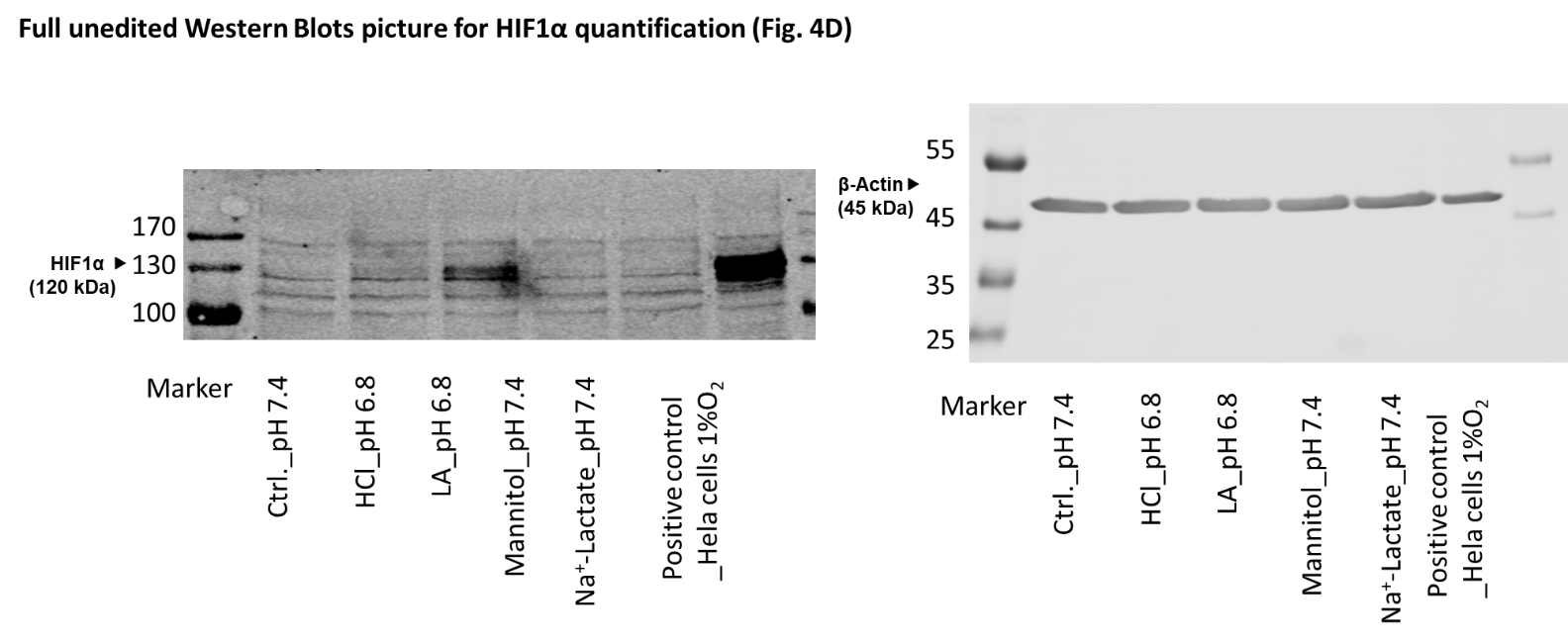


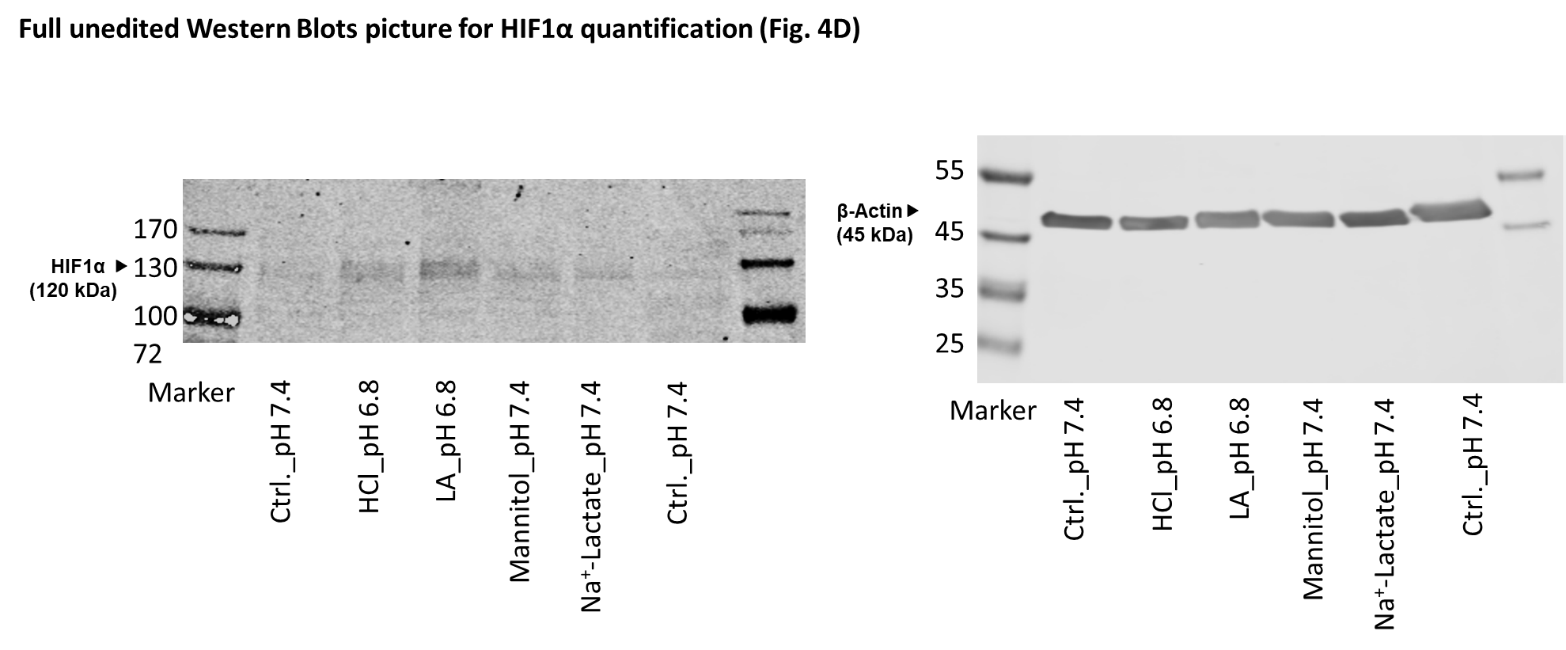


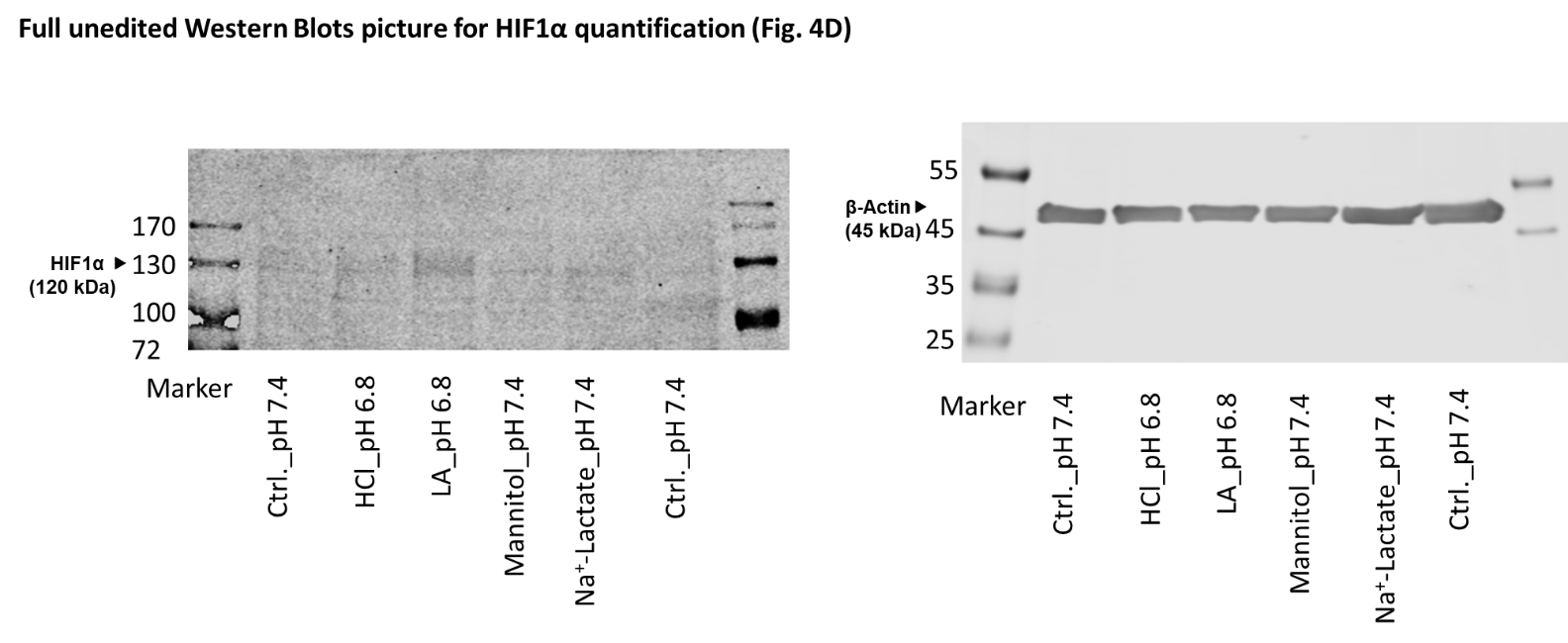


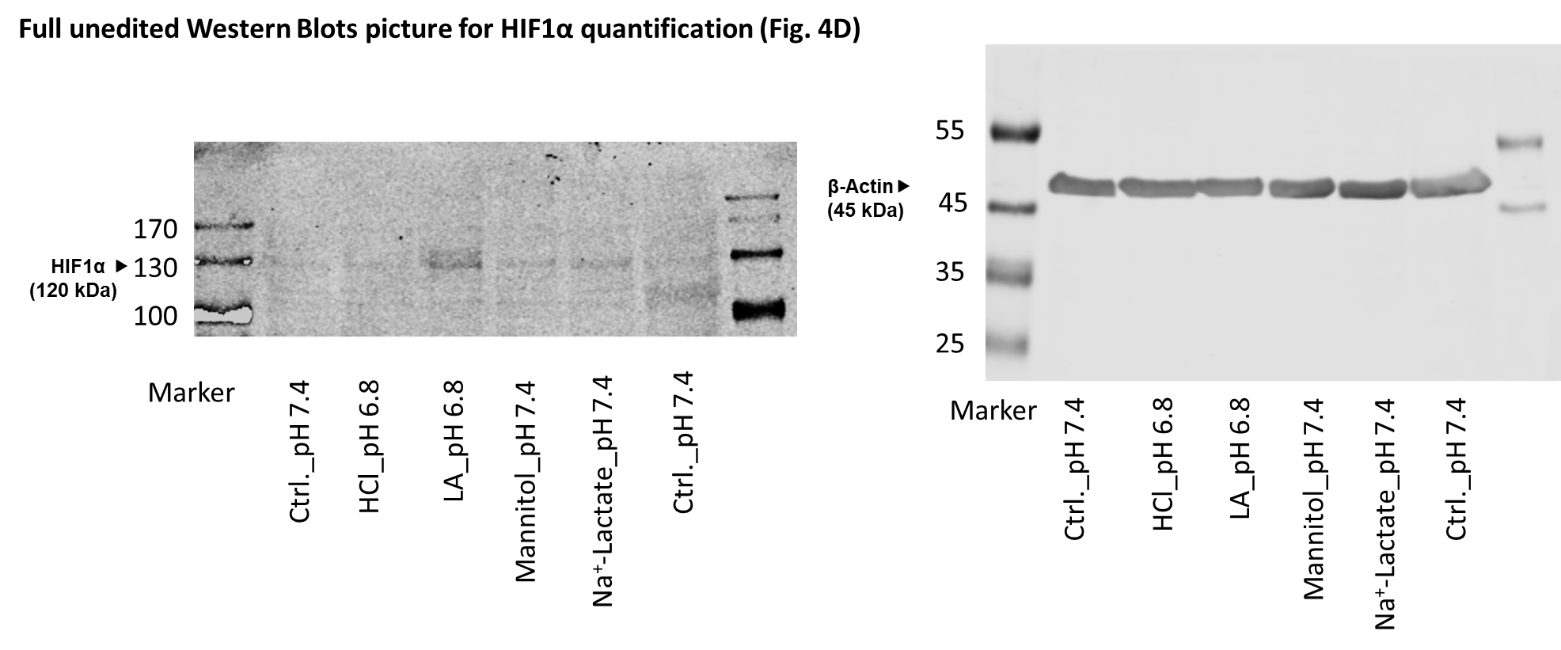


Western Blot c-MYC protein expression –Figure 4D:


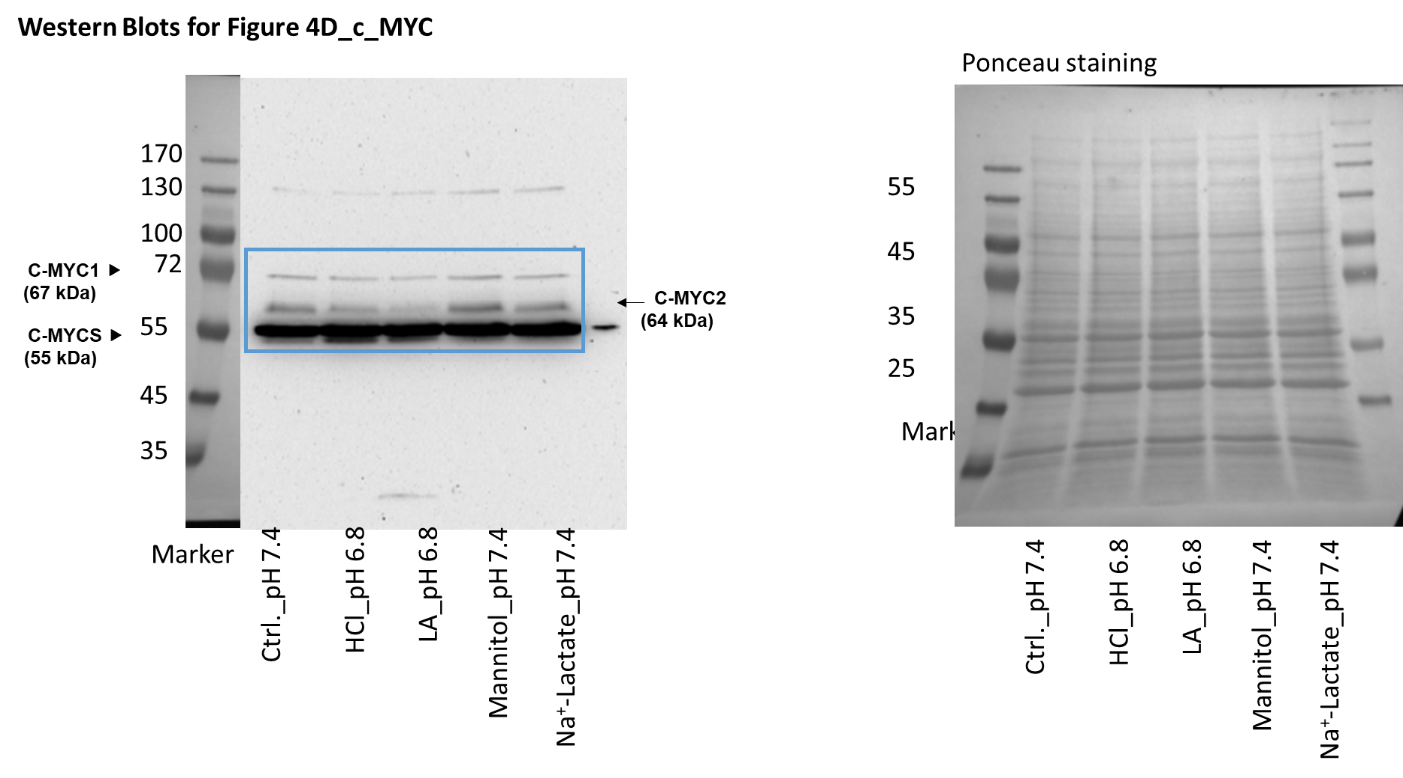


**
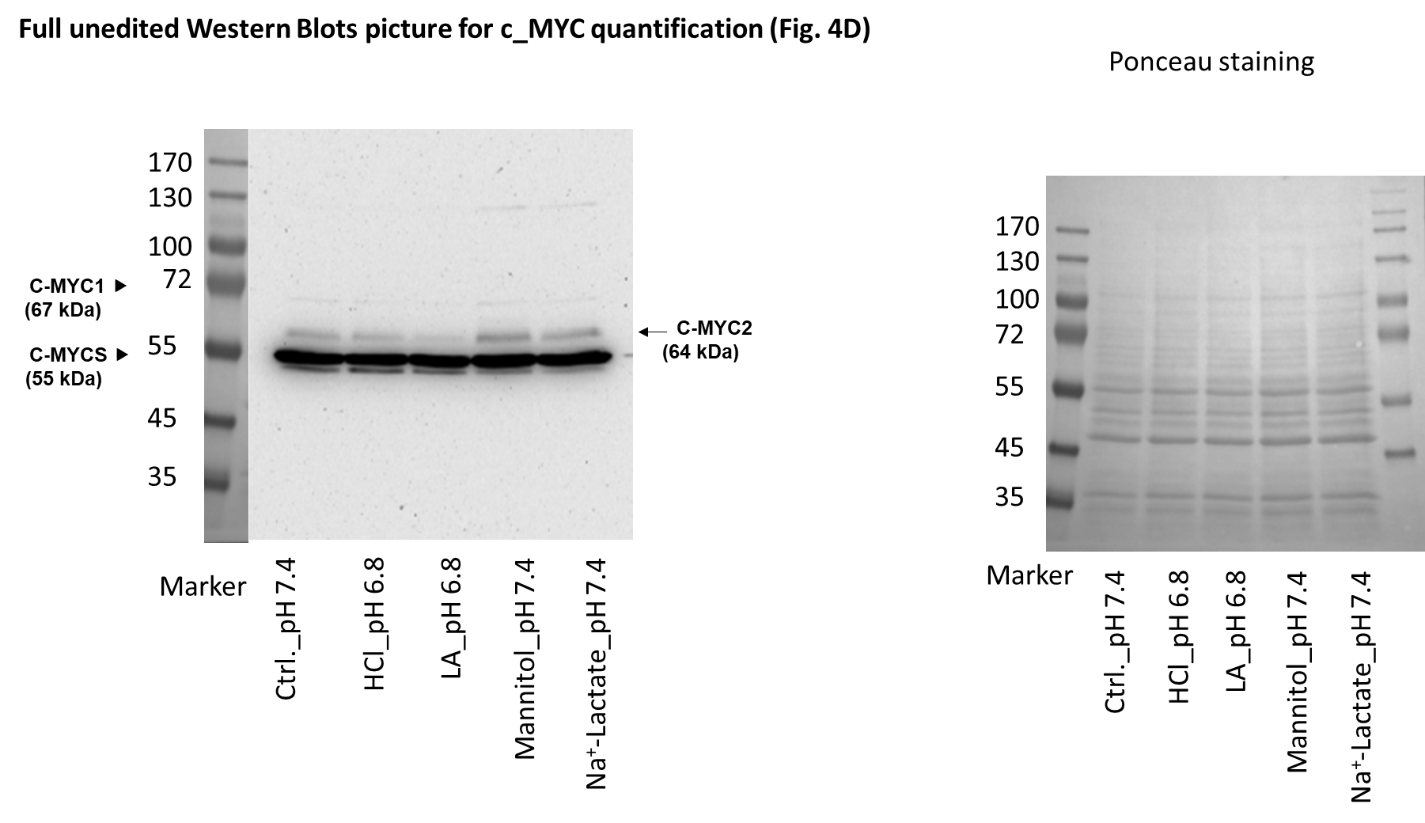
**

**
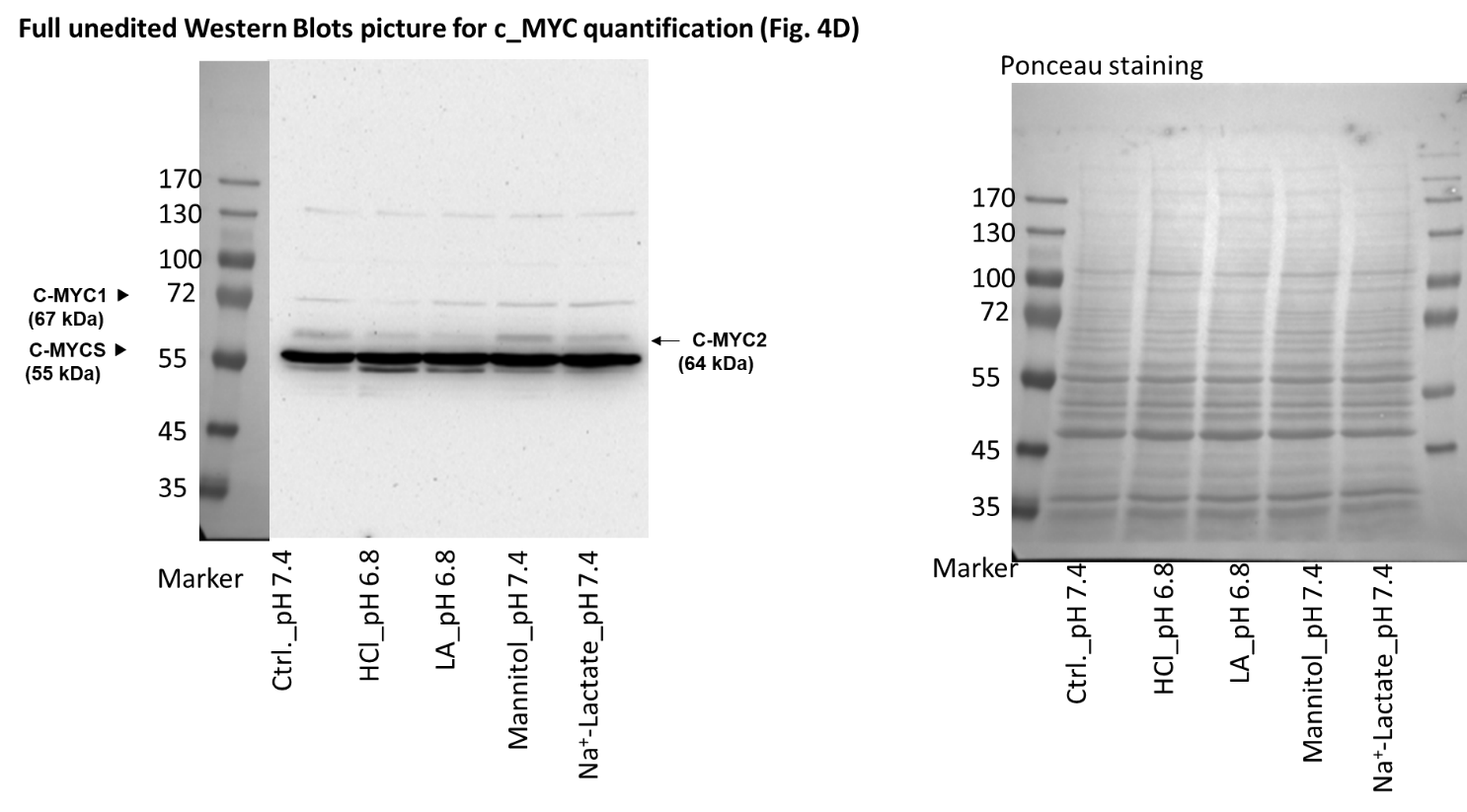
**

**
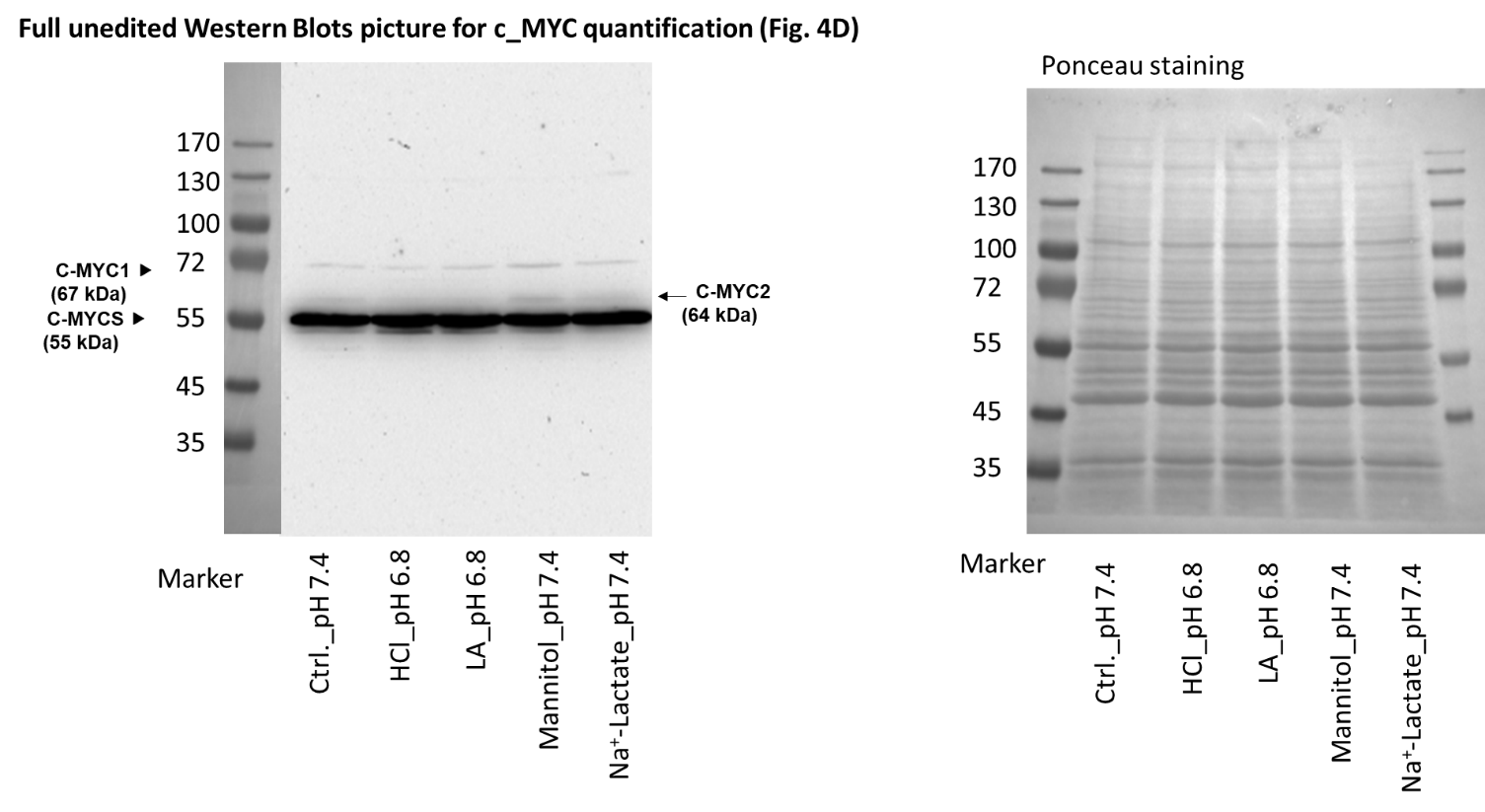
**
